# Achieving the “Ending the HIV Epidemic in the U.S.” incidence reduction goals among at-risk populations in the South

**DOI:** 10.1186/s12889-023-15563-5

**Published:** 2023-04-20

**Authors:** Deven T. Hamilton, Karen W. Hoover, Dawn K. Smith, Kevin P. Delaney, Li Yan Wang, Jingjing Li, Tamika Hoyte, Samuel M. Jenness, Steven M. Goodreau

**Affiliations:** 1 Center for Studies in Demography and Ecology, University of Washington, 206 Raitt Hall, UW, Box 353412, Seattle, WA 98195-3412 USA; 2 Division of HIV Prevention (DHP), National Center for HIV, Viral Hepatitis, STD, and TB Prevention (NCHHSTP), Centers for Disease Control and Prevention (CDC), Atlanta, GA USA; 3 Division of Adolescent and School Health, Centers for Disease Control and Prevention, Atlanta, GA USA; 4 National Center for HIV, Viral Hepatitis, STD, and TB Prevention (NCHHSTP), Centers for Disease Control and Prevention (CDC), Atlanta, GA USA; 5 Department of Epidemiology, Rollins School of Public Health, Emory University, Atlanta, GA USA; 6 Department of Anthropology, University of Washington, Seattle, WA USA

**Keywords:** HIV, Antiretroviral therapy, Pre-exposure prophylaxis, Modeling

## Abstract

**Introduction:**

Antiretroviral medication coverage remains sub-optimal in much of the United States, particularly the Sothern region, and Non-Hispanic Black or African American persons (NHB) continue to be disproportionately impacted by the HIV epidemic. The “Ending the HIV Epidemic in the U.S.” (EHE) initiative seeks to reduce HIV incidence nationally by focusing resources towards the most highly impacted localities and populations. This study evaluates the impact of hypothetical improvements in ART and PrEP coverage to estimate the levels of coverage needed to achieve EHE goals in the South.

**Methods:**

We developed a stochastic, agent-based network model of 500,000 individuals to simulate the HIV epidemic and hypothetical improvements in ART and PrEP coverage.

**Results:**

New infections declined by 78.6% at 90%/40% ART/PrEP and 94.3% at 100%/50% ART/PrEP. Declines in annual incidence rates surpassed 75% by 2025 with 90%/40% ART/PrEP and 90% by 2030 with 100%/50% ART/PrEP coverage. Increased ART coverage among NHB MSM was associated with a linear decline in incidence among all MSM. Declines in incidence among Hispanic/Latino and White/Other MSM were similar regardless of which MSM race group increased their ART coverage, while the benefit to NHB MSM was greatest when their own ART coverage increased. The incidence rate among NHB women declined by over a third when either NHB heterosexual men or NHB MSM increased their ART use respectively. Increased use of PrEP was associated with a decline in incidence for the groups using PrEP. MSM experienced the largest absolute declines in incidence with increasing PrEP coverage, followed by NHB women.

**Conclusions:**

Our analysis indicates that it is possible to reach EHE goals. The largest reductions in HIV incidence can be achieved by increasing ART coverage among MSM and all race groups benefit regardless of differences in ART initiation by race. Improving ART coverage to > 90% should be prioritized with a particular emphasis on reaching NHB MSM. Such a focus will reduce the largest number of incident cases, reduce racial HIV incidence disparities among both MSM and women, and reduce racial health disparities among persons with HIV. NHB women should also be prioritized for PrEP outreach.

**Supplementary Information:**

The online version contains supplementary material available at 10.1186/s12889-023-15563-5.

## Introduction

There are now several effective interventionstrategies using antiretroviral medications to prevent HIV transmission/acquisition, including antiretroviral therapy (ART) for treatment, treatment as prevention (TasP) and pre-exposure prophylaxis (PrEP). Unfortunately, antiretroviral medication coverage remains low in much of the United States [1–3]. The 16 states and District of Columbia that make up the South census region currently experience the greatest burden of HIV of any U.S. region, and lag behind in providing quality HIV prevention services and care [3]. The CDC has estimated that of the 34,800 new HIV infections in persons > = 13 years in 2019, 18,500 (53%) were in the South [4]. The South also had a prevalence rate (524.4 per 100,000 persons) far higher than either the West, or Midwest with rates of 364.7 and 252.9 per 100,000 persons, respectively.

In the US, and the South, Non-Hispanic Black or African American persons (NHB) continue to be disproportionately impacted among both gay, bisexual, and other men who have sex with men (collectively referred to as MSM) and heterosexually active men and women. NHB women in particular have experienced a substantial HIV disparity [5]— As of 2018, HIV prevalence among NHB women was estimated to be 17 times that of White women [5].

The “Ending the HIV Epidemic in the U.S.” (EHE) initiative seeks to reduce HIV incidence nationally by 75% by 2025 and 90% by 2030. Introduced in 2019, EHE focuses federal resources towards the most highly impacted localities and populations, including the South, to improve access and uptake of effective interventions [6]. A key step in achieving EHE goals will be increasing antiretroviral medication coverage, both TasP and PrEP, to reduce transmission/acquisition, but the combinations and levels of TasP and PrEP needed to meet the EHE goals in the South are unknown. It is also not clear how different patterns of use, by race and gender, may affect disparities in HIV.

Prior studies have estimated the levels of ART and PrEP coverage needed to reach the EHE goals among some specific sub-populations. A study of MSM in Atlanta [7] concluded that it may be possible to reach the EHE goals of a 75% and 90% reductions in incidence by 2025 and 2030 respectively, with immediate and substantial improvements in HIV screening, PrEP use, and ART care and retention; however, meeting these goals will require specifically addressing the HIV service needs of black MSM [7]. Similarly, a study of city-level HIV transmission for 32 priority metropolitan statistical areas (MSAs) in the U.S found that improving HIV testing, ART coverage, and PrEP coverage among all MSM and persons who inject drugs could reduce incidence by 48% to 90% and that 32 MSAs could achieve greater than 90% reductions in HIV incidence with large-scale interventions that include heterosexual men and women [8]. But thus far no studies have estimated the level of ART and PrEP coverage that will be needed to meet the EHE goals in the South. The EHE goals are national in scope, but here we focus specifically on the South where the HIV burden is greatest, and where the epidemic is a significant cause of morbidity and mortality among MSM and heterosexuals, particularly NHB women. Without significant reductions in new infections in the South the EHE goals will not be attainable. In addition, given the significant racial disparities in HIV burden found in the South, achieving the EHE incidence reductions goal in the South would also represent a significant step towards reducing HIV-related disparities and health inequities, one of the four goals of the National HIV/AIDS Strategy (2022–2025) [9].

Here we conduct a series of counterfactual analyses that explore the overall ART and PrEP coverage levels needed in the South to achieve EHE goals, as well as the impact of different levels of uptake by specific population segments defined by race, sex, and sex of sexual contacts on HIV disparities, focusing on outcomes among racial/ethnic minority populations that are disproportionately affected by HIV.

## Methods

We developed a stochastic, agent-based network model to simulate the HIV epidemic in the South. The model was built using the EpiModel [10] and *statnet* [11–13] software platforms. The extension EpiModelHIV R package was used to incorporate the relevant HIV-specific epidemiology for this study [https://github.com/EpiModel/EpiModelHIV]. Similar models have been used previously to model HIV and STI interventions and outcomes in other contexts [7, 14, 15]. Full details of the model and parameters are in the technical appendix (Supplemental Material 1), but we provide a brief overview here.

The simulation included 500,000 individuals ages 15–65, with the age, sex, and race composition of the South from the US census. The population was 50.6% women, 45.9% men with exclusively opposite sex partners and 3.6% MSM [16]. Women who have sex with women were not included in the simulation given their low HIV burden. The racial composition of the simulated population was collapsed to three categories: NHB, Hispanics (of any race), and all others, in order to focus on outcomes for NHB and Hispanics, priority populations identified in the National HIV/AIDS Strategy 2022–2025 [9]. The proportion of men who have sex with men and women (MSMW) varied by race/ethnicity (26.4%, 12.4% and 10.9% for NHB, Hispanic and White/Other MSM, respectively), following Dasgupta et al. 2020. [17] We used three waves of the National Survey of Family Growth (NSFG) (2011–2017) [18–20] to estimate sexual network and behavioral parameters for men and women with opposite sex partners, and ARTnet [21] to obtain equivalent data for MSM. The NSFG is a nationally representative survey of U.S. households with independent samples of men and women ages 15–49. The ARTnet study was an anonymous cross-sectional web-based survey conducted in the United States in 2017–2019 that focused on HIV-related behaviors and prevention among MSM ages 15–65 [21].

We modeled networks of three interacting types of MSM and heterosexual sexual relations: main partnerships, casual (but persistent) partnerships, and one-time sexual contacts. All six networks included the same node set (individuals) but the ties on each network were determined by the type of partnership. At each time step, individuals could form or dissolve one or more partnerships. Each partnership had type-specific within-partnership behaviors (e.g., HIV status disclosure, condom use, coital frequency, sexual role (for MSM)). Partnerships had duration specific to both the type of partnership and the age of the nodes. Mixing patterns were age-, race-, and sex-specific within partnership type. Partnership formations of each type were also conditional on the presence of partnerships of other types. Demographic change included entry at age 15, aging, mortality, and exit due to mortality or aging out of the population. Intrahost HIV epidemiology included the natural progression of disease within persons with HIV (PWH) in the absence of clinical intervention. Changes in HIV viral load were associated with immunological disease progression and HIV transmission rates. The clinical epidemiological processes in the model included HIV screening, linkage to care, treatment, PrEP initiation, adherence to either ART or PrEP, and HIV viral suppression. The epidemic simulation was run for the 8 years remaining in the EHE initiative (2022 – 2030).

Outcomes of interest were the total number of infections averted over the remaining 8 years of EHE, the simulated annual HIV incidence rate at year 5 (2025) and year 10 (2030) of EHE, the reduction in incidence relative to 2030 incidence in the reference scenario, the number of infections averted per 100,000 person-years at risk (NIA) and the percentage of infections averted (PIA). All outcomes were reported as means over 50 simulations and 80% simulation intervals (SI), the range of the central 80% of simulations. For all analyses, men that had both male and female partners were included in the MSM categories for both the intervention groups and the outcomes.

In the simulations, ART coverage was a stochastic function of testing, linkage to care, retention in care, discontinuation and re-initiation, all of which varied by demographic group. In scenarios with fixed ART coverage, PWH in each demographic group targeted by the intervention were selected at random to initiate ART such that the coverage within each demographic group was equal to the fixed coverage level. Individuals initiating ART as part of the hypothetical intervention were also assigned the highest level of adherence conferring a 0.01 relative risk of HIV acquisition given exposure under the assumption that the hypothetical intervention used to achieve the target ART coverage also increase adherence. Throughout the simulation the remainder of the HIV care continuum continued to operate as it does under the reference scenario with individuals testing, beginning treatment, and adhering to, discontinuing, and reinitiating care. Individuals starting ART through this empirically derived pathway also adhered to ART based on the empirically derived estimates of adherence. If through this background pathway the proportion of individuals on ART exceeded the fixed coverage, individuals on ART were randomly selected to discontinue ART.

In the reference scenario 64%, 68%, and 68% of NHB, Hispanic and White/Other PWH MSM were on ART while 50%, 21%, 30%, 41%, 27% and 22% of NHB, Hispanic and White/Other heterosexual men and women with HIV were on ART. These coverage rates were simulated outcomes of demographic-group specific testing, linkage to care and retention rates. In the intervention scenarios ART coverage was systematically varied across fixed levels from 60 to 100%. Consequently, scenarios in which ART coverage was fixed at 60% for MSM reflected a decrease in ART coverage in this population. The 60% coverage rate was selected as our lower bound because it is the closest round value to current MSM coverage rates without exceeding them and thereby reflects an approximation for getting the other 6 demographic groups to ART coverage similar to current coverage among MSM. The increases in incidence observed when 60% ART coverage is applied to the MSM can be interpreted as the potential outcomes if ART coverage declines with a shift in focus to other public health priorities.

PrEP use was modeled stochastically in the reference scenario. MSM in the simulation were indicated for PrEP if they were HIV-negative and either in a monogamous partnership with a partner who had not tested in the past 6 months, or they were in 2 or more partnerships. Heterosexuals in the simulation were indicated if they were HIV-negative and either in a casual partnership with a partner who had not HIV-tested in 6 months, or they were in 2 or more partnerships. In our model, PrEP was only indicated for individuals age 18 and older.

The level of PrEP coverage was fixed in the reference scenario and was based on findings from Siegler et al. [22] who reported 21 PrEP users per 100,000 individuals in the South in 2017 overall, and 1.9/100,000 among women. We assumed that the rate of PrEP use among heterosexual men more closely resembles that of women than MSM and used the 1.9/100,000 rate for heterosexual men as well. After determining the expected number of heterosexual men and women on PrEP using these rates, we subtracted the heterosexuals from the total number of reported PrEP users and used the remainder to determine the rate for MSM (1351.2/100,000). To account for differences in PrEP use by race we used the proportions of indicated individuals currently using PrEP reported in the HIV Surveillance Supplemental report [23]; (5.9% for NHB, 10.9% for Hispanic, 42.1% for White/Other) and applied the relative proportions to the three different race groups for the MSM, heterosexual men and heterosexual women. PrEP adherence [24, 25] and discontinuation [24, 26] also varied by demographic group, and the full details of the PrEP continuum are in the technical Appendix.

In the intervention scenarios PrEP was fixed within each scenario at 10%—50% coverage for those indicated for PrEP. At each timestep in the simulation PrEP eligibility was determined for those not currently indicated for PrEP and reassessed annually for those already indicated. If coverage was below the fixed coverage level, indicated individuals were randomly selected from each demographic group to initiate PrEP. PrEP discontinuation occurred both stochastically and with a change in indications.

We conducted two analyses (Table 1). The first estimated the impact of increases in ART and PrEP coverage on the EHE goals. The second examined the demographic group-specific outcomes associated with demographic group-specific changes in ART and PrEP coverage individually. For reference each scenario is numbered in Table 1.

**Table 1:** Analyses exploring the potential impact of changes in ART and PrEP coverage on achieving EHE goals and their implications for the racial and sex disparities in HIV burden

Analysis	Scenario	ART coverage among HIV positive persons	PrEP coverage among indicated persons	Intervention population
1	1	60%	10%	All
	2	70%	20%	
	3	80%	30%	
	4	90%	40%	
	5	100%	50%	
	6	90%	10%	
	7		20%	
	8		30%	
	9		40%	
	10		50%	
	11	95%	10%	
	12		20%	
	13		30%	
	14		40%	
	15		50%	
2	16–20	60%-100%	Baseline	Black/African American MSM
	21–25			Hispanic/Latino MSM
	26–30			White/Other MSM
	30–35			Black/African American heterosexual males
	36–40			Hispanic/Latino heterosexual males
	41–45			White/Other heterosexual males
	46–50			Black/African American females
	51–55			Hispanic/Latino females
	56–60			White/Other females
	61–65	Baseline	10%-50%	Black/African American MSM
	66–70			Hispanic/Latino MSM
	71–75			White/Other MSM
	76–80			Black/African American heterosexual males
	80–85			Hispanic/Latino heterosexual males
	86–90			White/Other heterosexual males
	91–95			Black/African American females
	96–100			Hispanic/Latino females
	101–105			White/Other females

## Results

**Fig. 1:**
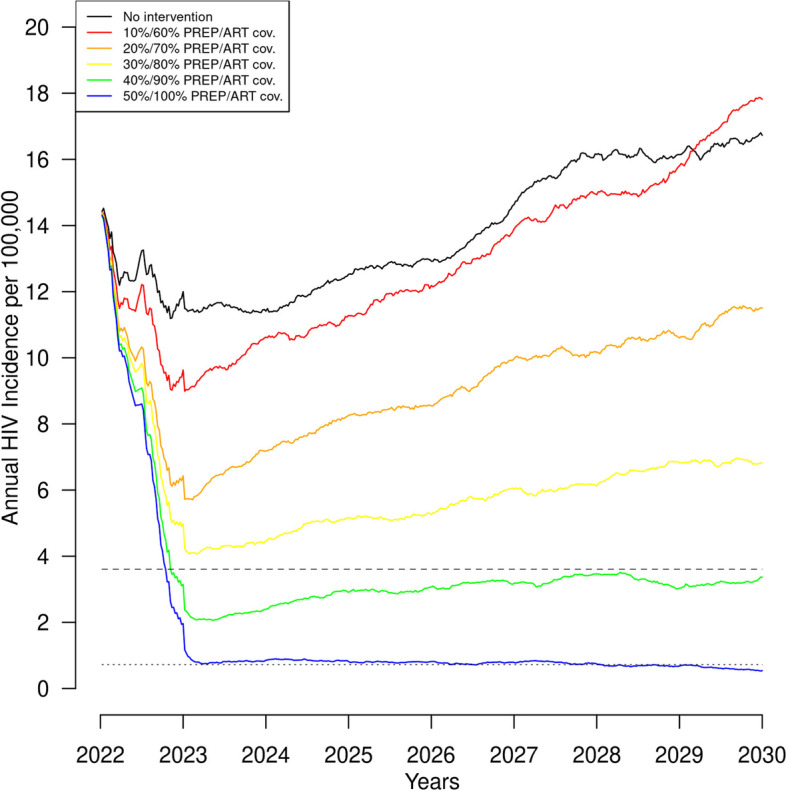
Annual HIV incidence per 100,000 individuals over 8 years with simultaneously improved antiretroviral therapy (ART) and preexposure prophylaxis (PrEP) coverage. Footnote: Dashed lines indicate the 75% (large dashes) and 90% (small dashes) EHE goals; An alternative version of Fig. 1 with shaded regions for the SI is included in the supplemental material

In our reference scenario with baseline ART and PrEP coverage, there were 549.7 (80%SI: 487.8, 600.8) new HIV infections over 8 years, and 48% (80%SI: 47%, 49%) of PWH on ART were fully virally suppressed. Figure 1 shows the annual incidence per 100,000 individuals from the reference scenario and scenarios with interventions that increased both ART and PrEP simultaneously for the entire population uniformly. The simultaneous increases resulted in a roughly linear decline in the total number of new HIV infections among 100,000 individuals over 8 years from 549.7 (80%SI: 487.8, 600.8) to 517.6 (80%SI: 462.6, 566.7) at 60%/10% ART/PrEP coverage to 116.9 (80%SI: 99, 140.3) at 90%/40% ART/PrEP coverage (Table 2). Total new infections declined by 78.6% at 90%/40% ART/PrEP and 94.3% at 100%/50% ART/PrEP. The decline in the annual incidence rate surpassed the EHE target of 75% by 2025 with 90%/40% ART/PrEP and the EHE target of 90% by 2030 with 100%/50% ART/PrEP (Fig. 1). The rapid decline in the incidence rates observed in each of the intervention scenarios is the result of the instantaneous change in ART and PrEP coverage at the beginning of the simulation. Transmission persisted even at 100% ART coverage due to imperfect ART adherence. In our reference scenario the proportion of PWH who were on ART and fully suppressed ranged from a high of 61% among White/Other MSM to a low of 14% among Hispanic/Latino heterosexual men. In our simulated scenario with 100% ART coverage 94% of all PWH were fully suppressed.

**Table 2:**
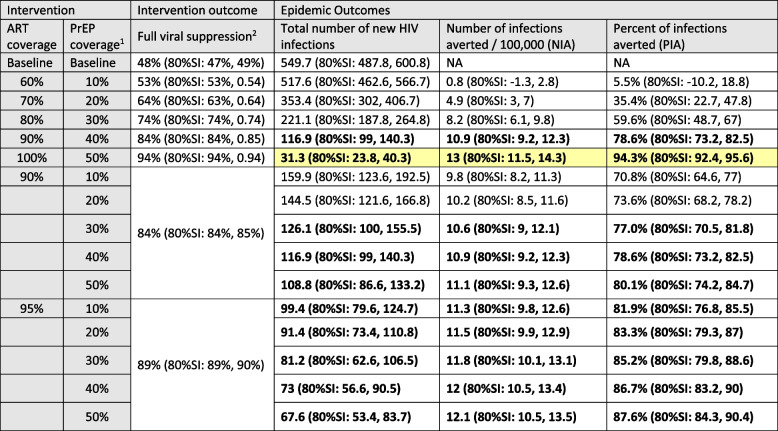
Reductions in estimated new HIV infections associated with universal increasing viral suppression through increased ART coverage and HIV prevention through PrEP uptake

*ART* Antiretroviral therapy, *PrEP*: Pre-exposure prophylaxis, *SI* Simulation interval

^1^Prep coverage is calculated using the number of individuals eligible for PrEP as the denominator

^2^The percentage fully suppressed is calculated as the fraction of all HIV positive individuals on ART with a viral load < = 200. The hypothetical interventions increases overall ART coverage and assumed those newly enrolled in ART are fully compliant and become fully suppressed. The percentage fully suppressed is lower than the ART coverage due to those already on but not fully suppressed at the time the intervention is implemented and individuals initiating ART but who have not been on ART long enough to reach full suppression

Reduction in new HIV infections of > = 75% are shown in bold

Reduction in new HIV infections of > = 90% are highlighted

Achieving 60%/10% ART/PrEP coverage had divergent effects on different demographic groups. For MSM annual incidence increased for NHB, Hispanic/Latino and White/Others by 8.7%, 14.4% and 3.1% respectively (Table 3). The negative ramifications for these populations were due to the higher overall ART coverage at baseline. Essentially, moving to 60% ART coverage represents a regression in the continuum of care for MSM and therefore an increase in incidence. Conversely 60%/10% ART/PrEP coverage is a significant improvement for heterosexuals. For heterosexual men incidence declined by 13.8%, 20.2% and 61.0% for NHB, Hispanic/Latino and White/Others respectively (Table 4). At 100%/50% ART/PrEP coverage incidence declined by 92.9%, 93.4%, and 100% for NHB, Hispanic/Latino and White/Others heterosexual men respectively. The largest reductions were among White/Other heterosexual men when considering percentage declines, but among NHB MSM when considering absolute declines. This reflects the extremely low baseline HIV rates among some groups (e.g., White heterosexual men), which provides little room for absolute declines, and for whom a very small absolute decline can represent a large relative decline.

**Table 3:**
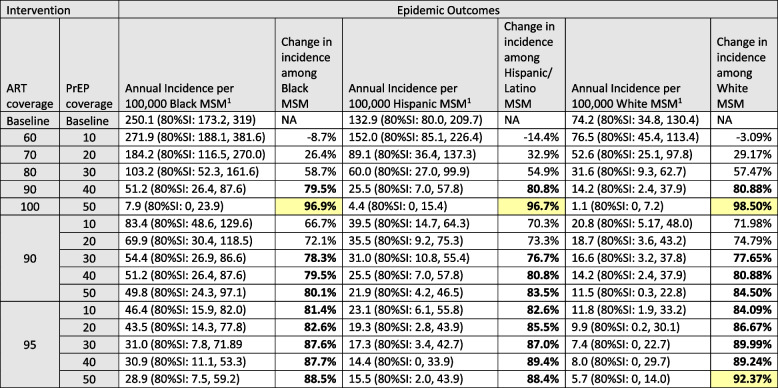
Absolute and relative reductions in estimated HIV incidence rates among MSM associated with universal increasing viral suppression through increased ART coverage and HIV prevention through increased PrEP uptake

*ART* Antiretroviral therapy, *PrEP* Pre-exposure prophylaxis, *SI* Simulation interval

^1^Incidence calculations for MSM include males who have sex with both males and females

Changes in incidence rate of > = 75% are shown in bold

Changes in incidence rate of > = 90% are highlighted

**Table 4:**
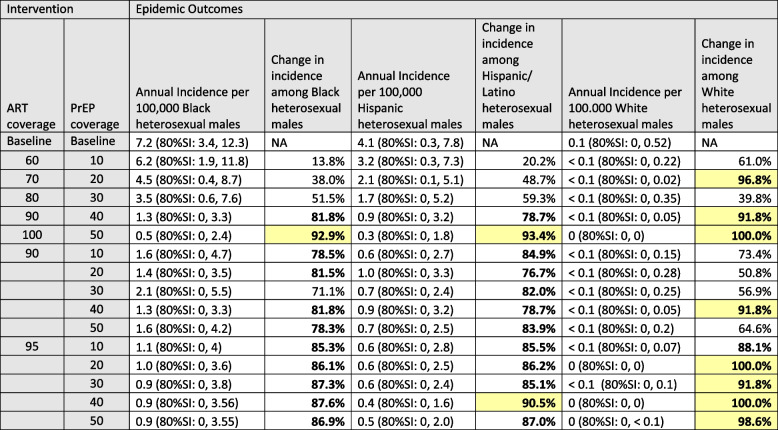
Absolute and relative reductions in estimated HIV incidence rates among heterosexual males associated with universal increasing viral suppression through increased ART coverage and HIV prevention through increased PrEP uptake

*ART* Antiretroviral therapy, *PrEP* Pre-exposure prophylaxis, *SI* Simulation interval

Changes in incidence rate of > = 75% are shown in bold

Changes in incidence rate of > = 90% are highlighted

The outcomes for heterosexual women were similar to those for heterosexual men when coverages were improved uniformly across all groups (Table 5). Incidence among women declined approximately linearly as both ART and PrEP coverage increased. At 100%/50% ART/PrEP coverage incidence among White/Other women reached 0 (80% SI: 0,0), a 100% decline in incidence. In absolute terms the reduction among White/Other women was just 0.2/100,000 individuals, contrasting with a decline among NHB women from 14.4 (80%SI: 7.3, 21.0) to 0.9/100,000 individuals (80%SI: 0, 3.2), a decline of 13.5/100,000 individuals. Following the same pattern as the heterosexual men, White/Other women’s lower bound of the SI was 0 even at baseline but realizations with no incident cases emerged for Hispanic/Latino women at 80%/30% ART/PrEP coverage and for NHB women at 90%/40% ART/PrEP coverage.

**Table 5:**
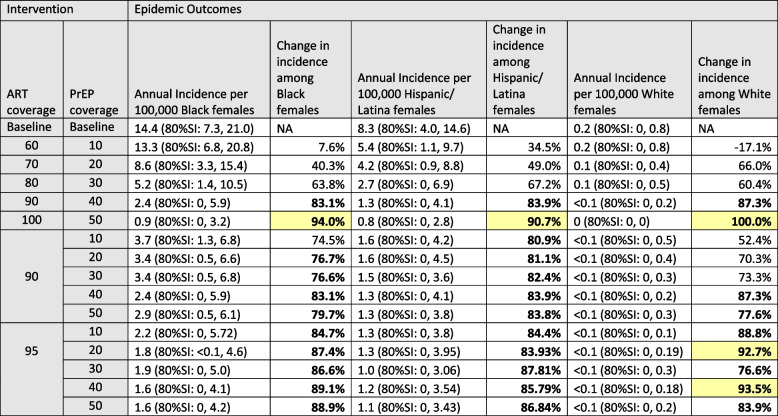
Absolute and relative reductions in estimated HIV incidence rates among heterosexual females associated with universal increasing viral suppression through increased ART coverage and HIV prevention through increased PrEP uptake

*ART* Antiretroviral therapy, *PrEP* Pre-exposure prophylaxis, *SI* Simulation interval

Changes in incidence rate of > = 75% are shown in bold

Changes in incidence rate of > = 90% are highlighted

In scenarios 6–10, we fixed ART coverage at 90% and adjusted PrEP coverage from 10 to 50% (Table 2). With 90% ART coverage and just 10% PrEP coverage overall incidence declined by 70.8% and the NIA was 9.8/100,000 individuals (80%SI: 8.2, 11.3). Improving PrEP coverage to 50% in conjunction with 90% ART coverage reduced overall incidence by 80.1% and increased the NIA to 11.1/100,000 individuals (80%SI: 9.3, 12.6). ART coverage increased from 90 to 95% in scenarios 11–15. At 10% PrEP coverage the 5% increase in ART coverage had a similar, slightly larger, effect than increasing PrEP from 10 to 50% coverage with persistent 90% ART coverage, reducing overall incidence by 81.9% and increasing the NIA to 11.3/100,000 individuals (80%SI: 9.8, 12.6).

**Fig. 2:**
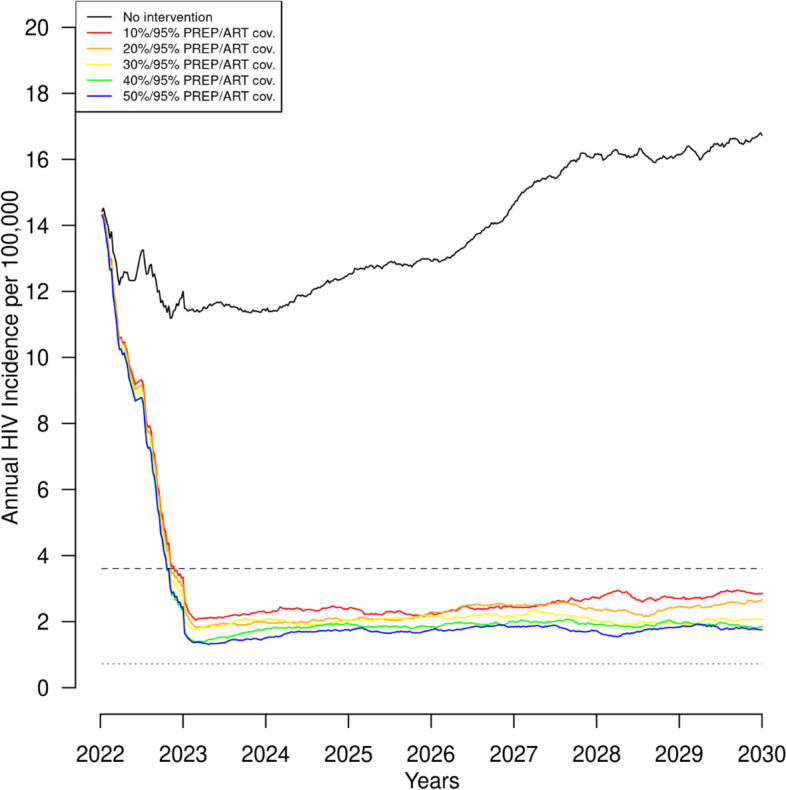
Annual HIV incidence per 100,000 individuals over 8 years with 95% antiretroviral therapy (ART) and hypothetical preexposure prophylaxis (PrEP) coverage improvements from 10 to 50%. Footnote: Dashed lines indicate the 75% (large dashes) and 90% (small dashes) EHE goals

Figure 2 shows annual incidence over the 8 years of simulation with 95% ART coverage and PrEP use from 10 to 50%. The overall contribution of PrEP to reducing incidence is small relative to the impact of high ART coverage. With such high ART coverage, the short term EHE goal of a 75% reduction in incidence is achievable regardless of the level of PrEP coverage, but even at 50% PrEP use the reductions in incidence did not reach the longer-term goal of 90%.

In our final set of scenarios, we increased ART and PrEP coverage independently for each of the 9 demographic groups and evaluated the change in incidence for the 9 groups separately. The results of these analyses are in the supplemental Appendix. When we increased ART coverage among NHB MSM we found a linear decline in incidence among NHB MSM as well as a significant decline in incidence among Hispanic/Latino and White/Other MSM. When we increased ART coverage among either Hispanic/Latino or White/Other MSM we also found a linear decline in incidence among all three MSM groups. However, the declines in incidence among Hispanic/Latino and White/Other MSM were almost identical regardless of which MSM race group increased their ART coverage, while the benefit to NHB MSM was greatest when their own ART coverage increased. Increases in PrEP use among the three different MSM groups significantly reduced incidence among the group receiving the greater PrEP coverage but even at 50% coverage there was little prevention benefit in reducing incidence in any of the other demographic groups.

The pattern among Hispanic/Latino and White/Other heterosexuals was quite similar. Increased ART coverage among each sex/race group was accompanied by a significant decline in incidence among members of the opposite sex within the same race group. There were also modest reductions in incidence among the group increasing their ART use, suggesting a disruption in chains of forward transmission.

**Fig. 3:**
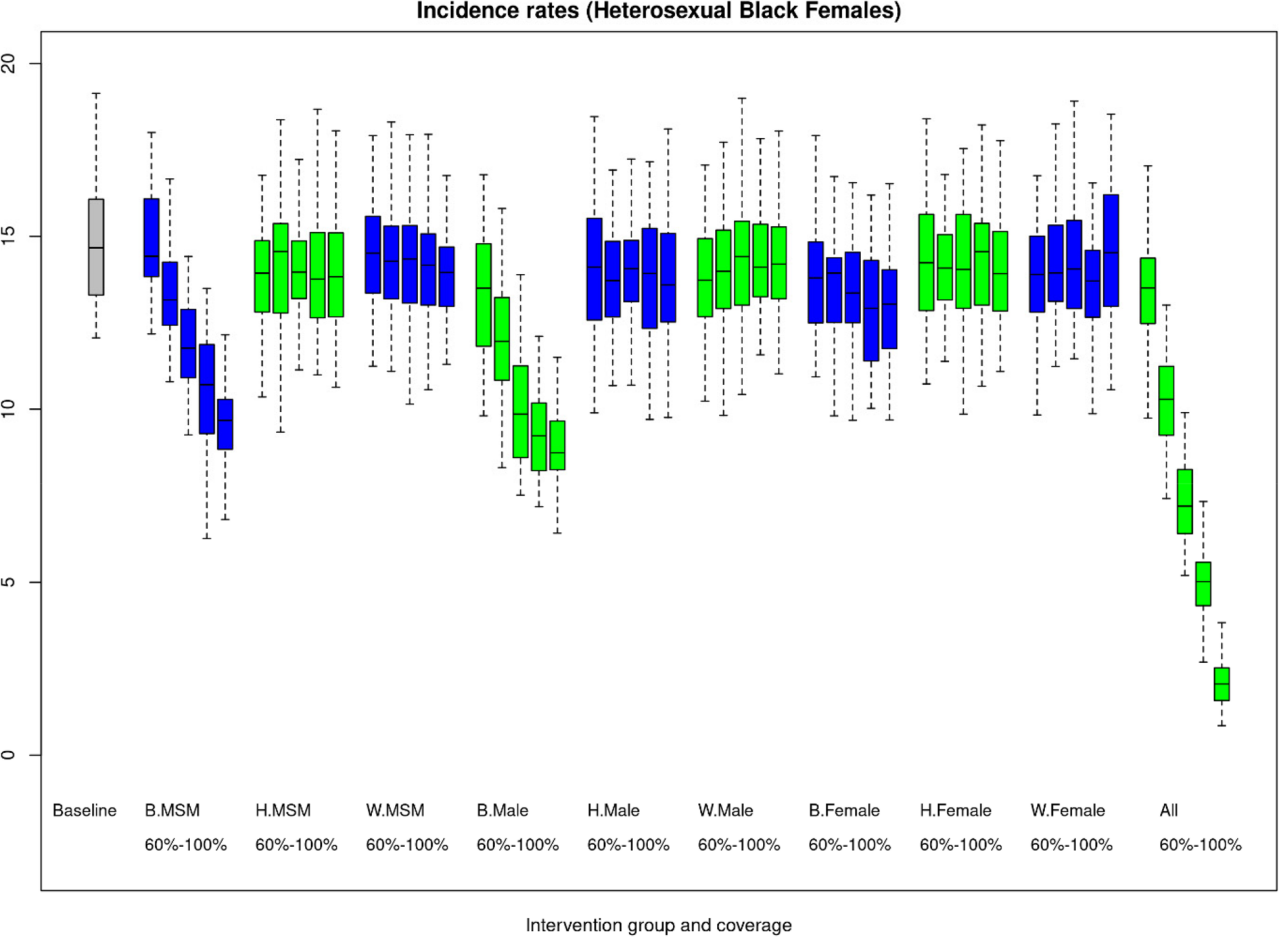
HIV incidence rates among non-Hispanic Black heterosexual females with hypothetical improvements in antiretrovirus (ART) coverage from 60 to 100% within 9 individual demographic groups and all combined. Note: The reference scenario is shown in grey. The blue and green color coding is to indicate a change in the intervention population moving from left to right. Each boxplot is the combined results for 50 simulations and represents outcomes for heterosexual non-Hispanic Black females. In the x-axis labels B, H, and W indicate non-Hispanic Black, Hispanic/Latino and White/Other respectively. The male category indicates heterosexual males

The patterns among the NHB heterosexuals were unique. Figure 3 shown the changes in incidence among NHB women when different segments of the population increase their ART use. They experience little reduction in incidence when they improve their own rate of ART use even to 100%. The incidence rate among NHB women declined by over a third, from 14.4/100,000 (80%SI: 7.3, 21.0) to 9.0/100,000 (80%SI: 3.8, 15.8) and 9.2/100,000 (80%SI: 4.6, 14.6), when either NHB heterosexual men or NHB MSM increased their ART use respectively. There was also a modest downward trend in incidence among NHB women as ART coverage improved among Hispanic/Latino and White/Other MSM. NHB heterosexual men also showed no reduction in incidence when they increased their ART use even to 100% (figure S5). They experienced the largest declines in incidence with increased ART coverage among NHB women but also a significant reduction in incidence as ART coverage increased among NHB MSM.

For all 9 demographic groups, increased use of PrEP was associated with a decline in incidence for the groups using PrEP. MSM experienced the largest absolute declines in incidence with increasing PrEP coverage, followed by NHB women (see supplemental figures S11-S19). The reductions associated with PrEP were small relative to improvement in ART coverage even at 50% PrEP coverage, and we found no indication of reductions outside the group using PrEP that might result from fewer prevalent cases in the population.

## Discussion

In this simulation study we analyzed the potential of improved rates of ART and PrEP use to reduce HIV incidence in the Southern U.S., testing whether these improvements were sufficient to meet the EHE goals of a 75% reduction by 2025 and a 90% reduction by 2030. Our model included heterosexuals, MSM and MSMW, six interacting sexual networks, nine partnership types and three race groups allowing us to capture transmission dynamics within and between these groups to evaluate how ART and PrEP uptake in different populations may impact sex and racial/ethnic disparities in HIV.

Consistent with other studies [7, 8] our results suggest that the near term EHE goals can be achieved if ART coverage is increased to 95% of PWH with at least 89% fully suppressed. If ART coverage only improves to 90% of PWH with 84% full viral suppression PrEP coverage will need to be expanded to cover > 20% of those indicated. Our results also indicate that improvements in ART coverage and PrEP to 100% and 50% respectively could reduce HIV incidence by almost 95% by 2030 if 94% of those on ART are fully virally suppressed.

In addition, if ART and PrEP were expanded uniformly across the entire population the percent reductions in incidence would be greatest among White/Other heterosexuals who currently have the lowest HIV burden, but the absolute reductions in incidence would be greatest among MSM and NHB women.

Our results also highlight the role of sexual networks and the way in which individuals are interconnected drive both the epidemic and the dynamics of prevention. Among MSM, increases in ART use by individuals of any race group produced net positive outcomes for all MSM. In fact, for Hispanic/Latino and White/Other MSM the reductions in incidence were similar irrespective of the race group increasing ART coverage. For NHB MSM however, the reductions in incidence were greater when ART coverage increased among NHB compared to similar increases among Hispanic/Latino and White/Other MSM. This suggests that focusing resources on improving ART coverage among NHB MSM will have the greatest impact on reducing incidence among NHB MSM, reducing racial disparities among MSM, and have no less of an impact on reducing incidence among other MSM relative to allocating resources toward different racial and ethnic groups.

Heterosexuals by and large saw significant reductions in incidence only when individuals of the same race but opposite sex increased their use of ART. However, NHB women were an important exception to this general pattern. NHB women are a significant and often overlooked population with unique challenges for HIV prevention including access to health care and other supportive services that enable women to exercise agency in their own HIV prevention and care [27]. Our finding suggest that NHB women in particular face a challenge based on the structure of sexual relations and the underlying patterns of sexual behavior in the community as a whole. NHB women were unique in that their HIV outcomes were impacted the least by changes in their own ART use and by far the most by changes in ART use by others. There was little decline in incidence with improved ART and viral suppression, but for all other groups we found that there was a reduction in incidence among sexual partner groups and second-order reductions in incidence among those using ART. In addition, the outcomes for NHB women were highly impacted by changes in ART use by multiple other groups. Incidence among NHB women declined to the same degree when NHB heterosexual men or MSM increased their ART use and also experienced small reductions in incidence with improved ART among non-NHB MSM.

The pattern observed among NHB women highlights just how interconnected the population is and how much HIV outcomes at the individual level depend not just on individual behavior but the structure of transmission networks. However, increasing PrEP use did reduce incidence among NHB women by a greater degree than similar PrEP use among any other heterosexual groups suggesting that improved PrEP use among NHB women would reduce a significant number of infections in this group, reduce racial disparities in HIV outcomes, and have a much larger overall impact on the epidemic than increased PrEP uptake among other heterosexual groups.

It should also be noted that MSM were a significant part of the overall HIV epidemic through their partnerships with heterosexual women. One recent study found that a majority of new infections among heterosexual women in the US originated among MSM [28] which is consistent with recent findings in Switzerland [29]. Our results suggest the same. We found significant reductions in incidence among MSM as well as among heterosexuals, particularly NHB women with improved ART coverage among MSM. It will be imperative to continue to improve ART among MSM where the impact on transmission and the utility of TasP is greatest. However, this should not overshadow the need for improved testing and linkage to care among heterosexuals as all those infected are in equal need of treatment. Expanded PrEP will also be an important tool for those at risk to protect themselves.

There were several limitations to this study. First, in our reference scenario incidence is projected to increase over the next 8 years, all else being equal. This is contrary to recent trends which have shown that HIV diagnoses have decreased for all risk groups in 2009–18; and among MSM, new diagnoses decreased both overall and for White MSM, remained stable for Black MSM, and increased for Hispanic/Latino MSM [3]. Other projection models have similarly pointed to general declines in incidence over the coming years [8]. Our divergent findings may be the result of our calibration method. We calibrated our model to current prevalence and incidence assuming that current reported behavior and the continuum of care as they exist now were in place during the entire history of the epidemic rather than something that co-evolved with the epidemic. Given these additional barriers to transmission at the outset our model may overestimate transmission and consequently underestimate the impact of additional viral suppression and PrEP use. Second, we used a very broad definition of PrEP eligibility based on the CDC guidelines, and the result was that approximately 30% of the population was indicated for PrEP at any time. This is in line with recent estimated for MSM [30] but may be a substantial over-estimate for heterosexual populations. Given the size of the heterosexual population, this suggests that PrEP use would need to be very widespread in terms of the number of individuals on PrEP in order to have a meaningful impact on HIV incidence, which may not be practical or cost-effective. There is no doubt that PrEP will be an important tool in reaching the EHE goals of a 90% reduction in incidence, but its use may need to be more targeted than the guidelines we used for this study. PrEP should certainly be available for those that perceive themselves as at risk and it may be an essential tool for providing individuals with agency over their own health outcomes. Third, our analysis did not account for the sars-cov-2 pandemic, an exogenous shock to the system, that has disrupted HIV prevention and care, and changed behaviors that impact transmission [31–35]. And finally, our model did not account for changes in behavior in response to the intervention itself. Individuals using PrEP may engage in risk compensation while others who are not on PrEP may also change their behavior and increase their risk if they underestimate their own risk based on their beliefs about their partners ART or PrEP status, ART or PrEP use in their community, and/or changes in community prevalence and incidence.

It should also be noted that our simulation only models the proximate causes of HIV transmission (e.g., sexual contact, condom use, ART status / viral load, and PrEP status). The secondary contextual and structural causes, including socioeconomic and political factors, community and neighborhood effects, healthcare access, transportation, food security, substance use, mental health, and intra-partnership violence are not explicitly modeled, and their impact is only captured in the aggregate via the parameter estimates for the proximate causes listed above. In addition, our counterfactual ART and PreP coverage levels were fixed values in order to demonstrate what could happen if such coverage levels were achieved. Achieving these ambitious levels of ART and PrEP coverage will require overcoming significant obstacles across a range of domains beyond just the clinical. As is stated in the National HIV/AIDS Strategy for the United States 2022–2025, “Inequities in the social determinants of health are significant drivers and contributors to health disparities and highlight the need to focus not only on HIV prevention and care efforts, but also on the ways that programs, practices, and policies affect communities of color and other populations that experience HIV disparities.” [9] Addressing the social determinants of health will be a necessary step in reducing HIV incidence [36, 37]. Several priority populations for HIV treatment and prevention have already been identified in the strategic plan including gay, bisexual, and other men who have sex with men, in particular Black, Latino, and American Indian/Alaska Native men; Black women; transgender women; youth aged 13–24 years; and people who inject drugs. There are ongoing programs like Targeted Highly-Effective Interventions to Reverse the HIV Epidemic (THRIVE) that focused on improving outcomes in these populations which have found some success [38–41] as well a more general approaches to facilitating entry into the prevention and treatment continuum of care like internet based HIV test distribution [42] and opt-out testing in emergency rooms and other clinical setting [43]. Our analysis indicates that it is possible to reach the EHE goal of a 75% reduction in HIV incidence by increasing ART coverage to 90% with at least 84% full viral suppression and 30% PrEP coverage, or 95% ART coverage with as little as 10% PrEP coverage. Reaching the 90% reduction in incidence is also attainable but would require 100% ART initiation with 94% full viral suppression and 50% PrEP coverage. The largest reductions in HIV incidence can be achieved by increasing ART coverage among MSM and all race groups benefit as the level of ART initiation increases regardless of any differences in ART initiation by race groups. This is consistent with finding from other studies of incidence among MSM [7]. However, improvement in ART coverage among Hispanic/Latino or White/Other MSM did not result in the level of generalized reductions in incidence among heterosexuals, particularly NHB women, which were found with improvements in ART coverage among NHB MSM. Consequently, improving ART coverage to > 90% should be prioritized with a particular emphasis on ART initiation, adherence, and retention in care among NHB MSM. Such a focus will reduce the largest number of incident cases, reduce racial HIV incidence disparities among both MSM and women, and reduce racial health disparities among persons with HIV. NHB women should also be prioritized for PrEP outreach.

## Supplementary Information

**Additional file 1:** **Appendix.**

**Additional file 2:** **Supplemental figures.**

## Data Availability

This study did not collect primary data. Data used in this study are available from their original sources https://www.cdc.gov/nchs/nsfg/index.htm and https://github.com/EpiModel/ARTnet.

## Abbreviations

HIV: Human immunodeficiency virus
NHB: Non-Hispanic Black or African American persons
EHE: Ending the HIV Epidemic in the U.S.
ART: Antiretroviral therapy
PrEP: Pre-exposure prophylaxis
MSM: Gay, bisexual, and other men who have sex with men
TasP: Treatment as prevention
MSA: Metropolitan statistical area
MSMW: Men who have sex with men and women
NSFG: National Survey of Family Growth
PWH: Persons with HIV
PIA: Percent of infections averted
NIA: The number of infections averted per 100,000 person-years at risk
SI: Simulation interval
