# Supplementary material for: Achieving the “Ending the HIV Epidemic in the U.S.” incidence reduction goals among at-risk populations in the South
Source: BMC Public Health. 2023 Apr 20;23:716. doi: 10.1186/s12889-023-15563-5 (PMC10116101; doi:10.1186/s12889-023-15563-5)
Supplement: Supplementary file 1 — Additional file 1: Appendix. [file 12889_2023_15563_MOESM1_ESM.docx]

**Achieving the “Ending the HIV Epidemic” program goals among at risk populations in the Southeastern United States**

**Technical Appendix**

#

# INTRODUCTION

We designed an agent-based stochastic network model to estimate the level of ART-based viral suppression and/or PrEP uptake that would be required to meet the EHE goal of a 90% reduction in HIV incidence among key populations in the Southeastern United States over 8 years (from 2022-2030) in order to meet the 2030 goal. Here we provide the technical details of the model.

## Model Framework

HIV transmission dynamics were modeled in this study using stochastic agent-based network microsimulations in which uniquely identifiable sexual partnership dyads were simulated and tracked over time. Each simulation included six networks representing partnership dyads of different types described below, while the node set remains the same in all six networks over time. Overlaid on top of the dynamic network simulations was a larger epidemic model that represented demography (entries, exits, and aging), interhost epidemiology (disease transmission), intrahost epidemiology (disease progression), and clinical epidemiology (disease diagnosis and treatment). Individual attributes related to these processes were stored and updated in discrete time (1-week time steps) over the course of each epidemic simulation.

## Model Software

The models in this study were programmed in the R and C++ software languages using the *statnet* R package for network model estimation and the *EpiModel* [http://epimodel.org/] software platform for epidemic modeling. *Statnet* [http://statnet.org/] is a suite of software in R for the representation, visualization, and statistical analysis of complex network data.[1] The EpiModelHIV R package, which is built on the EpiModel platform, was used to incorporate HIV-specific epidemiology. The original version of EpiModelHIV was created to model HIV transmission among men who have sex with men (MSM) in the US and South America by Goodreau et al. [2, 3] but has subsequently been expanded and modified for numerous populations and projects.[4-10] The specific version of the software package used for this study is available here https://github.com/EpiModel/EpiModelHIV-p/tree/SouthComb.

Simulations were performed on the Hyak.Mox nextgen high-performance computing (HPC) system at the University of Washington, allowing for execution of multiple simulations on large networks in parallel and reducing the overall computation time.

# EMPIRICAL DATA

The population of interest modeled in this study was 15-65 year-olds in the South. The primary data sources for this analysis were three waves of the National Survey of Family Growth (NSFG) (2011-2013 [11], 2013-2015 [12] and 2015-2017 [13]) which provided sexual network and behavioral data for the heterosexual population in our simulation, and ARTnet [14] which provided sexual network and behavioral data for the population of MSM. The NSFG is a nationally representative survey of U.S. households with independent samples of men and women. Data from 2011-2015 were limited to ages 15-44 but the survey was expanded to include ages 15-49 for 2015-17 on. Adolescents, non-Hispanic Black, and Hispanic people are oversampled. The survey includes a computer-assisted personal interview (CAPI) and audio computer-assisted self-interview (ACASI) for a subset of sensitive questions. Respondents 18 years and older provide informed consent; respondents ages 15-17 provide assent after parental permission. The surveys received ethical approval from the institutional review board at the National Center for Health Statistics. Our analysis of publicly available data is not considered human subjects research. The ARTnet study was conducted in the United States in 2017–2019 and included respondents who were male sex at birth, current male cisgender identity, lifetime history of sexual activity with another man, and age between 15 and 65. Participants were recruited directly after participating in the American Men’s Internet Study (AMIS), [15] a parent web-based study about MSM sexual health that recruited through banner ads placed on websites or social network applications. At the completion of AMIS, MSM were asked to participate in ARTnet, which focused on sexual network features. The final sample size following deduplication was n=4904 participants. The Emory University Institutional Review Board approved the study. Additional model parameters on HIV infection, prevention, and treatment were drawn from the literature and surveillance reports. When possible parameter estimates were specific to the South. These data are discussed in the appropriate sections below.

# NETWORKS OF SEXUAL PARTNERSHIPS

The NSFG collected demographic data on each of the survey respondents including their age, sex and race/ethnicity. Data were also collected on current spouse, cohabitating or other ongoing sexual partnerships, and as many as 5 of the most recent partners depending on the wave of data collection and whether the respondents were male or female. Depending on the year of the survey some of the questions about partner demographics changed over time. However, respondents did provide the age, sex, and race/ethnicity of current partners as well as the date of first sex. Current ongoing partners provide the basis of the network model as they capture the sexual network structure at the cross section. The ARTnet study collected comparable demographic and partnership data among a national convenience sample of MSM recruited online. These data informed the structure and dynamics of the relational networks described below. All relational data used in this analysis is egocentric, meaning that the participants were asked to provide information about themselves and their partners, but the partners themselves were not enrolled in the study.

For this analysis our interest was in the South. Both ARTnet and the NSFG provide sample weights to generate national parameter estimates [14] [16], but regional weights are not available for the NSFG and all geographical variables are redacted from the public use data. In order to capture much of the regional differences in the demographic composition of the population we constructed a self-weighted sample of the data by first applying the national sampling weights, which took into account the individual study sampling procedures, and then weighting the data to reflect the age by race by sex distributions reported in the South by the US census in 2018[17]. Table 1 provides the population counts for the Southern region for males and females by race group and age group from age 15 to 65. We combined the White Non-Hispanic and Other Non-Hispanic populations into a single group to provide the relative distributions within sex. We used these proportions (table 2) to generate a self-weighted sample of 500,000 individuals to inform our simulation. The sample data was generated by drawing observations with replacement from both the NSFG and ARTnet based on both the sampling weights and the demographic proportions in the South. The sample population in the ARTnet survey included MSM age 15-65, however the NSFG only included respondents up through age 44 for all three waves. In order to generate a simulated population through age 65 we sampled respondents aged 38-44 from the NSFG as proxies for the 45-65 year-old heterosexuals. Based on the census data 50.58% of the sample was drawn from the female respondents in the NSFG. The remaining 49.42% of the population was drawn from males respondents in two parts, 3.57% of the male sample was drawn from the ARTnet respondents to reflect the proportion of the male population in the South that is estimated to have same sex partners[18] and the remainder was drawn from the male respondents in the NSFG.

| **Table 1:** Population of the Southeastern United States in 2018 within sex by age and race | | | | | | | | | | | |
| --- | --- | --- | --- | --- | --- | --- | --- | --- | --- | --- | --- |
| **Males** | | | | | |  | **Females** | | | | |
|  | **Black** | **Hispanic** | **White** | **Other** | **Total** |  | **Black** | **Hispanic** | **White** | **Other** | **Total** |
| **15-18** | 712432 | 774092 | 1638029 | 271994 | 3396547 |  | 679204 | 748143 | 1550991 | 263844 | 3242182 |
| **19-24** | 1065203 | 1099537 | 2512889 | 380519 | 5058148 |  | 1080404 | 1012913 | 2350786 | 360637 | 4804740 |
| **25-34** | 1688156 | 1785666 | 4433523 | 598791 | 8506136 |  | 1819190 | 1617635 | 4382429 | 649532 | 8468786 |
| **35-44** | 1463294 | 1724822 | 4174530 | 548124 | 7910770 |  | 1685742 | 1637697 | 4184219 | 614793 | 8122451 |
| **45-54** | 1387280 | 1345598 | 4614344 | 435753 | 7782975 |  | 1613526 | 1328697 | 4675999 | 511471 | 8129693 |
| **55-65** | 1395555 | 957970 | 5465162 | 351240 | 8169927 |  | 1690399 | 1037745 | 5842409 | 436296 | 9006849 |
| **Total** | 7711920 | 7687685 | 22838477 | 2586421 | 40824503 |  | 8568465 | 7382830 | 22986833 | 2836573 | 41774701 |

| **Table 2.** U.S. population proportions in the southeast in 2018 | | | | | | | | | |
| --- | --- | --- | --- | --- | --- | --- | --- | --- | --- |
|  | **Black / AA** | **Hispanic** | **White/ other NH** | **Total** |  | **Black / AA** | **Hispanic** | **White/ other NH** | **Total** |
| **15-18** | 1.75% | 1.90% | 4.68% | 8.32% |  | 1.63% | 1.79% | 4.34% | 7.76% |
| **19-24** | 2.61% | 2.69% | 7.09% | 12.39% |  | 2.59% | 2.43% | 6.49% | 11.50% |
| **25-34** | 4.14% | 4.37% | 12.33% | 20.84% |  | 4.36% | 3.87% | 12.05% | 20.27% |
| **35-44** | 3.58% | 4.23% | 11.57% | 19.38% |  | 4.04% | 3.92% | 11.49% | 19.44% |
| **45-54** | 3.40% | 3.30% | 12.37% | 19.06% |  | 3.86% | 3.18% | 12.42% | 19.46% |
| **55-65** | 3.42% | 2.35% | 14.25% | 20.01% |  | 4.05% | 2.48% | 15.03% | 21.56% |
| **Total** | 18.90% | 18.83% | 62.28% | 100% |  | 20.51% | 17.67% | 61.82% | 100% |

From these relational data we created the network models that provided the foundation for the larger epidemic model. The methods are briefly described conceptually, followed by a technical description of the statistical modeling methods and finally a description of the estimated parameters used in the simulation.

We modeled networks of three interacting types of sexual relations: main partnerships, casual (but persistent) partnerships, and one-time sexual contacts for both the MSM population and the heterosexual population for a total of six different relational networks. A full description of the relational data from the ARTnet study is provided in Weiss et al. 2020 [14] and the code used to construct all of the parameters from the ARTnet data are available here <https://github.com/EpiModel/ARTnet>. Similarly, a full description of the NSFG is available elsewhere [19]. For the heterosexual population we matched as closely as possible the approaches used for the analysis of the MSM data and the constructions of the model parameters. For the heterosexual population, parameters constructed from data provided for partners reported in the NSFG to be either a spouse or cohabitating partner were classified as a main partnership while all other partnerships that were ongoing were classified as casual. The first three networks, which we refer to as the heterosexual networks, represent the main, casual and one-time partnerships in which penile- vaginal sex is expected to occur. The nodes, or individuals, active in these three networks are the heterosexual females, heterosexual males and the males that have both male and female partners. The proportion of MSM who have sex with both males and females was 26.4%, 12.4% and 10.9% for Black/AA, Hispanic and White/Other MSM respectively based on estimates from Dasgupta et al. 2020 [20]. Network 4 through 6, which we refer to as the MSM networks, represent the main, casual and one-time partnerships in which penile-anal sex is expected to occur between two males. The nodes, or individuals, active in these three networks are the males that have sex with exclusively males and the males that have both male and female partners.

##

## Conceptual Representation of Sexual Networks

Our modeling methods aim to preserve several of the features of the cross-sectional and dynamic network structure we observed in our primary data, while also allowing for mean relational durations to be targeted to those reported for different groups and relational types. Our methods do so within the context of changing population composition attributes such as age. The methods are robust to changes in population size as well but in this case the population size was held fixed at 500K with the number exiting the simulation either by aging out or dying replaced by new entries into the population in the subsequent time step. The broader motivation, methodological details, and link between models and primary data are described here [21].

*Relationships with duration*

We modeled two types of sexual relations with duration: main partnerships and casual (but persistent) partnerships. The ARTnet survey asked specifically about ongoing main and casual partnerships. In the NSFG partners were identified as a spouse, former spouse, cohabiting partner, former cohabiting partner, other most recent partner or other. For consistency with the MSM we considered any ongoing spouse or ongoing cohabitating relationship to be a main partnership while all other partnerships that were ongoing were classified as casual.

The network features that we aim to preserve in the main and casual networks are as follows:

- Persistent (Main and Casual) Partnerships on both the heterosexual and MSM networks
  - The mean degree (number of ongoing partners), stratified by main and casual partnership types, and the proportion of individuals with concurrency (2 or more ongoing partners) for each partnership type, at any time point.
  - Variations in the mean degree specific to each persistent partnership type by:
    - Race/ethnicity group (3 categories for Black, Hispanic, and Other).
    - Age group (6 categories for 15-18, 19–24, 25–34, 35–44, 45–54, and 55–64).
    - Age at entry (1 category indicating age 15).
    - Cross-type degree: Degree in the other persistent partnership type (e.g., mean degree of MSM for main partnerships given current casual degree of 0, 1, 2).
  - Selection of partners within the same race/ethnicity group (mixing by race/ethnicity).
  - Selection of partners within the same age group (mixing by age).
  - The average difference in age (offset on the heterosexual network to capture age asymmetry in heterosexual relationships)
  - Prohibitions against partnerships on a network representing sexual relationships in which a node does not participate
    - i.e. (males who have sex with males can’t form relationships on the heterosexual networks, females and males who have sex exclusively with females can’t form relationships on the MSM networks)
  - Prohibitions against forming partnerships with the same role exclusivity. i.e.(exclusively insertive MSM can’t form a relationship with another exclusively insertive MSM)
  - Mean partnership durations, stratified by main and casual partnership types, and by mixing within age groups.

*Relationships without duration*

We modeled networks of three interacting types of sexual relations: main partnerships, casual (but persistent) partnerships, and one-time sexual contacts for both the MSM population and the heterosexual population. One-time partnerships are those that have no duration. The ARTnet survey asked specifically about the number of one-time partnerships in the last year. The NSFG did not specifically ask about one-time partnerships so we estimated the number of one-time partnerships in the last year to be the total number of opposite sex sexual partnerships reported in the last year minus any reported ongoing partnerships. Because the NSFG also did not ask about the demographic details of all partners in the last year the age and race/ethnicity were imputed based on the age and race/ethnicity of the respondent and the age and race/ethnicity distribution of the main and casual partners.

The network features that we aim to preserve in the one-time partnership networks are as follows:

- One-Time Partnerships
  - The overall rate of having one-time intercourse (anal for MSM and vaginal for heterosexuals) partnerships per week.
  - Variations in this contact rate by:
    - Race/ethnicity group.
    - Age group.
    - Total persistent degree (sum of main and casual partnerships ongoing).
    - Risk level heterogeneity above variations by these three factors (mean partnership rates for five quintiles of MSM and heterosexuals stratified by mean one-time rates).
  - Selection of partners within the same race/ethnicity group (mixing by race/ethnicity).
  - Selection of partners within the same age group (mixing by age).
  - Prohibitions against partnerships on a network representing sexual relationships in which a node does not participate
    - i.e.(males who have sex with males can’t form relationships on the heterosexual networks, females and males who have sex exclusively with females can’t form relationships on the MSM networks)
  - Prohibitions against forming partnerships with the same role exclusivity. i.e.(exclusively insertive MSM can’t form a relationship with another exclusively insertive MSM)

*Network Model Parameters*

Here we describe each of the parameters used in the network models.

*Mean Degree / Number of Ongoing Partnerships (Main / Casual)*

Ongoing partnerships (whether main or casual) were defined from the egocentrically reported relationship data as those relationships in which penile-vaginal intercourse for heterosexuals or penile-anal intercourse had already occurred and for which the respondent reported that they were likely to have sexual intercourse again.

The momentary main or casual mean degree is then defined as the mean of the degree of all individuals in the population for main or casual heterosexual or MSM partnerships on the day of study. We estimated this with a Poisson model with main or casual heterosexual or MSM degree as the outcome and then exponentiating the coefficients, resulting in an estimated mean main degree of .5077 in the main heterosexual network, .2036 on the casual heterosexual network, .0065 on the main MSM network and 0.0102 on the casual MSM network. Note that the mean degree is far smaller on the MSM networks because the node set that represents the entire population is consistent across all six networks, it is the relationship types that change. Thus, the mean degree for main MSM partnerships is the number of main MSM partnerships divided across the entire population, not just the MSM. In addition, we modeled the proportion of individuals with concurrency (degree of 2 or more) by partnership type on each of the 4 networks. This was estimated with logistic regression models for binary outcomes. Taking the inverse of the logit of the coefficient yielded the predicted probabilities of 8.8e^-5^ and .011% for main and casual concurrency on the respective heterosexual networks and .0003% on both the main and casual MSM networks. Again note that the values on the MSM network are based on the entire population of 500K nodes while the MSM are less than 8K nodes.

We estimated the heterogeneity in main and casual mean degree by fitting three Poisson regression models. For race/ethnicity, we estimated the mean degree for each group within the target population by including dummy variables for race/ethnicity. For age, we modeled the semi-parametric relationship between age and mean degrees by including age group and square root of age group to allow for a non-linear relationship between age and the outcome. For nodes age 15, we modeled the mean degree for main and casual partnerships with a binary predictor for nodes being age. For cross type degree, we modeled the mean degree for main partnerships as a function of degree of casual partnerships, and vice versa. For each of the 16 models (4 partnership types times four predictors of interest), we estimated the statistical models and then exponentiated the coefficients to obtain the rates for each stratum. Those are shown in the Table below.

| **Table 3.** Race/Ethnicity, Age, and Cross-partnership type heterogeneity in Main and Casual partnership degree | | | | |
| --- | --- | --- | --- | --- |
|  | **Main heterosexual** | **Casual Heterosexual** | **Main MSM** | **Casual MSM** |
| **Race / Ethnicity** |  |  |  |  |
| Black | .357 | .360 | .0042 | .0103 |
| Hispanic | .511 | .173 | .0064 | .0103 |
| Other | .557 | .162 | .0072 | .0102 |
| **Age Groups** |  |  |  |  |
| 15-18 | .040 | .241 | .0034 | .0031 |
| 19-24 | .222 | .261 | .0066 | .0061 |
| 25-34 | .495 | .243 | .0080 | .0090 |
| 35-44 | .686 | .209 | .0078 | .0113 |
| 45-54 | .702 | .171 | .0065 | .0130 |
| 55-65 | .578 | .136 | .0049 | .0139 |
| **Age at Entry**  **Age 15** |  |  |  |  |
| No | .519 | .207 | .0066 | .0105 |
| Yes | .002 | .067 | .0010 | .0008 |
| **Cross partnership type** |  |  |  |  |
| 0 | .622 | .405 | .0048 | .0077 |
| 1+ | .021 | .009 | .2746 | .4206 |

*Race and Age Mixing*

Respondents in both the ARTnet study and the NSFG reported on their perception of the race and ethnicity (Hispanic/non-Hispanic) for each of their ongoing partners. We recoded the partner race reported in both studies into three mutually exclusive groups: Black, Hispanic, and Other. Using logistic regression models, we estimated the proportion of partnerships that were between individuals of the same race (within-group mixing) by the race of the respondent race by evaluating relationships that matched on race between the respondent group and partner group as a binary outcome. The inverse logit of the coefficients is then interpreted as the predicted probability of a same-race/ethnicity partnership for each race group. The values are shown in the table below

| **Table 4.** Race/Ethnicity homophily by race and partnership type | | | |
| --- | --- | --- | --- |
|  | **Black** | **Hispanic** | **Other** |
| **Main Heterosexual** | 83.3% | 79.1% | 92.4% |
| **Casual Heterosexual** | 82.0% | 67.1% | 82.8% |
| **Main MSM** | 54.8% | 39.9% | 81.2% |
| **Casual MSM** | 50.3% | 35.2% | 72.3% |

For mixing by age, we used a model parameterization for the 6-category age group that allowed for differences in the level of age mixing that could vary by age group (differential homophily). We fit a logistic regression model for partnerships, with being in a partnership of the same age group as the outcome and the age group of the respondent as the main predictor. With the inverse logit transformation, the probabilities of partnerships within the same age group, stratified by partnership type are shown in the table below.

| **Table 5.** Age mixing by partnership type | | | | |
| --- | --- | --- | --- | --- |
| **Age** | **Main Heterosexual** | **Casual Heterosexual** | **Main MSM** | **Casual MSM** |
| **15-18** | 52.5% | 67.2% | 65.5% | 52.4% |
| **19-24** | 57.3% | 65.6% | 59.5% | 45.4% |
| **25-34** | 61.9% | 64.0% | 53.2% | 38.6% |
| **35-44** | 66.3% | 62.4% | 46.8% | 32.2% |
| **45-54** | 70.4% | 61.7% | 40.5% | 26.5% |
| **55-65** | 74.2% | 59.0% | 34.5% | 21.4% |

*Partnership Durations and dissolutions*

We model partnership dissolution as a heterogenous, geometrically distributed process with unique parameters for each relational type. The geometric distribution for relational durations implies a “memoryless process.” Although this assumption implies that the rate of dissolution does not depend on the current age of the partnership, the overall exponential shape of the dissolution distribution matches reasonably well to empirical data on relational durations. Following Jenness et al. [8] we stratified by partnership types and the interaction between partnership type and age of both of the members within the dyad, estimating specific distributions based on matched age groups (that is, partnerships between two persons of the same age). Raw relational ages were calculated as the difference between date of first sex and the date of interview for each ongoing relationship. To derive our estimator of relational age, we take the median of the observed distribution and then calculate the mean for the geometric distributions associated with that median. The resulting expected relational ages are summarized in the table below.

| **Table 6.** Duration of main and casual heterosexual and MSM partnerships by dyadic age group of the respondent and their partner | | | | |
| --- | --- | --- | --- | --- |
| **Age group** | **Main Heterosexual Relational Age (weeks)** | **Casual Heterosexual Relational Age (weeks)** | **Main MSM Relational Age (weeks)** | **Casual MSM Relational Age (weeks)** |
| **Both 15-18** | 119.3 | 31.8 | 33.3 | 29.6 |
| **Both 19-24** | 206.8 | 81.8 | 105.2 | 84.6 |
| **Both 25-34** | 500.6 | 113.0 | 242.8 | 71.3 |
| **Both 35-44** | 1063.3 | 144.3 | 420.9 | 134.5 |
| **Both 45-54** | 1144.6 | 131.8 | 631.9 | 136.3 |
| **Both 55-65** | 1175.8 | 138.0 | 807.0 | 168.5 |
| **Different groups** | 725.7 | 100.5 | 194.9 | 112.0 |

*Onetime partnerships*

We modeled one-time sexual contacts involving anal intercourse between two males based on ARTnet reports on the number and variation in these types of relations. We calculated a weekly rate of new contacts by subtracting the total main and casual partners from the total past-year partners. We similarly calculated a weekly rate of new contacts involving vaginal intercourse between males and females based on NSFG reports on the number of total vaginal sex partnerships reported in the last year minus the current ongoing partnerships. Demographic information was not provided for one-time partnerships so we imputed the demographic attributes of one-time partners based on the demographic distributions of main and casual partnerships conditional on the demographic attributes of the respondent. We estimated the weekly rate by fitting a Poisson regression model with the count of one-time contacts, exponentiating the coefficient to get the predicted count, and dividing by 52 to get the week rate. The overall mean one-time contact rate was 0.006 vaginal sex contacts per week and 0.080 AI contacts per week.

Heterogeneity in one-time contact rates was modeled with three Poisson regression models to estimate the rates as a function of race/ethnicity, age group and risk level strata. Similar to the one-time rate, we fit these models and exponentiated the coefficients and then divided by 52 to get the group-specific rates. For age group, similar to the estimation of degree, we modeled this semi-parametrically by including age group and the square root of age group as the joint predictors. The results are shown in the table below.

| **Table 7.** Race/Ethnicity, Age and Risk level heterogeneity in One-Time Partnerships | | |
| --- | --- | --- |
|  | **One-Time Heterosexual** | **One-Time MSM** |
| **Race / Ethnicity** |  |  |
| Black | .009 | .058 |
| Hispanic | .006 | .090 |
| Other | .006 | .086 |
| **Age Groups** |  |  |
| 15-18 | .009 | .029 |
| 19-24 | .010 | .059 |
| 25-34 | .008 | .083 |
| 35-44 | .006 | .095 |
| 45-54 | .005 | .097 |
| 55-65 | .003 | .091 |
| **Risk Level Quintile** |  |  |
| 1 | 5.6e-10 | 4.14e-9 |
| 2 | 5.6e-10 | 4.14e-9 |
| 3 | 5.6e-10 | .0118 |
| 4 | 5.6e-10 | .0450 |
| 5 | .03 | .345 |

*Mixing by Race and Age in One-Time Partnerships*

We used the same approach to within-group mixing by race/ethnicity and age group for one-time contacts that was used for persistent contacts. The proportions for each race / ethnicity and age group are shown in the table below:

| **Table 8.** Race and age group homophily in One-Time Partnerships | | |
| --- | --- | --- |
|  | **One-Time Heterosexual** | **One-Time MSM** |
| **Race / Ethnicity** |  |  |
| **Black** | 81.7% | 39.8% |
| **Hispanic** | 76.8% | 30.7% |
| **Other** | 90.0% | 71.7% |
| **Age Group** |  |  |
| **15-18** | 79.5% | 48.6% |
| **19-24** | 74.6% | 43.0% |
| **25-34** | 69.0% | 37.5% |
| **35-44** | 62.8% | 32.3% |
| **45-54** | 56.1% | 27.5% |
| **55-65** | 49.2% | 23.1% |

*Mixing by Sexual Role*

We assign the MSM in our simulation a fixed sexual role preference (exclusively insertive, exclusively receptive, versatile). The model then includes a prohibition against two exclusively insertive or two exclusively receptive men forming a partnership on an MSM network. We estimated the proportion of men that were in each category (insertive, receptive, and versatile) by analyzing whether men had only insertive anal intercourse, only receptive anal intercourse, or both insertive and receptive anal intercourse (respectively) in their past five anal partnerships over the past year. The proportions were: 21.8% exclusively insertive, 26.5% exclusively receptive, and 51.7% versatile. Similar prohibitions also prevent men who exclusively have sex with other men from forming relationships on any of the heterosexual networks, same sex partnerships of any kind from forming on any of the heterosexual networks, or females from forming partnerships on any of the MSM networks.

## Statistical Representation of Sexual Networks

Exponential-family random graph models (ERGMs) and their dynamic extension temporal ERGMs (TERGMs) provide a foundation for statistically principled simulation of local and global network structure given a set of target statistics from empirical data. Main and casual relationships were modeled using TERGMs,[22] since they persist for multiple time steps. One-time contacts, on the other hand, were modeled using cross-sectional ERGMs.[23] Formally, our statistical models for relational dynamics can be represented as 10 equations for the conditional log odds (logits) of relational formation and persistence at time *t* (for heterosexual and MSM main and casual relationships) or for relational existence at time *t* (for heterosexual or MSM one-time contacts):

$logit\left( P\left( Y_{ij,t}=1 | Y_{ij,t-1}=0\text{, }Y_{ij,t}^{C} \right) \right)\text{ = }{\theta_{mh}^{+}}^{'}\partial\left( g_{mh}^{+}\left( y \right) \right)$ Main heterosexual partnership formation

$logit\left( P\left( Y_{ij,t}=1 | Y_{ij,t-1}=0\text{, }Y_{ij,t}^{C} \right) \right)\text{ = }{\theta_{ch}^{+}}^{'}\partial\left( g_{ch}^{+}\left( y \right) \right)$ Casual heterosexual partnership formation

$logit\left( P\left( Y_{ij,t}=1 | Y_{ij,t-1}=0\text{, }Y_{ij,t}^{C} \right) \right)\text{ = }{\theta_{mm}^{+}}^{'}\partial\left( g_{mm}^{+}\left( y \right) \right)$ Main MSM partnership formation

$logit\left( P\left( Y_{ij,t}=1 | Y_{ij,t-1}=0\text{, }Y_{ij,t}^{C} \right) \right)\text{ = }{\theta_{cm}^{+}}^{'}\partial\left( g_{cm}^{+}\left( y \right) \right)$ Casual MSM partnership formation

$logit\left( P\left( Y_{ij,t}=1 | Y_{ij,t-1}=1\text{, }Y_{ij,t}^{C} \right) \right)\text{ = }{\theta_{mh}^{-}}^{'}\partial\left( g_{mh}^{-}\left( y \right) \right)$ Main heterosexual partnership persistence

$logit\left( P\left( Y_{ij,t}=1 | Y_{ij,t-1}=1\text{, }Y_{ij,t}^{C} \right) \right)\text{ = }{\theta_{ch}^{-}}^{'}\partial\left( g_{ch}^{-}\left( y \right) \right)$ Casual heterosexual partnership persistence

$logit\left( P\left( Y_{ij,t}=1 | Y_{ij,t-1}=1\text{, }Y_{ij,t}^{C} \right) \right)\text{ = }{\theta_{mm}^{-}}^{'}\partial\left( g_{mm}^{-}\left( y \right) \right)$ Main MSM partnership persistence

$logit\left( P\left( Y_{ij,t}=1 | Y_{ij,t-1}=1\text{, }Y_{ij,t}^{C} \right) \right)\text{ = }{\theta_{cm}^{-}}^{'}\partial\left( g_{cm}^{-}\left( y \right) \right)$ Casual MSM partnership persistence

$logit\left( P\left( Y_{ij,t}=1 | Y_{ij,t}^{C} \right) \right)\text{ = }{\theta_{oh}}^{'}\partial\left( g_{oh}\left( y \right) \right)$ One-time heterosexual contact existence

$logit\left( P\left( Y_{ij,t}=1 | Y_{ij,t}^{C} \right) \right)\text{ = }{\theta_{oh}}^{'}\partial\left( g_{oh}\left( y \right) \right)$ One-time MSM contact existence

where:

- $Y_{ij,t}$ = the relational status of persons *i* and *j* at time *t* (1 = in relationship/contact, 0 = not)
- $Y_{ij,t}^{C}$ = the network complement of *i,j* at time *t*, i.e. all relations in the network other than *i,j*
- $g\left( y \right)$ = vector of network statistics in each model
- $\theta$ = vector of parameters in the formation model

For $g\left( y \right)$ and $\theta$, the superscript distinguishes the formation model (+), persistence model (-) and existence models (neither). The subscript indicates the main heterosexual (mh), casual heterosexual (ch), main MSM (mm), casual MSM (cm), one-time heterosexual (oh), and one-time MSM (om) models. The recursive dependence among the relationships renders the model impossible to evaluate using standard techniques; we used Markov chain Monte Carlo (MCMC) methods in order to obtain the maximum likelihood estimates for the $\boldsymbol{\theta}$ vectors given the $\boldsymbol{g}\left( \boldsymbol{y} \right)$ vectors.

Our method of converting the statistics laid out above into our fully specified network models consists of the following steps:

1. Create a self-weighted sample of 500,000 respondents from the NSFG and ARTnet survey data and the demographic distributions reported for the Southeastern region by the census.
2. Create an empty network of 500,000 nodes and assign each node the attributes from an observation from the self-weighted sample.
3. Calculate the target statistics (i.e., the expected count of each statistic at any given moment in time) associated with the terms in the formation model (for the main and casual heterosexual and MSM partnerships) and in the existence model (for one-time heterosexual and MSM contacts).
4. For the main and casual networks, use the mean relational durations by age group combination to calculate the parameters of the persistence model, using closed-form solutions, given that the models are dyadic-independent (each relationship’s persistence probability is independent of all others).
5. For the main and casual networks, estimate the coefficients for the formation model that represent the maximum likelihood estimates for the expected cross-sectional network structure.
6. For the one-off network, estimate the coefficients for the existence model that represent the maximum likelihood estimates for the expected cross-sectional network structure.

Steps 4–6 occur within the EpiModel software, and use the ERGM and STERGM methods therein. They are completed efficiently by the use of an approximation in Step 5. [24] During the subsequent model simulation, we use the method of Krivitsky [25] to adjust the coefficient for the first term in each model at each time step, in order to preserve the same expected mean degree (relationships per person) over time in the face of changing nodal composition. At all stages of the project, simulated partnership networks were checked to ensure that they retained the expected cross-sectional structure and relational durations throughout the simulations.

# BEHAVIOR WITHIN SEXUAL PARTNERSHIPS

We modeled three phenomena consecutively within relationships at each time step: the number of sex acts (penile-vaginal on the heterosexual networks and penile-anal on the MSM networks), condom use per sex act on all networks, and role per sex act on the MSM networks.

## Frequency of intercourse

Coital frequency for the main and casual heterosexual and MSM partnerships were calculated from the NSFG and ARTnet survey data respectively. In the NSFG respondents were asked how often in the last four weeks they had had sex. The question was a summary question that was not specific to a particular partnership. Therefore, we limited our analysis to respondents with just one partner, either a main or casual partnership, and only included those with partnerships that had been ongoing for at least four weeks to avoid underestimating coital frequency. We calculated the mean number of acts over the four weeks and divided by four to get the expected weekly count. In the ARTnet study participants were asked about the frequency of AI acts within partnerships. Participants could report on the average number of acts within the partnership over the past year by week, month, year, or total partnership duration. These responses were scaled into total weekly acts. Using the partnerships as the unit of analysis we were then able to model the counts of acts per year using Poisson regression. Acts rates are modeled based on the expectation that changes in coital frequency depend on the duration of the partnership, race/ethnicity, partnership type, age and whether or not a member of the partnership is an adolescent.

Poisson regression formula:

Y_i_ ~ b_0_ + b_1_X_1_ + b_2_X_1_^2^+ b_3_X_2_ + b_4_X_3_ + b_5_X_1_X_3_ + b_6_X_4_ + b_7_X_4_^2^ + b_8_X_5_

where:

Y_i_ = Log of acts per year.

X_1_ = Duration of partnership in weeks at the survey date.

X_2_ = Racial/ethnic combination of the ego (respondent) and alter (partner), coded in 6 categories to capture within and across group mixing: black-black, black-Hispanic/white, Hispanic-black/ white, Hispanic-Hispanic, white-black/Hispanic, white-white.

X_3_ = Partnership type (main or casual).

X_4_ = The combined age of ego and alter in years.

X_5_ = A dummy variable indicating if either the respondent or the alter is an adolescent under the age of 18

| **Table 9.** Statistical Model of Act Rate in Main and Casual Heterosexual and MSM Partnerships | | | | | | |
| --- | --- | --- | --- | --- | --- | --- |
|  | **Heterosexual Penile-Vaginal Sex** | | | **MSM Anal Sex** | | |
| **Model Parameter** | **Estimate** | **Lower 95% CI** | **Upper 95% CI** | **Estimate** | **Lower 95% CI** | **Upper 95% CI** |
| **b_0_ (Intercept)** | 4.93e+00  -1.309e-03 | 4.92e+00 | 4.93e+00 | 4.242e+00  -1.469e-03 | 4.21e+00 | 4.27e+00 |
| **b_1_ (Duration)** | -1.31e-03 | -1.31e-03 | -1.31e-03 | -1.469e-03 | -1.50e-03 | -1.44e-03 |
| **b_2_ (Duration^2^)** | 7.70e-07 | 7.67e-07 | 7.73e-07 | 7.45e-07 | 7.26e-07 | 7.64e-07 |
| **b_3_ (B-H/O Combo)** | 7.28e-02 | 7.05e-02 | 7.51e-02 | 5.05e-01 | 4.92e-01 | 5.17e-01 |
| **b_3_ (H-B/O Combo)** | 6.93e-02 | 6.73e-02 | 7.12e-02 | 3.38e-02 | 2.16e-02 | 4.60e-02 |
| **b_3_ (H-H Combo)** | 8.45e-02 | 8.31e-02 | 8.59e-02 | 1.20e-01 | 1.07e-01 | 1.33e-01 |
| **b_3_ (O-B/H Combo)** | 1.84e-01 | 1.83e-01 | 1.86e-01 | 2.91e-01 | 2.80e-01 | 3.02e-01 |
| **b_3_ (O-O Combo)** | -4.00e-02 | -4.12e-02 | -3.89e-02 | 7.20e-02 | 6.17e-02 | 8.23e-02 |
| **b_4_ (Casual Type)** | -3.06e-01 | -3.07e-01 | -3.05e-01 | -1.21e+00 | -1.22e+00 | -1.20e+00 |
| **b_5_ (Duration x Casual type)** | -6.25e-05 | -6.64e-05 | -5.86e-05 | 9.69e-03 | 8.91e-03 | 1.05e-02 |
| **b_6_ (Combined Age)** | -6.47e-04 | -7.50e-04 | -5.43e-04 | -5.71e-05 | -6.21e-05 | -5.21e-05 |
| **b_7_ (Combined Age^2^)** | -8.40e-06 | -8.99e-06 | -7.81e-06 | 2.16e-01 | 2.01e-01 | 2.31e-01 |
| **b_8_ (Adolescent < Age 18)** | -5.28e-01 | -5.32e-01 | -5.24e-01 | -3.80e-04 | -4.13e-04 | -3.47e-04 |

Predicted weekly rates of penile-vaginal sex among heterosexual partnerships and anal sex among MSM were based on the linear combination of partnership and individual attributes in the equation above. In the simulation these rates were obtained dynamically by predicting from the statistical model with inputs based on the current state of partnerships in the simulated population. EpiModel tracks the age of partners, the duration of their partnership, their racial combination, the partnership type, and an indicator for an adolescent less than age 18 in the partnership. The predict function in R was used to obtain the weekly mean rates in each strata.

Neither the NSFG nor ARTnet surveys provide data to indicate changes in the frequency of coital acts following the onset of late-stage AIDS. However, other studies have suggested that late-stage AIDS is associated with a decline in sexual activity.[26, 27] In our simulations we assume that coital frequency drops to zero once HIV viral load reaches 5.75.

## Condom use

We modeled condom use within all three partnership types (main, casual, and one-time contacts) for the heterosexual and MSM partnerships based on the NSFG and ARTnet data on the frequency of condom use within reported partnerships. The NSFG provided reported condom use over the last four weeks and ARTnet provided reported condom use frequency within partnerships as was the case for coital frequency. From the data we calculated the proportion of acts that included condom use. This resulted in a U-shaped distribution of proportions, with most persistent partnerships involving either always or never condom use. The distributions of condom use are shown in figure 1 below. We therefore, simplified the outcome variable to any condom use (yes, no). We then followed the same general approach to measuring, fitting statistical models, and dynamically predicting condom use within EpiModel as we used for coital frequency.

Figure 1. Condom use within heterosexual and MSM partnerships


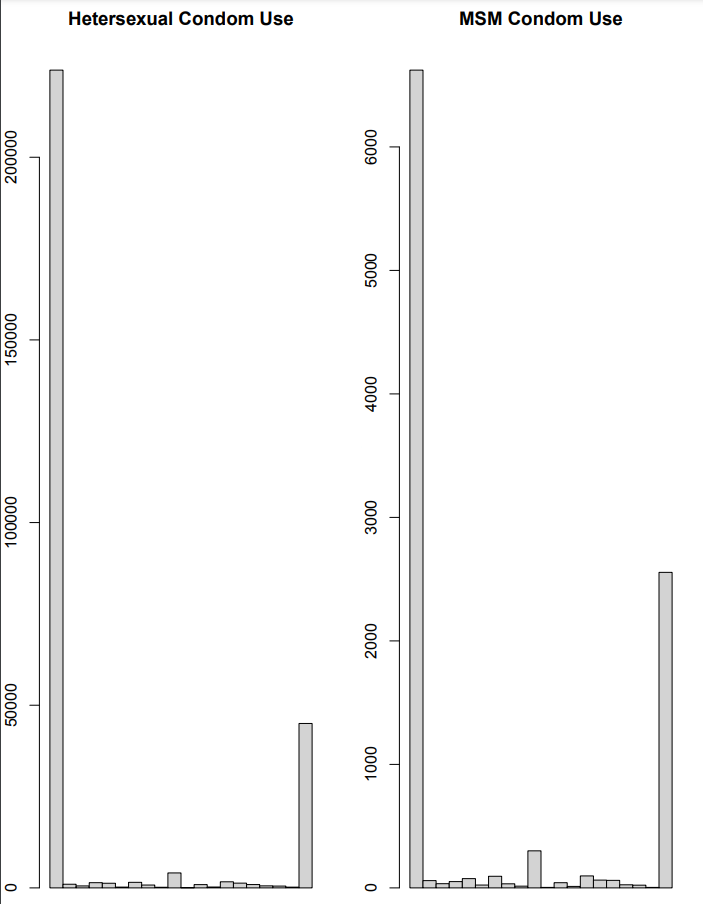


#

Logistic regression model:

*Yi ~* b*_0_ +* b*_1_X_1_ +* b*_2_X_1_^2^ +* b*_3_ X_2_ +* b*_4_X_3_ +* b*_5_X_1_X_3_ +* b*_6_X_4_ +* b*_7_X_4_^2^ +* b*_8_ X_5_*

where:

*Y_i_ =* Log odds of the probability of condom use per act.

*X_1_* = Duration of partnership in weeks at the survey date.

*X_2_ =* Racial/ethnic combination of the ego (respondent) and alter (partner), coded in 6 categories to capture within and across group mixing: black-black, black-Hispanic/white, Hispanicblack/ white, Hispanic-Hispanic, white-black/Hispanic, white-white.

*X_3_ =* Partnership type (main or casual).

*X_4_ =* The combined age of ego and alter in years.

*X_5_ =* An adolescent under age 18 in the partnerships

| **Table 10.** Statistical Model of Per Act Condom Use Probability for Main and Casual Heterosexual and MSM Partnerships | | | | | | |
| --- | --- | --- | --- | --- | --- | --- |
|  | **Heterosexual** | | | **MSM** | | |
|  | **Estimate** | **Lower 95% CI** | **Upper 95% CI** | **Estimate** | **Lower 95% CI** | **Upper 95% CI** |
| **b_0_ (Intercept)** | 8.64e-01 | 7.64e-01 | 9.65e-01 | 2.59e+00 | 2.09e+00 | 3.10e+00 |
| **b_1_ (Duration)** | 8.74e-05 | -1.85e-05 | 1.94e-04 | -2.89e-03 | -3.52e-03 | -2.26e-03 |
| **b_2_ (Duration^2^)** | -8.57e-08 | -1.63e-07 | -8.55e-09 | 1.04e-06 | 5.31e-07 | 1.51e-06 |
| **b_3_ (B-H/O Combo)** | 1.69e-01 | 1.15e-01 | 2.24e-01 | -2.91e-01 | -4.92e-01 | -9.02e-02 |
| **b_3_ (H-B/O Combo)** | -1.94e-01 | -2.44e-01 | -1.45e-01 | -4.66e-01 | -6.47e-01 | -2.85e-01 |
| **b_3_ (H-H Combo)** | 2.11e-01 | 1.77e-01 | 2.45e-01 | -4.73e-01 | -6.73e-01 | -2.73e-01 |
| **b_3_ (O-B/H Combo)** | -1.65e-01 | -2.11e-01 | -1.20e-01 | -4.60e-01 | -6.30e-01 | -2.89e-01 |
| **b_3_ (O-O Combo)** | 3.11e-02 | 3.59e-03 | 5.87e-02 | -6.15e-01 | -7.66e-01 | -4.63e-01 |
| **b_4_ (Casual Type)** | 1.29e+00 | 1.26e+00 | 1.32e+00 | 4.37e-01 | 3.12e-01 | 5.62e-01 |
| **b_5_ (Duration x Casual type)** | -6.20e-04 | -7.05e-04 | -5.34e-04 | 1.01e-03 | 5.17e-04 | 1.51e-03 |
| **b_6_ (Combined Age)** | -5.35e-02 | -5.60e-02 | -5.10e-02 | -6.48e-02 | -7.80e-02 | -5.16e-02 |
| **b_7_ (Combined Age^2^)** | 2.43e-04 | 2.29e-04 | 2.58e-04 | 3.40e-04 | 2.55e-04 | 4.24e-04 |
| **b_8_ (Adolescent < Age 18)** | 1.38e-01 | 6.43e-02 | 2.13e-01 | 1.74e-01 | -8.77e-02 | 4.39e-01 |

Predicted condom use in heterosexual and MSM main and casual partnerships were based on the linear combination of partnership and individual attributes in the equation above. In the simulation the rbinom R function was used to draw for a binomial distribution based on the above model generating either a 0 or 1 for each act to indicate in 0-no condom was used or 1 – a condom was used. EpiModel tracks the age of partners, the duration of their partnership, their racial combination, the partnership type, and an indicator for an adolescent less than age 18 in the partnership.

*Sexual Role*

We assigned each MSM a sexual role preference upon entry into the simulated population as described above. Following Jenness et al. versatile MSM were each assigned a preference for being an insertive partner from a draw from a uniform distribution between 0 and 1. When two versatile men were partnered a man’s probability of being the insertive partner equals his preference divided by the sum of the two men’s preference. Being the incentive partner was then determined based on a draw for a binomial distribution. The draw from the binomial distributions was done for each act. Exclusively insertive MSM are prevented from partnering via the TERGM models as are two exclusively receptive MSM. No sexual role was assigned for heterosexuals as all sex acts are penile-vaginal by default.

# DEMOGRAPHY

The composition of the population of interest in our simulation is 15- through 65-year-old males and females in the South. The age, sex and race/ethnicity composition of the simulated population was drawn for the 2018 census. In this model, there were three demographic processes: entries, exits, and aging.

## Entry

All persons entered the network at age 15. The number of new entries at each time step was equivalent to the number of individuals that exited the simulation in the prior time step in order to preserve the overall simulated population size N=500,000.

## Initialization of Attributes

The initial conditions in our simulation are drawn directly from our data. The self-weighted sample of 500K observations matches 1:1 to the simulated population thus each node takes on all of the attributes (age, race, sex, sexual preference) of a specific observation in the data. Persons entering the population were assigned attributes, some of which remained fixed (e.g., race, sex, sexual preference, circumcision) while others varied over time (e.g., age, relationship status and disease status). Here we describe the attributes in the first category.

All new entries into the simulation enter at age 15. Upon entry into the simulated population individual are assigned one of nine demographic categories based on the combination of race / ethnicity, sex and sexual preference. The categories are (Black-Male-MSM, Hispanic-Male-MSM, Other-Male-MSM, Black-Male-Heterosexual, Hispanic-Male-Heterosexual, Other-Male-Heterosexual, Black-Female-Heterosexual, Hispanic-Female-Heterosexual, Other-Female-Heterosexual. The probability of being assigned to any of the above nine categories was based on their relative frequencies in the self-weighted sample of 500K observations from the NSFG and ARTnet described above. The probability distribution for the nine demographic categories is (0.0032, 0.0035, 0.0109, 0.0909, 0.1033, 0.2823, 0.1055, 0.1028, 0.2975). Because the draws are stochastic the composition of the populations in each simulation is unique, but matches the observed on average. Once the demographic categories are assigned for all nodes entering the population the MSM are assigned to either exclusively have sex with other males or to have sex with both males and females. Based on estimates from Dasgupta et al. 2020 26.4% of Black MSM, 12.11% of Hispanic and 10.9% of Other MSM were assigned to have sex with both males and females.

Circumcision status was randomly assigned to incoming males based on their assigned race with Black, Hispanic and Other being assigned circumcision with probability 0.76, 0.44, and 0.91 respectively.[28] Circumcision was associated with a 60% reduction in the per-act probability of infection among HIV- males for insertive anal or vaginal intercourse only (i.e., circumcision did not lower the transmission probability if the HIV+ partner was insertive). [29]

## Exits from the Network

The population of interest in this model was individual age 15-65. Persons exited the network either from mortality or reaching the upper age bound of the target population of interest. Mortality included both natural (non-HIV-related) and HIV-related mortality causes before age 65. Background non-HIV-related mortality rates were based on reported age- race- and sex-specific mortality reported in the United States Life Tables, 2012 [30] which provided 300 unique mortality probabilities. These annual probabilities were converted to weekly probabilities and applied to persons within the population at each time step stochastically by drawing from a binomial distribution for each eligible person with a probability parameter corresponding to that person’s risk of death tied to their age, sex and race. HIV-related mortality was modeled based on clinical HIV disease progression, as described later. For nodes that reached the maximum age of 65 exit was modeled deterministically (probability = 1).

## Aging

The aging process in the population was linear by time step for all active persons. The unit of time for each step in these simulations was one week, and therefore, persons were aged in weekly steps between the minimum and maximum ages allowed (15 and 65 years old). Evolving age impacted background mortality, age-based mixing in forming new partnerships, and other behavioral features of the epidemic model. Persons who exited the network were no longer active and their attributes such as age were no longer updated.

# INTRAHOST EPIDEMIOLOGY

Intrahost epidemiology included the model features and parameters that govern the natural progression of disease within HIV-positive persons in the absence of clinical intervention. The main component of progression that was explicitly modeled for this study was HIV viral load, which controlled both interhost epidemiology (HIV transmission rates) and disease progression, eventually leading to mortality.

Following prior approaches[2, 3, 6, 9, 31], we modeled changes in HIV viral load to account for the high level of viremia during acute-stage infection, viral set point during the long chronic stage of infection, and the subsequent rise in viral load at clinical AIDS and disease-related mortality. A starting viral load of 0 was assigned to all persons upon infection. From there, the natural viral load curve was fit with the parameters shown in table 11. The HIV viral load had a crucial impact on the rates of HIV transmission within serodiscordant couples in the model, and this interaction is detailed below.

| Table 11. Intrarhost epidemiology parameters | | |
| --- | --- | --- |
| **Parameter** | **Value** | **Reference** |
| Time to peak viremia in acute stage | 12 days | Robb [32] |
| Level of peak viremia | 6.76 log_10_ | Robb [32] |
| Time from peak viremia to viral set point | 20 days | Robb [32] |
| Level of viral set point | 4.0 log_10_ | Robb [32] |
| Duration of chronic stage infection (no ART) | 3550 days | Buchbinder[33] Katz[34] |
| Duration of AIDS stage | 728 days | Buchbinder[33] |
| Level of fatal viral load | 7.0 log_10_ | Estimated from the duration of each stage |

Following infection, it took 12 days to reach peak viremia, at a level of 6.76 log 10.[32] From peak viremia, it took another 20 days to reach viral set point (4.0 log 10).[32] The total time of acute stage infection was therefore 32 days. The duration of chronic stage infection in the absence of clinical intervention was 3550 days, or approximately 10 years.[33] The AIDS stage lasted for 728 days in the absence of intervention.[33] Viral loads increased over time to a viral load of 7.0 log10, at which point it was fatal. This viral load trajectory was for antiretroviral therapy (ART)-naïve persons only, and the influence of ART on disease progression is detailed in the ART section. Transitions were deterministic for all ART-naïve persons. During the AIDS stage, HIV-related mortality was imposed stochastically with a constant risk of 1/104, corresponding to average duration of the AIDS stage in weeks. HIV-related mortality during the AIDS stage was drawn from a binomial (Bernoulli) distribution for all eligible individuals in the AIDS stage.

# CLINICAL EPIDEMIOLOGY

Clinical epidemiological processes refer to the steps along the HIV care continuum after initial infection: diagnosis, linkage to care, treatment initiation and adherence, and HIV viral load suppression. In this model, these clinical features had critical interactions with behavioral features detailed above, as well as impacts on the rates of HIV transmission, detailed below. The features of our model’s clinical processes generally followed the steps of the HIV care continuum, in which persons transitioned across states from infection to diagnosis to HIV care linkage and ART initiation to HIV viral suppression. HIV-positive individuals could also discontinue ART and re-initiate at a later time point. HIV-negative individuals could also initiate, discontinue and re-initiate PrEP. The clinical epidemiology features of the model were modified to simulate counterfactual levels of both ART coverage and PrEP use to estimate how changes in these aspects of the prevention and care continua could be leveraged to achieve the EHE goals of a 95% reduction in HIV incidence.

## HIV TESTING

In this simulation undiagnosed individuals were exposed to regular diagnostic HIV-testing which served as the entry point for both HIV prevention and treatment services in the baseline model. Both the NSFG and ARTnet studies asked respondents about the date of their last HIV- test which provided an approximate estimate of testing frequency assuming that testing is a memoryless process following a geometric distribution. However, our approach here was to use these values as starting parameters and to calibrate our model to fit diagnosis rates as reported by Crepaz et al. [35]. The table below shows the target diagnosis rate for each demographic group and the final calibrated testing probabilities.

| Table 12. Annual diagnoses rates per 100K by demographic group | | | |
| --- | --- | --- | --- |
| **Demographic Group** | | **Annual diagnosis per/100K** | **Post-calibration testing parameters** |
| MSM | Black | 574.7 | 0.0146 |
|  | Hispanic |  | 0.0156 |
|  | White/Other |  | 0.0113 |
| Male Heterosexual | Black | 16.6 | 0.0019 |
|  | Hispanic | 3.2 | 0.0004 |
|  | White/Other | .07 | 0.0002 |
| Female Heterosexual | Black | 26.1 | 0.0022 |
|  | Hispanic | 5.5 | 0.0006 |
|  | White/Other | 1.4 | 0.0001 |

Figures two and three show the simulated distribution of diagnosis rates across 50 simulations for the heterosexual and MSM populations respectively. The range of the boxplots is an indication of the overall heterogeneity in our simulations. The target values found in the literature are indicated in red. The simulated testing rates generally reproduced the target statistics with a under prediction for diagnosis among heterosexual black males.

**Figure 2** Diagnosis rates for each of the heterosexual demographic groups with the target rate indicated in red.


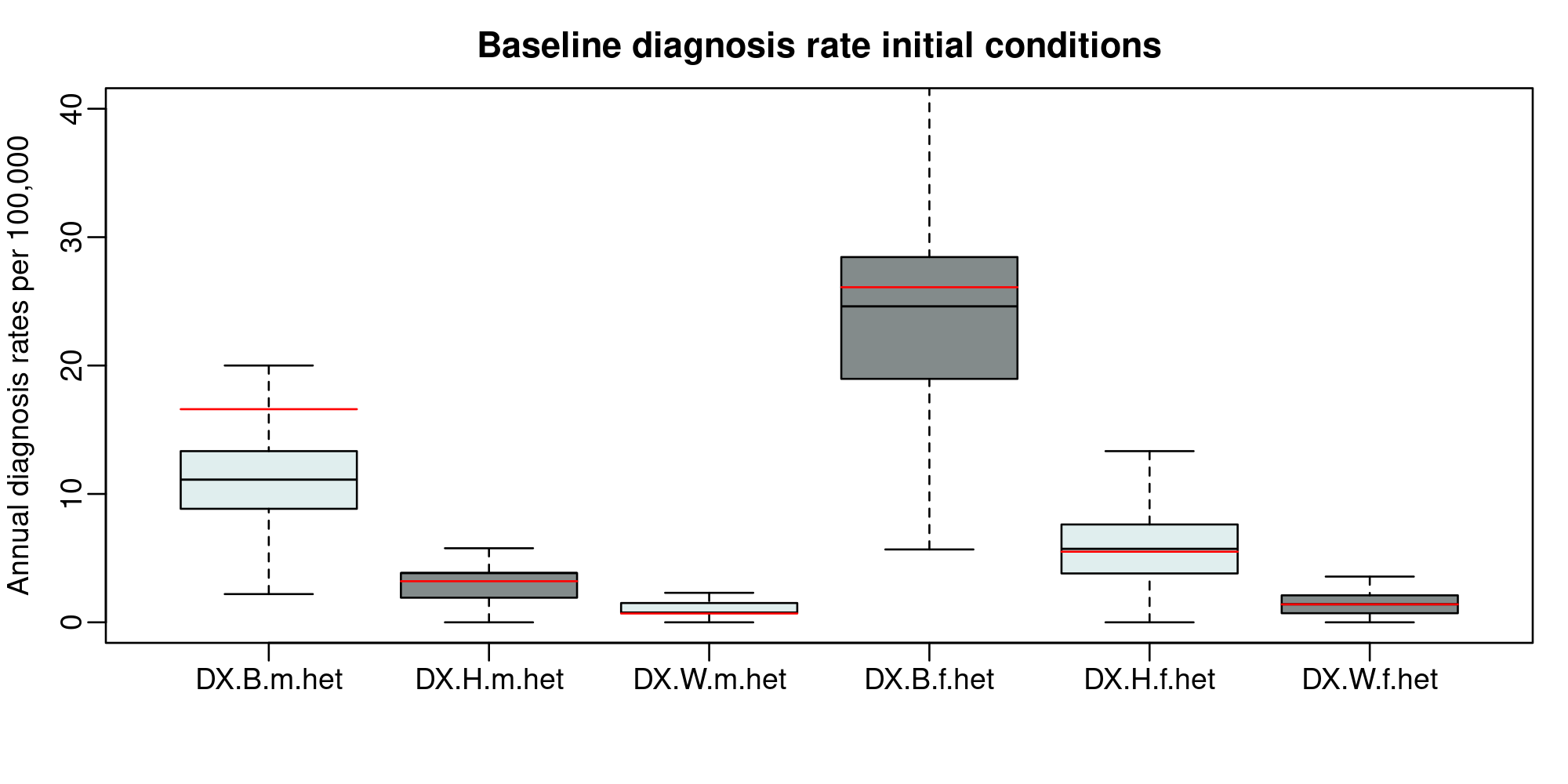


**Figure 3** Diagnosis rates for MSM with the target rate indicated in red


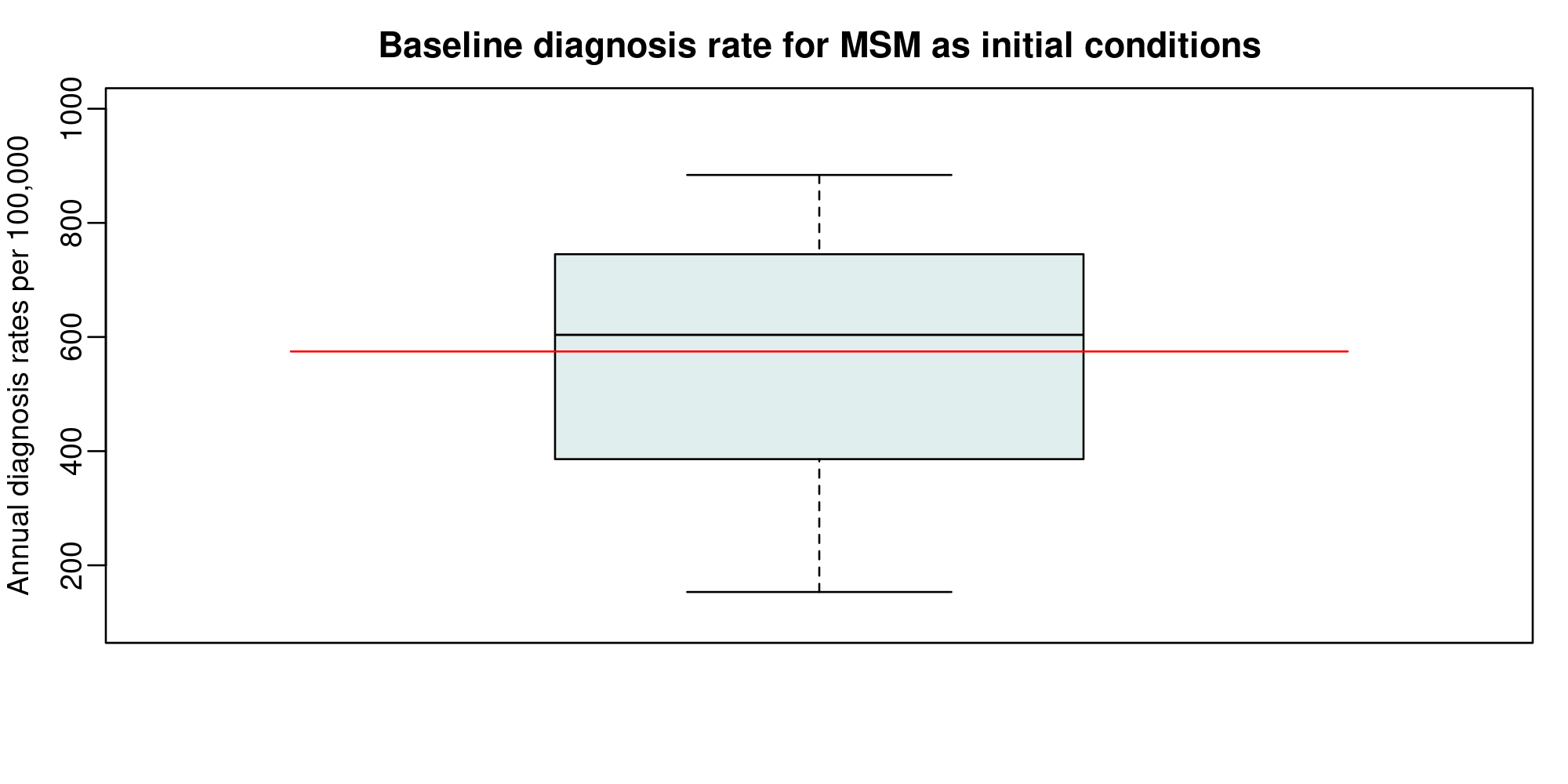


## Antiretroviral Therapy (ART) Initiation and Adherence

We simulated the initiation of ART and subsequent clinical outcomes of full or partial HIV viral suppression based on nodes being in one of three clinical states: never tested/undiagnosed, on treatment and partially virally suppressed, and on treatment with full viral suppression. There were insufficient empirical data to represent the patterns and rates at which individual switch among these three states over the course of their infection, since the clinical ART landscape is constantly evolving. Therefore, we modeled nodes as being on one of two fixed treatment trajectories as an individual-level attribute. Individuals initiating ART were designated as either full or partial suppressors.

Following diagnosis, ART initiation rates were specific to each of the nine demographic groups discussed previously. These rates were calibrated to match the reported linkage to care reported in AHEAD in 2019. [36] In the AHEAD reports linkage to HIV medical care was measured by documentation of ≥1 CD4 or VL tests ≤1 month after HIV diagnosis. Percentages of diagnosed individuals linked to care within ≤1 month were reported by gender (Additional gender identity, Female, Male, Transgender male-to-female, Transgender female-to-male), by race (Asian, Black/African American, Hispanic/Latinx, multiple races, Native Hawaiian / Other Pacific Islander, Other, and White), and by transmission category (Heterosexual contact-female, Heterosexual contact-male, Injection drug use-female, Injection drug use-male, Male-to-male sexual contact, Male-to-male sexual contact and injection drug use, Other-female and Other-male). In order to convert these categories into the nine demographic categories in our model we use the percentage linked by transmission category to determine our baseline proportion of diagnosed individuals linked to care for heterosexual females, heterosexual males and males who have sex with males. For males that have sex with both males and females, we based the proportion linked to care on the proportion of males who have sex with males. These proportions where then adjusted across race categories using the relative proportions reported for the Black/African American and Hispanic/Latino race/ethnicity groups with White as the referent category. The target proportions and the ART initiation rates after calibration are shown in the table below.

| Table 13. Linkage to HIV care and treatment | | | |
| --- | --- | --- | --- |
| **Demographic group** | | **Percentage linked within 1 month** | **Rate of ART initiation** |
| MSM | Black | 77.0% | 0.270 |
|  | Hispanic | 83.9% | 0.299 |
|  | Other | 82.1% | 0.304 |
| Male Heterosexual | Black | 76.2% | 0.237 |
|  | Hispanic | 81.9% | 0.276 |
|  | Other | 80.1% | 0.237 |
| Female Heterosexual | Black | 77.0% | 0.269 |
|  | Hispanic | 82.9% | 0.323 |
|  | Other | 81.1% | 0.278 |

**Figure 4** The proportion linked to care within 1 month over the course of eight years of simulation across 50 simulations by demographic group.


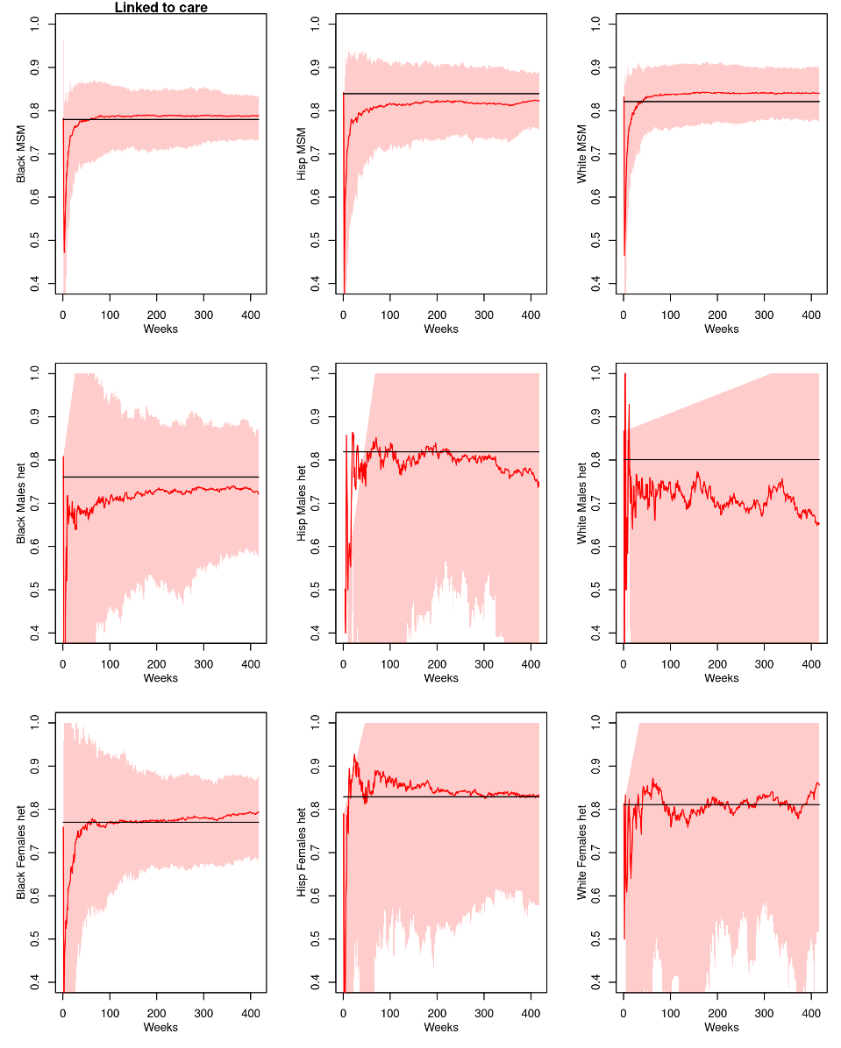


These results represent linkage to care in our baseline model. The red line indicates the mean proportion linked to care within <= 1 month for each demographic group during the 8 years of simulation. The red shaded regions indicate the 95% simulation interval (SI) and the black lines indicate the target value.

*Viral Suppression*

The AHEAD report also provided estimates of the proportion of those diagnosed with HIV that are virally suppressed which they define as a VL test result of <200 copies/mL. Viral suppression was reported for the same categories as linkage to care so we used the same approach for converting the reported suppression proportions to proportions for the nine demographic groups in our model. Only individuals that are full suppressors achieve viral loads below <200 copies/mL in our model. Full suppressors also live longer due to lower viral loads. In order to match the proportions of diagnosed individuals within demographic group that are virally suppressed we calibrated our simulations by adjusting the proportions of individuals that are full vs. partial suppressors. The target proportions from the AHEAD estimates and the proportions in the simulated population designated as full suppressors are shown in the table below.

| Table 13. Proportion Virally Suppressed | | |
| --- | --- | --- |
| Demographic group |  | Target Proportion Virally Suppressed |
| MSM | Black | 64.09 |
|  | Hispanic | 61.56 |
|  | Other | 68.10 |
| Male Heterosexual | Black | 56.94 |
|  | Hispanic | 54.69 |
|  | Other | 60.50 |
| Female Heterosexual | Black | 61.27 |
|  | Hispanic | 58.85 |
|  | Other | 65.10 |

**Figure 5** The proportion of diagnosed individuals that were virally suppressed over the course of eight years of simulation by demographic group.


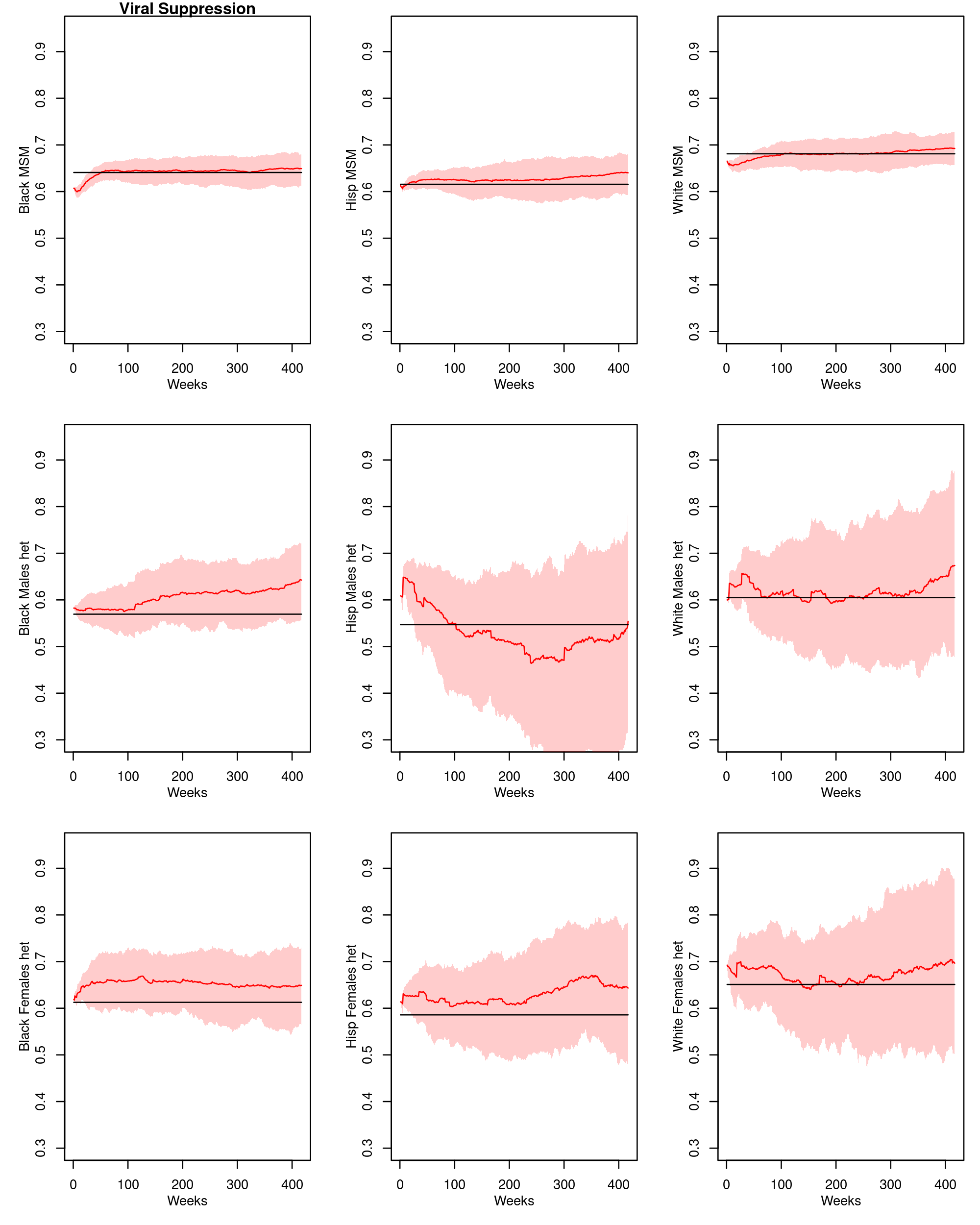


These results represent the proportion of those on ART that are virally suppressed in our baseline model. The red line indicates the mean proportion virally suppressed for each demographic group. The red shaded regions indicate the 95% SI and the black lines indicate the target value.

*ART coverage and retention*

Overall ART coverage targets were calculated from the AIDSvue State-level reports on the continuum of care.[37] The data for the southern states and the proportion of cases who have had at least 1 CD4/VL test in past year where weighted to account for the difference in the number of diagnosed individuals in each state which generated an estimated 75.6% of those diagnosed engaged in care which we use as a proxy for being on ART. Our model was calibrated to fit overall ART coverage by adjusting the rate of ART discontinuation and re-initiation. ART initiation was driven by demographic specific rates of linkage to care within one month as described previously. In the calibrated model individuals discontinued ART at a rate of 0.00123 and re-initiated ART at a rate of 0.00235. The baseline level of ART coverage is shown in figure 6. The red line indicates the mean proportion on ART, the red shaded regions indicate the 95% SI and the black line indicates the target value.

**Figure 6** The proportion of diagnosed individuals that are on ART over the course of eight years of simulation.


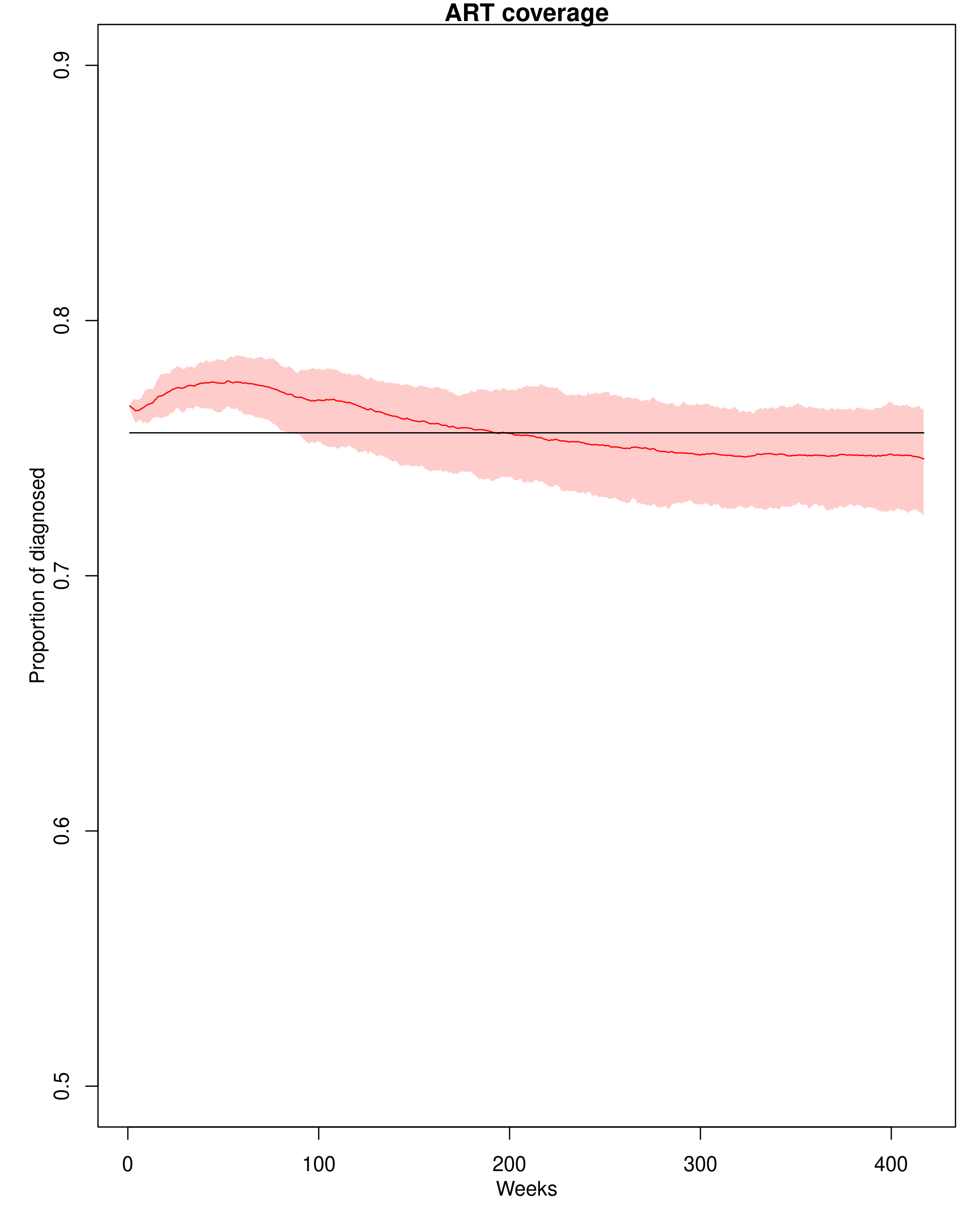


After calibrating our model to fit demographic-group specific diagnosis rates and linkage to care as well as the overall level of ART coverage the level of ART coverage for each demographic group, shown in figure 7, became an outcome of the simulation process. These demographic-specific levels of ART coverage are the focus of the hypothetical ART interventions modeled in this study.

**Figure 7** The proportion of HIV+ individuals that are on ART over the course of eight years of simulation for each of the 9 demographic groups.

#
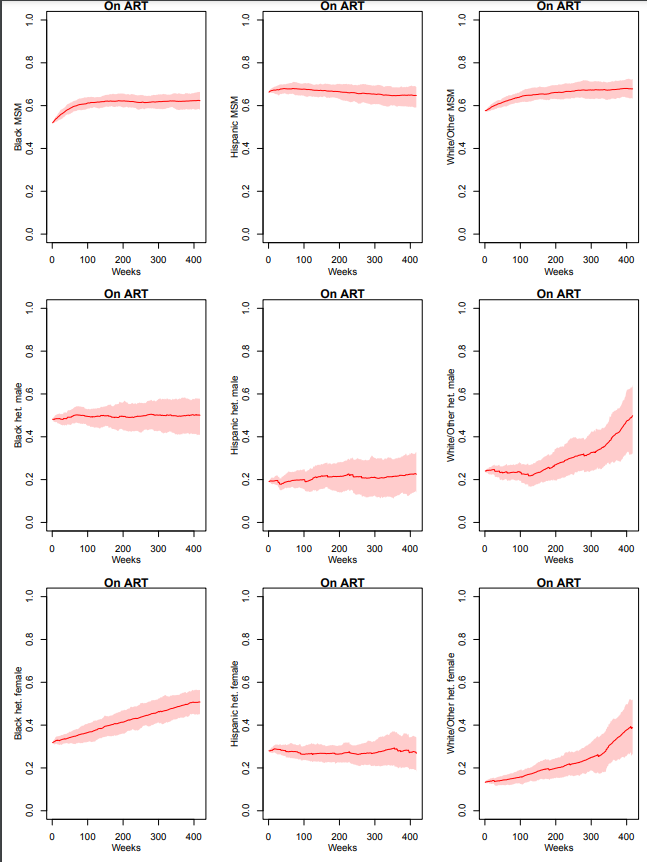


# *PrEP coverage and retention*

MSM in the simulation were eligible for PrEP if they were HIV- and either in a monogamous relationship with a partner who had not tested in past 6 months or they were in 2 or more concurrent relationships. Heterosexuals in the simulation were eligible for PrEP if they were HIV- and either in a casual relationship with a partner who had not tested in 6 months or they were in 2 or more concurrent relationships. In practice a primary indicator for PrEP is a positive diagnosis for STI, however, in this simulation the only STI included is HIV.

The baseline level of PrEP coverage was based on findings from Siegler el al. [38] who reported 21 PrEP users per 100,000 individuals in the South in Q4 of 2017 overall and a rate of 1.9/100,000 among females. We assumed that the rate of PrEP use among heterosexual males more closely resembles that of females than MSM and used the 1.9/100,000 rate for heterosexual males as well. After determining the expected number of heterosexual males and females on PrEP using these rates we subtracted the heterosexuals from the total number of reported PrEP users and used the remainder to determine the rate for MSM (1351.2/100,000). To account for differences in PrEP use by race we used the proportions of indicated individuals currently using PrEP reported in the HIV Surveillance Supplemental report.[39]; (5.9 Black, 10.9 Hispanic, 42.1 White/Other) and applied the relative proportions to the three different race groups for the MSM, heterosexual males and heterosexual females. Once PrEP was initiated adherence was specific to MSM and heterosexuals. MSM had high, moderate and low adherence at rates of 0.784, 0.127 and 0.089 respectively based on adherence reported by Lui et al. [40] while heterosexuals had high, moderate and low adherence at rates of 0.66, 0.13 and 0.21 based on reports from Blumenthal et al.[41] The MSM in the model had race specific probabilities of discontinuation 0.015, 0.012 and 0.006 for Black/AA, Hispanic and White/Other respectively[40]. Race specific estimates for heterosexual males and females were not available, however Huang et al. reported a PrEP discontinuation rate 0.025 among females[42]. The reported discontinuation rate for males was not used because it does not differentiate between heterosexual males and MSM and PrEP use by heterosexual males is likely to be more similar to PrEP use by heterosexual females than MSM. Differences between heterosexual males and females were based on the 2.8 relative risk of discontinuation reported for females by Marcus et al. [43] which yielded an 0.07 weekly rate of discontinuation among heterosexual males.

# INTERHOST EPIDEMIOLOGY

Interhost epidemiological processes represent the HIV-1 disease transmission within the model. Disease transmission occurred between sexual partners who were active on a given time step. This section describes the overall rate as a function of the intrahost epidemiological profile of each member of a partnership and behavioral features within the dyad.

## Disease-Discordant Dyads

At each time step in the simulation, dyads that were discordant with respect to HIV status had the potential to transmit an infection from the HIV positive partner to the HIV negative partner at that time step.

## HIV Transmission

Within HIV-discordant dyads, transmission was simulated stochastically across separate sexual acts at each timestep. The per-act probabilities were a combined function of attributes of the HIV-negative and HIV-positive partner. Final per-partnership transmission rates per time step were then a function of these per-act transmission probabilities raised to the number of acts within the partnership during that time step.

These probabilities were calibrated to reach the empirical estimated HIV prevalence and incidence. The empirical targets for both prevalence and incidence were drawn from the literature. Based on data from the HIV Surveillance supplemental report [44] the prevalence of HIV in the South was 539,600 persons or 517.4 per 100,000 population which translated to 2587 individuals in our simulated population of 500K. The CDC report did not breakout the number of individuals living with HIV in the South by demographic groups but the number of individuals living with HIV in the US was provided so we use the national data to determine the relative distributions of the cases reported for the South. Based on the National distribution 63.3% of individuals living with HIV are MSM and 36.7% are heterosexual. In both cases we combined the MSM and Heterosexual transmission categories to include those with sexual contact risk and IDU. The distribution of persons living with HIV were 44% Black/AA, 25% Hispanic/Latino and 31% Other/White. We used this distribution by race to determine race specific prevalence among the heterosexual population in our model. More specific data was available for MSM living with HIV. CDC estimates that among MSM 33.5% of individuals living with HIV are Black/AA, 25.7% are Hispanic/Latino and 40% are Other/White. We used this MSM specific race distribution to determine the target prevalence by race among the MSM in our simulation. Table 14 provided the target distribution of individuals living with HIV in our simulation as well as the starting values after model calibration.

| Table 14. Individuals living with HIV by demographic category | | | | | |
| --- | --- | --- | --- | --- | --- |
| Demographic group |  | N | % of individuals living with HIV | Starting prevalence values in the calibrated simulation | Starting % of individuals living with HIV |
| MSM | Black/AA | 549 | 21.2% | 581 | 22.6% |
|  | Hispanic | 421 | 16.3% | 439 | 17.1% |
|  | Other/White | 667 | 25.8% | 647 | 25.2% |
| Male Heterosexual | Black/AA | 164 | 6.4% | 166 | 6.5% |
|  | Hispanic | 93 | 3.6% | 94 | 3.7% |
|  | Other/White | 116 | 4.5% | 103 | 4.0% |
| Female Heterosexual | Black/AA | 254 | 9.8% | 242 | 9.4% |
|  | Hispanic | 144 | 5.6% | 132 | 5.1% |
|  | Other/White | 179 | 6.9% | 164 | 6.4% |
| Total |  | 2587 | 100% | 2568 | 100% |

The HIV incidence targets were taken directly from the CDC HIV incidence reports for 2018 [44]. The overall incidence rate in the south was 18.4 cases per 100K. The rates for males and females were 22.2 and 4.8 per 100K respectively. The incidence rates by race were reported as 45.4, 22.4 and 5.2 per 100K for Black/AA, Hispanic/Latino and Other/White respectively. Finally, the proportion of incident cases was 71% MSM and 29% heterosexual contact. Because the overall rate we used in our analysis is specific to the South but the rates by race and proportions by transmission category are national it is not expected that these parameter targets will be internally consistent. As such we used the incidence rate among males and females as well as the ratio of MSM to heterosexuals as our primary targets which resulted in a lower incidence rate overall. Table 14 shows the baseline incidence rate by demographic group. The calibrated incidence rates closely matched the rates for males and females and the proportion of incident cases that were among MSM also closely aligned with the CDC reports (see figures 8 & 9). The overall incidence in the simulation was somewhat lower than the CDC report for the South, however the relative under-fitting was uniform across all race groups. In addition, the proportion of incident infections among MSM in the simulation was within 4% of reported estimates.

| Table 14. HIV incidence per 100,000 | | | |
| --- | --- | --- | --- |
| Demographic group | Target value | Mean incidence rate and 95% SI | Percent difference |
| All | 18.4 | 12.7 (95%SI: 10.1, 14.8) | 31% |
| Males | 22.1 | 20.5 (95%SI: 15.3, 24.5) | **7%** |
| Females | 4.8 | 4.7 (95%SI: 3.0, 7.0) | **2%** |
| Black | 45.4 | 30.7 (95%SI: 21.7, 39.9) | 32% |
| Hispanic | 22.2 | 17.7 (95%SI: 12.2, 24.3) | 20% |
| White/Other | 5.2 | 4.2 (95%SI: 2.5, 6.4) | 19% |
| MSM/Hetersexual ratio | 0.71 | 0.68 (95%SI: 0.55, 0.76) | **4%** |

**Figure 8** Annual HIV incidence per 100,000 individuals over the course of eight years of simulation for each of the 6 demographic groups.


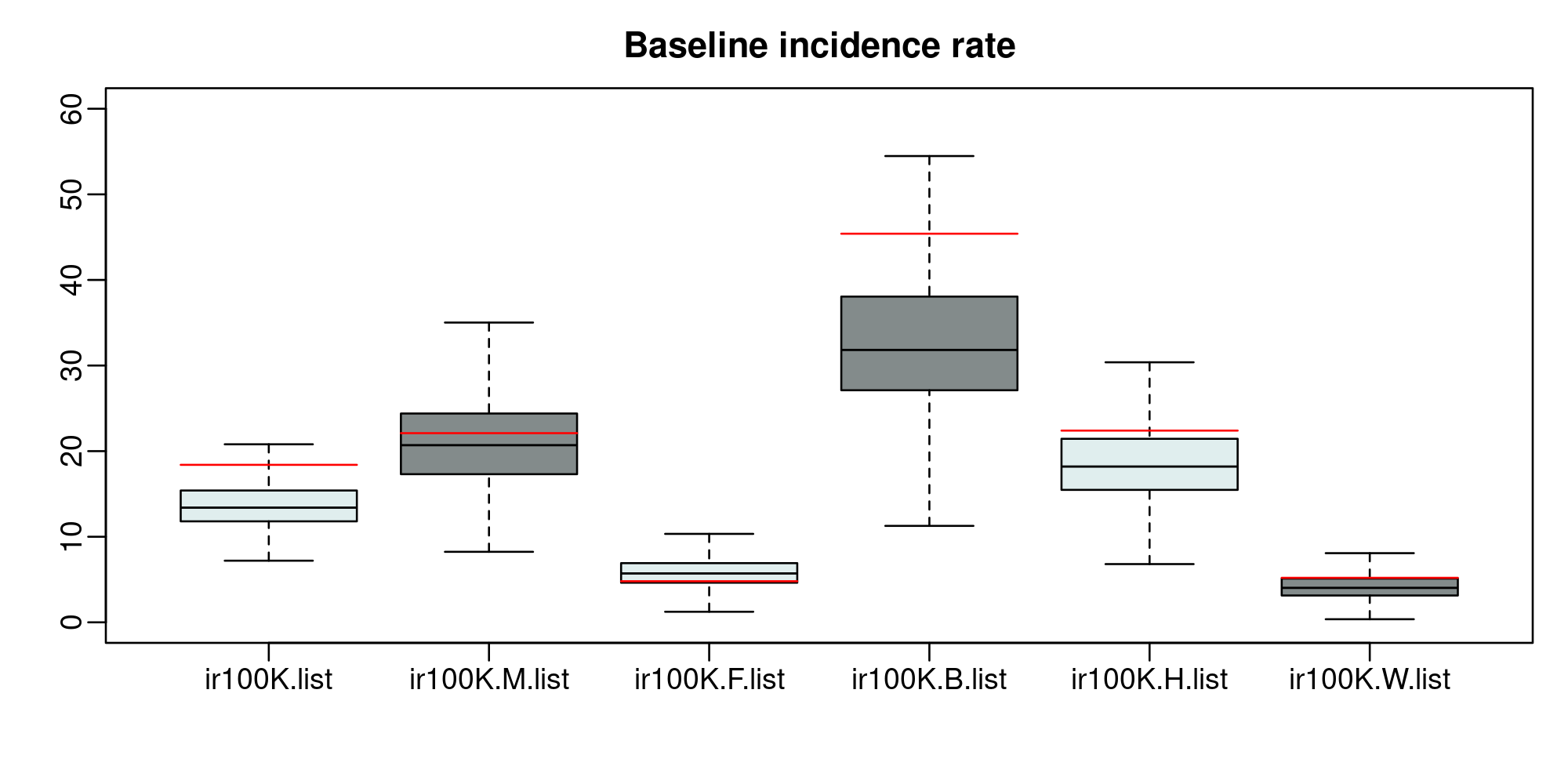


**Figure 9** Proportion of incident HIV infections among MSM over the course of eight years of baseline simulation


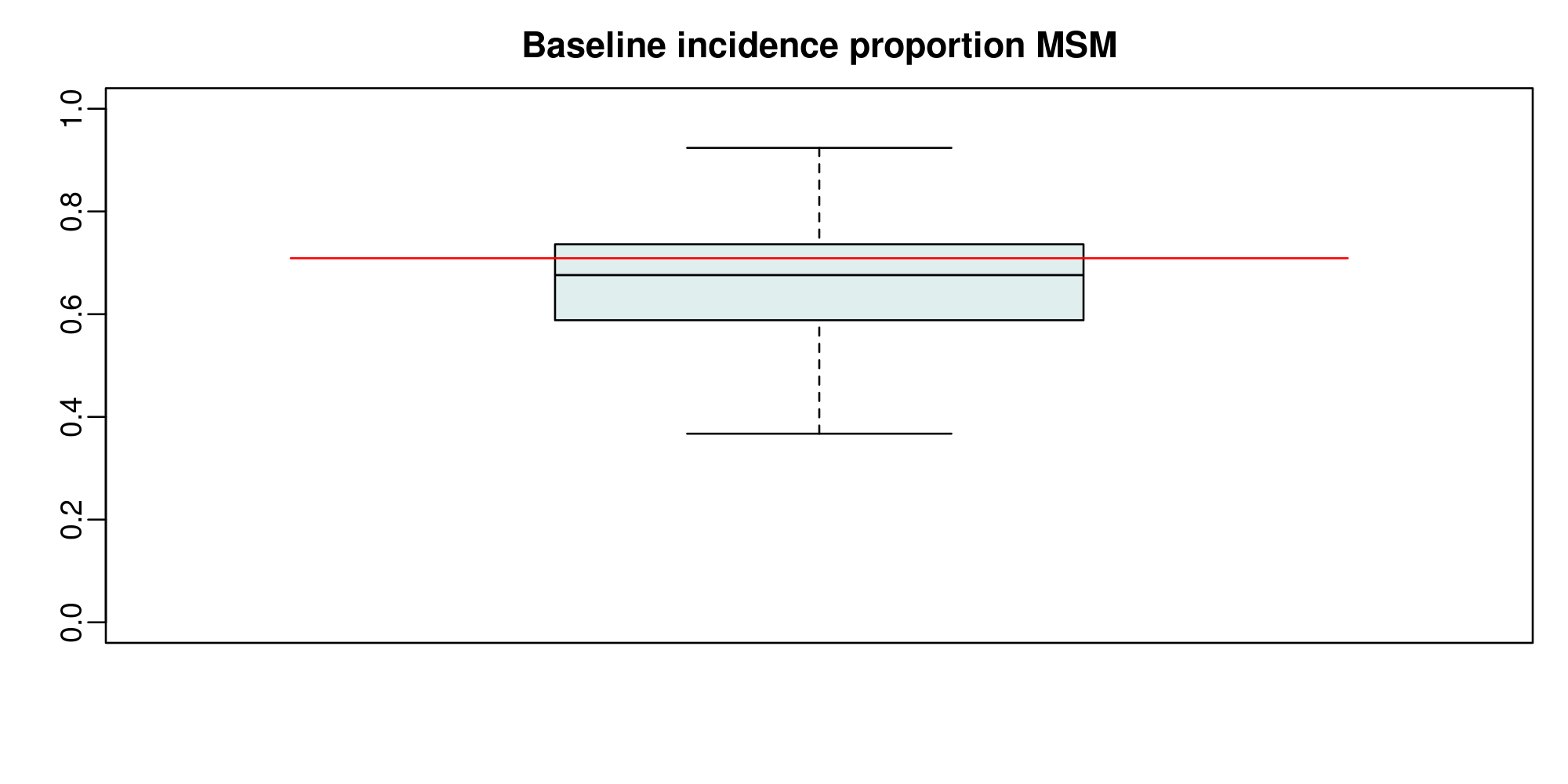


Per Act probabilities

HIV transmission was modeled based on a sexual act-by-act basis, in which multiple acts of varying infectiousness could occur within one partnership within a weekly time step. The determination of the number of acts within each dyad for each time step, as well as condom use and role for each of those acts, was described above. Transmission by act was then modeled as a stochastic process for each discordant sex act following a binomial distribution with a probability parameter that is a multiplicative function of the following predictors of the HIV- and HIV+ partners within the dyad, as shown in Table 15 below. For each act, the overall transmission probability was determined first based on the type of sex (penile-vaginal or penile-anal), sexual position and HIV viral suppression status of the infected partner. If the infected partner was virally suppressed and on ART, then the base probability was 2.2/100,000, which was derived from a model-based estimate of Supervie [45]. This study estimated an upper bound of the transmission probability of 4.4/100,000 for MSM and 3.9/100,000 for heterosexuals. Following Jennesse et al. [8] we used the mean between the observed number (zero) and the upper bound reported for MSM as our base per-act transmission probability (so 2.2 transmissions per 100,000 exposures) in our model. We used the same value for both MSM and heterosexuals as the 2.2/100,000 was not qualitatively dissimilar to the mean 1.5/100,000 between the observed number (one) and the upper bound reported for heterosexuals. If the infected partner was not virally suppressed (viral load of 200 copies/mL or higher) or not currently on ART, the base probability was a function of whether the HIV- partner was in the receptive or insertive role, and whether the act was penile-anal or penile-vaginal. The per-act transmission probabilities are shown in the table 15. Then, following the parametric function of Wilson [46] the HIV+ partner’s viral load modifies this base probability in a non-linear formulation, upwards if the VL was above the VL set point during chronic stage infection in the absence of ART, and downwards if it was below the set point. Following others, we modeled an excess transmission risk in the acute stage of infection above that predicted by the heightened VL during that period.[47] Three covariates could reduce the risk of infection: condom use within the act by either the HIV- or HIV+ partner, circumcision status of the HIV- partner (only if the HIV- partner was insertive in that act), and PrEP use at the time of the act by the HIV- partner. The impact of PrEP on the probability of transmission was conditional on PrEP adherence which could be high, medium or low. The associated multipliers for the impact of PrEP were drawn from Gray et al. and were consistent for all demographic groups, however the distribution of adherence levels differed between MSM and heterosexuals. For MSM, 78.4%, 12.7% and 8.9% were high, moderate and low adherence respectively compared to 66%, 13% and 21% for the heterosexuals based on reports from Blumenthal et al.[41]

| **Table 15** Per-act transmission probabilities | | | |
| --- | --- | --- | --- |
| **Predictor** | **Partner** | **Parameters** | **References** |
| Sex Act | HIV – receptive male | 0.0194 | Infectious Disease Prevention and Control[48] |
|  | HIV – insertive male with male partner | 0.0011 | Infectious Disease Prevention and Control[48] |
|  | HIV – receptive females | 0.0008 | Infectious Disease Prevention and Control[48] |
|  | HIV – insertive male with female partner | 0.0005 | Infectious Disease Prevention and Control[48] |
| Acute stage | HIV-positive | Multiplier of 6.0 | Leynaert [49] Bellan [47] |
| Condom use | Both | Multiplier of 0.05 | Varghese [50] Weller [51]  Smith[52] |
| Circumcision status | HIV-negative, male | Multiplier of 0.40 | Gray[29] |
| Preexposure Prop[hylaxis | HIV-negative | High  adherence: Multiplier of 0.01  Medium adherence: Multiplier of 0.19  Low adherence: Multiplier of 0.69 | Grant [53] |

Calibration of transmission probabilities

In addition to the calibration of the HIV care continuum parameters described above, we also calibrated the per-act transmission probabilities so that both HIV prevalence and HIV incidence were consistent with empirical data discussed above. Calibration was conducted in two phases, first the overall transmission rates were adjusted by one multiplier and then the per-act transmission probabilities defined above were multiplied by a factor unique to each of the 9 demographic categories. The overall transmission probabilities were increased by a factor of 10. The final group factor levels were (0.2798, 0.0913, 0.0226, 1.1264, 0.7917, 0.0239, 0.2856, 0.7817, 0.0126) for Black, Hispanic and White/Other MSM, heterosexual males and heterosexual females. These calibration factors represent the additional sources of potential error in the transmission parameters that would generate the current HIV epidemic. These include co-factors not included in this model, such as untreated sexually transmitted infections.[54-57] Among both the MSM and heterosexual males the Black/AA demographic groups had higher weights than either the Hispanic or White/Other groups. Among the heterosexual females the Hispanic group had the highest weight but the Black/AA group was also weighted much higher than their White/Other counterparts. This is consistent with long-standing findings that race-stratified behavioral and network data do not, by themselves, explain the excess burden of HIV among Black/AA populations [58-63]. The post calibration incidence rates are shown in figure 7 above.

# SIMULATION METHODS

## Intervention Simulations

The intervention scenarios are described fully within the main paper. For each scenario, we simulated the model scenario 50 times for 8 calendar years representing 2022 through 2030 in each simulation. Data from each simulation scenario were merged, and a complete 50-simulation data file was retained for each scenario. We ran 50 hypothetical ART scenarios that increase ART coverage to 60%, 70%, 80%, 90% and 100% for all HIV positive individuals within each of the 9 demographic groups and then for all demographic groups. We also ran 50 hypothetical PrEP scenarios that increase PrEP coverage to 10%, 20%, 30%, 40% and 50% for all PrEP eligible individuals within each of the 9 demographic groups and then for all demographic groups. The two interventions where then combined to determine the joint effects. An additional 15 scenarios were run adjusting both ART and PrEP coverage for all demographic groups simultaneously. In the 15 combined scenarios 5 increased ART coverage and PrEP coverage together along the same ranges used in the prior scenarios, 5 fixed ART coverage at 90% and increased PrEP coverage, and the last 5 fixed ART at 95% and increased PrEP coverage.

1. Handcock MS, Hunter DR, Butts CT, Goodreau SM, Morris M. statnet: Software Tools for the Representation, Visualization, Analysis and Simulation of Network Data. J Stat Softw. 2008;24(1):1548-7660. doi: 10.18637/jss.v024.i01. PubMed PMID: 18618019; PubMed Central PMCID: PMCPMC2447931.

2. Goodreau SM, Carnegie NB, Vittinghoff E, Lama JR, Fuchs JD, Sanchez J, et al. Can male circumcision have an impact on the HIV epidemic in men who have sex with men? PLoS One. 2014;9(7):e102960. doi: 10.1371/journal.pone.0102960. PubMed PMID: 25076493; PubMed Central PMCID: PMCPMC4116164.

3. Goodreau SM, Carnegie NB, Vittinghoff E, Lama JR, Sanchez J, Grinsztejn B, et al. What drives the US and Peruvian HIV epidemics in men who have sex with men (MSM)? PLoS One. 2012;7(11):e50522. doi: 10.1371/journal.pone.0050522. PubMed PMID: 23209768; PubMed Central PMCID: PMCPMC3510067.

4. Hamilton DT, Goodreau SM, Jenness SM, Sullivan PS, Wang LY, Dunville RL, et al. Potential Impact of HIV Preexposure Prophylaxis Among Black and White Adolescent Sexual Minority Males. American Journal of Public Health. 2018;108(S4):S284-S91. doi: 10.2105/ajph.2018.304471. PubMed PMID: 30383415.

5. Hamilton DT, Rosenberg ES, Jenness SM, Sullivan PS, Wang LY, Dunville RL, et al. Modeling the joint effects of adolescent and adult PrEP for sexual minority males in the United States. PLoS One. 2019;14(5):e0217315. doi: 10.1371/journal.pone.0217315. PubMed PMID: 31116802; PubMed Central PMCID: PMCPMC6530873.

6. Hamilton DT, Rosenberg ES, Sullivan PS, Wang LY, Dunville RL, Barrios LC, et al. Modeling the Impact of PrEP Programs for Adolescent Sexual Minority Males Based on Empirical Estimates for the PrEP Continuum of Care. J Adolesc Health. 2020. doi: 10.1016/j.jadohealth.2020.06.041. PubMed PMID: 32798099.

7. Luo W, Katz DA, Hamilton DT, McKenney J, Jenness SM, Goodreau SM, et al. Development of an Agent-Based Model to Investigate the Impact of HIV Self-Testing Programs on Men Who Have Sex With Men in Atlanta and Seattle. JMIR Public Health Surveill. 2018;4(2):e58. doi: 10.2196/publichealth.9357. PubMed PMID: 29959112; PubMed Central PMCID: PMCPMC6045793.

8. Jenness SM, Johnson JA, Hoover KW, Smith DK, Delaney KP. Modeling an integrated HIV prevention and care continuum to achieve the Ending the HIV Epidemic goals. AIDS. 2020;34(14):2103-13. doi: 10.1097/QAD.0000000000002681. PubMed PMID: 32910062; PubMed Central PMCID: PMCPMC7606461.

9. Jenness SM, Goodreau SM, Rosenberg E, Beylerian EN, Hoover KW, Smith DK, et al. Impact of the Centers for Disease Control's HIV Preexposure Prophylaxis Guidelines for Men Who Have Sex With Men in the United States. J Infect Dis. 2016;214(12):1800-7. doi: 10.1093/infdis/jiw223. PubMed PMID: 27418048; PubMed Central PMCID: PMCPMC5142082.

10. Maloney KM, Le Guillou A, Driggers RA, Sarkar S, Anderson EJ, Malik AA, et al. Projected Impact of Concurrently Available Long-Acting Injectable and Daily-Oral Human Immunodeficiency Virus Preexposure Prophylaxis: A Mathematical Model. J Infect Dis. 2021;223(1):72-82. doi: 10.1093/infdis/jiaa552. PubMed PMID: 32882043.

11. Centers for Disease Control and Prevention National Center for Health Statistics. 2011-2013 NSFG: Public Use Data Files, Codebooks, and Documentation. Available from: <https://www.cdc.gov/nchs/nsfg/nsfg_2011_2013_puf.htm>.

12. Centers for Disease Control and Prevention National Center for Health Statistics. 2013-2015 NSFG: Public Use Data Files, Codebooks, and Documentation. Available from: <https://www.cdc.gov/nchs/nsfg/nsfg_2013_2015_puf.htm>.

13. Centers for Disease Control and Prevention National Center for Health Statistics. 2015-2017 NSFG: Public-Use Data Files, Codebooks, and Documentation. Available from: <https://www.cdc.gov/nchs/nsfg/nsfg_2015_2017_puf.htm>.

14. Weiss KM, Goodreau SM, Morris M, Prasad P, Ramaraju R, Sanchez T, et al. Egocentric sexual networks of men who have sex with men in the United States: Results from the ARTnet study. Epidemics. 2020;30:100386. doi: 10.1016/j.epidem.2020.100386. PubMed PMID: 32004795; PubMed Central PMCID: PMCPMC7089812.

15. Zlotorzynska M, Sullivan P, Sanchez T. The Annual American Men's Internet Survey of Behaviors of Men Who Have Sex With Men in the United States: 2016 Key Indicators Report. JMIR Public Health Surveill. 2019;5(1):e11313. doi: 10.2196/11313. PubMed PMID: 30785405; PubMed Central PMCID: PMCPMC6401665.

16. Centers for Disease Control and Prevention National Center for Health Statistics. Combining Data across NSFG File Releases from 2011-2019. Available from: <https://www.cdc.gov/nchs/nsfg/nsfg_combining_data.htm>.

17. Census US. [cited 2020]. Available from: <https://data.census.gov/cedsci/>.

18. Grey JA, Bernstein KT, Sullivan PS, Purcell DW, Chesson HW, Gift TL, et al. Estimating the Population Sizes of Men Who Have Sex With Men in US States and Counties Using Data From the American Community Survey. JMIR Public Health Surveill. 2016;2(1):e14. doi: 10.2196/publichealth.5365. PubMed PMID: 27227149; PubMed Central PMCID: PMCPMC4873305.

19. Centers for Disease Control and Prevention. National Survey of Family Growth 2021 [cited 2021 Sept. 29]. Available from: <https://www.cdc.gov/nchs/nsfg/index.htm>.

20. Dasgupta S, Tie Y, Bradley H, Beer L, Rosenberg ES, Holtgrave D, et al. Characteristics of Sexual Partnerships Among Men With Diagnosed HIV Who Have Sex With Men, United States and Puerto Rico-2015-2019. J Acquir Immune Defic Syndr. 2020;84(5):443-52. doi: 10.1097/QAI.0000000000002388. PubMed PMID: 32692102.

21. Jenness SM, Goodreau SM, Morris M. EpiModel: An R Package for Mathematical Modeling of Infectious Disease over Networks. J Stat Softw. 2018;84. doi: 10.18637/jss.v084.i08. PubMed PMID: 29731699; PubMed Central PMCID: PMCPMC5931789.

22. Krivitsky PN, Handcock MS. A Separable Model for Dynamic Networks. J R Stat Soc Series B Stat Methodol. 2014;76(1):29-46. doi: 10.1111/rssb.12014. PubMed PMID: 24443639; PubMed Central PMCID: PMCPMC3891677.

23. Hunter DR, Handcock MS, Butts CT, Goodreau SM, Morris M. ergm: A Package to Fit, Simulate and Diagnose Exponential-Family Models for Networks. J Stat Softw. 2008;24(3):nihpa54860. doi: 10.18637/jss.v024.i03. PubMed PMID: 19756229; PubMed Central PMCID: PMCPMC2743438.

24. Carnegie NB, Krivitsky PN, Hunter DR, Goodreau SM. An approximation method for improving dynamic network model fitting. J Comput Graph Stat. 2015;24(2):502-19. doi: 10.1080/10618600.2014.903087. PubMed PMID: 26321857; PubMed Central PMCID: PMCPMC4548897.

25. Krivitsky PN, Handcock MS, Morris M. Adjusting for Network Size and Composition Effects in Exponential-Family Random Graph Models. Stat Methodol. 2011;8(4):319-39. doi: 10.1016/j.stamet.2011.01.005. PubMed PMID: 21691424; PubMed Central PMCID: PMCPMC3117581.

26. Eaton LA, Kalichman SC. Changes in transmission risk behaviors across stages of HIV disease among people living with HIV. J Assoc Nurses AIDS Care. 2009;20(1):39-49. doi: 10.1016/j.jana.2008.10.005. PubMed PMID: 19118770; PubMed Central PMCID: PMCPMC3560412.

27. Hollingsworth TD, Anderson RM, Fraser C. HIV-1 transmission, by stage of infection. J Infect Dis. 2008;198(5):687-93. doi: 10.1086/590501. PubMed PMID: 18662132.

28. Morris BJ, Bailis SA, Wiswell TE. Circumcision rates in the United States: rising or falling? What effect might the new affirmative pediatric policy statement have? Mayo Clin Proc. 2014;89(5):677-86. doi: 10.1016/j.mayocp.2014.01.001. PubMed PMID: 24702735.

29. Gray RH, Kigozi G, Serwadda D, Makumbi F, Watya S, Nalugoda F, et al. Male circumcision for HIV prevention in men in Rakai, Uganda: a randomised trial. The Lancet. 2007;369(9562):657-66. doi: 10.1016/s0140-6736(07)60313-4.

30. Arias E, Heron M, Xu J. United States Life Tables, 2012. Natl Vital Stat Rep. 2016;65(8):1-65. PubMed PMID: 27906644.

31. Goodreau SM, Hamilton DT, Jenness SM, Sullivan PS, Valencia RK, Wang LY, et al. Targeting Human Immunodeficiency Virus Pre-Exposure Prophylaxis to Adolescent Sexual Minority Males in Higher Prevalence Areas of the United States: A Modeling Study. J Adolesc Health. 2018;62(3):311-9. doi: 10.1016/j.jadohealth.2017.09.023. PubMed PMID: 29248392; PubMed Central PMCID: PMCPMC5818296.

32. Robb ML, Eller LA, Kibuuka H, Rono K, Maganga L, Nitayaphan S, et al. Prospective Study of Acute HIV-1 Infection in Adults in East Africa and Thailand. N Engl J Med. 2016;374(22):2120-30. doi: 10.1056/NEJMoa1508952. PubMed PMID: 27192360; PubMed Central PMCID: PMCPMC5111628.

33. Buchbinder S, Katz M, Hessol N, O’Malley P, Holmberg S. Long-term HIV-1 infection without immunologic progression. AIDS. 1994;8.

34. Katz MH, Hessol NA, Buchbinder SP, Hirozawa A, O'Malley P, Holmberg SD. Temporal trends of opportunistic infections and malignancies in homosexual men with AIDS. J Infect Dis. 1994;170(1):198-202. doi: 10.1093/infdis/170.1.198. PubMed PMID: 8014498.

35. Crepaz N, Hess KL, Purcell DW, Hall HI. Estimating national rates of HIV infection among MSM, persons who inject drugs, and heterosexuals in the United States. AIDS. 2019;33(4):701-8. doi: 10.1097/QAD.0000000000002111. PubMed PMID: 30585840.

36. Services. USHaH. America's HIV Epidemic Analysis Dashboard (AHEAD) 2019. Available from: <https://ahead.hiv.gov/>.

37. Sullivan P, Woodyatt C, Koski C, Pembleton E, McGuinness P, Taussig J, et al. A data visualization and dissemination resource to support HIV prevention and care at the local level: analysis and uses of the AIDSVu Public Data Resource. Journal of medical Internet research. 2020;22(10).

38. Siegler AJ, Mouhanna F, Giler RM, Weiss K, Pembleton E, Guest J, et al. The prevalence of pre-exposure prophylaxis use and the pre-exposure prophylaxis-to-need ratio in the fourth quarter of 2017, United States. Ann Epidemiol. 2018;28(12):841-9. doi: 10.1016/j.annepidem.2018.06.005. PubMed PMID: 29983236; PubMed Central PMCID: PMCPMC6286209.

39. Prevention. CfDCa. Monitoring selected national HIV prevention and care objectives by using HIV surveillance data—United States and 6 dependent areas, 2018. 2020.

40. Liu AY, Cohen SE, Vittinghoff E, Anderson PL, Doblecki-Lewis S, Bacon O, et al. Preexposure Prophylaxis for HIV Infection Integrated With Municipal- and Community-Based Sexual Health Services. JAMA Intern Med. 2016;176(1):75-84. doi: 10.1001/jamainternmed.2015.4683. PubMed PMID: 26571482; PubMed Central PMCID: PMCPMC5042323.

41. Blumenthal J, Jain S, He F, Amico KR, Kofron R, Ellorin E, et al. Results from a Pre-exposure Prophylaxis Demonstration Project for At-risk Cisgender Women in the United States. Clin Infect Dis. 2021;73(7):1149-56. doi: 10.1093/cid/ciab328. PubMed PMID: 33864370; PubMed Central PMCID: PMCPMC8492205.

42. Huang YA, Tao G, Smith DK, Hoover KW. Persistence With Human Immunodeficiency Virus Pre-exposure Prophylaxis in the United States, 2012-2017. Clin Infect Dis. 2021;72(3):379-85. doi: 10.1093/cid/ciaa037. PubMed PMID: 33527117.

43. Marcus JL, Hurley LB, Hare CB, Nguyen DP, Phengrasamy T, Silverberg MJ, et al. Preexposure Prophylaxis for HIV Prevention in a Large Integrated Health Care System: Adherence, Renal Safety, and Discontinuation. J Acquir Immune Defic Syndr. 2016;73(5):540-6. doi: 10.1097/QAI.0000000000001129. PubMed PMID: 27851714; PubMed Central PMCID: PMCPMC5424697.

44. Centers for Disease Control and Prevention. Estimated HIV incidence and prevalence in the United States, 2014–2018. 2020.

45. Supervie V, Breban R. Brief Report: Per Sex-Act Risk of HIV Transmission Under Antiretroviral Treatment: A Data-Driven Approach. J Acquir Immune Defic Syndr. 2018;79(4):440-4. doi: 10.1097/QAI.0000000000001845. PubMed PMID: 30179982.

46. Wilson DP, Law MG, Grulich AE, Cooper DA, Kaldor JM. Relation between HIV viral load and infectiousness: a model-based analysis. The Lancet. 2008;372(9635):314-20. doi: 10.1016/s0140-6736(08)61115-0.

47. Bellan SE, Dushoff J, Galvani AP, Meyers LA. Reassessment of HIV-1 acute phase infectivity: accounting for heterogeneity and study design with simulated cohorts. PLoS Med. 2015;12(3):e1001801. doi: 10.1371/journal.pmed.1001801. PubMed PMID: 25781323; PubMed Central PMCID: PMCPMC4363602.

48. Infectious Disease Prevention and Control HMtQiRoC. HIV Transmission Risk: A Summary of the Evidence. 2012.

49. Leynaert B, Downs AM, de Vincenzi I. Heterosexual transmission of human immunodeficiency virus: variability of infectivity throughout the course of infection. European Study Group on Heterosexual Transmission of HIV. Am J Epidemiol. 1998;148(1):88-96. doi: 10.1093/oxfordjournals.aje.a009564. PubMed PMID: 9663408.

50. Varghese B, Maher JE, Peterman TA, Branson BM, Steketee RW. Reducing the Risk of Sexual HIV Transmission: Quantifying the Per-Act Risk for HIV on the Basis of Choice of Partner, Sex Act, and Condom Use. Sex Transm Dis. 2002;29(1):38-43.

51. Weller SC, Davis-Beaty K. Condom effectiveness in reducing heterosexual HIV transmission. Cochrane Database of Systematic Reviews. 2002. doi: 10.1002/14651858.cd003255.

52. Smith DK, Herbst JH, Zhang X, Rose CE. Condom effectiveness for HIV prevention by consistency of use among men who have sex with men in the United States. J Acquir Immune Defic Syndr. 2015;68(3):337-44. doi: 10.1097/QAI.0000000000000461. PubMed PMID: 25469526.

53. Grant RM, Anderson PL, McMahan V, Liu A, Amico KR, Mehrotra M, et al. Uptake of pre-exposure prophylaxis, sexual practices, and HIV incidence in men and transgender women who have sex with men: a cohort study. The Lancet Infectious Diseases. 2014;14(9):820-9. doi: 10.1016/s1473-3099(14)70847-3.

54. Bernstein KT, Marcus JL, Nieri G, Philip SS, Klausner JD. Rectal gonorrhea and chlamydia reinfection is associated with increased risk of HIV seroconversion. J Acquir Immune Defic Syndr. 2010;53(4):537-43. doi: 10.1097/QAI.0b013e3181c3ef29. PubMed PMID: 19935075.

55. Wand H, Ramjee G. Biological impact of recurrent sexually transmitted infections on HIV seroconversion among women in South Africa: results from frailty models. J Int AIDS Soc. 2015;18:19866. doi: 10.7448/IAS.18.1.19866. PubMed PMID: 25912181; PubMed Central PMCID: PMCPMC4410128.

56. Houlihan CF, Larke NL, Watson-Jones D, Smith-McCune KK, Shiboski S, Gravitt PE, et al. Human papillomavirus infection and increased risk of HIV acquisition. A systematic review and meta-analysis. AIDS. 2012;26(17):2211-22. doi: 10.1097/QAD.0b013e328358d908. PubMed PMID: 22874522; PubMed Central PMCID: PMCPMC3831022.

57. Mwatelah R, McKinnon LR, Baxter C, Abdool Karim Q, Abdool Karim SS. Mechanisms of sexually transmitted infection-induced inflammation in women: implications for HIV risk. J Int AIDS Soc. 2019;22 Suppl 6:e25346. doi: 10.1002/jia2.25346. PubMed PMID: 31468677; PubMed Central PMCID: PMCPMC6715949.

58. Millett GA, Peterson JL, Wolitski RJ, Stall R. Greater risk for HIV infection of black men who have sex with men: a critical literature review. Am J Public Health. 2006;96(6):1007-19. doi: 10.2105/AJPH.2005.066720. PubMed PMID: 16670223; PubMed Central PMCID: PMCPMC1470628.

59. Goodreau SM, Rosenberg ES, Jenness SM, Luisi N, Stansfield SE, Millett GA, et al. Sources of racial disparities in HIV prevalence in men who have sex with men in Atlanta, GA, USA: a modelling study. The Lancet HIV. 2017;4(7):e311-e20. doi: 10.1016/s2352-3018(17)30067-x.

60. Tillerson K. Explaining racial disparities in HIV/AIDS incidence among women in the U.S.: a systematic review. Stat Med. 2008;27(20):4132-43. doi: 10.1002/sim.3224. PubMed PMID: 18551508; PubMed Central PMCID: PMCPMC2684462.

61. Mustanski B, Morgan E, D'Aquila R, Birkett M, Janulis P, Newcomb ME. Individual and Network Factors Associated With Racial Disparities in HIV Among Young Men Who Have Sex With Men: Results From the RADAR Cohort Study. J Acquir Immune Defic Syndr. 2019;80(1):24-30. doi: 10.1097/QAI.0000000000001886. PubMed PMID: 30365451; PubMed Central PMCID: PMCPMC6289601.

62. Oster AM, Wiegand RE, Sionean C, Miles IJ, Thomas PE, Melendez-Morales L, et al. Understanding disparities in HIV infection between black and white MSM in the United States. AIDS. 2011;25(8):1103-12. doi: 10.1097/QAD.0b013e3283471efa. PubMed PMID: 21505305.

63. Morris M, Kurth AE, Hamilton DT, Moody J, Wakefield S. Concurrent partnerships and HIV prevalence disparities by race: linking science and public health practice. Am J Public Health. 2009;99(6):1023-31. doi: 10.2105/AJPH.2008.147835. PubMed PMID: 19372508; PubMed Central PMCID: PMCPMC2679771.
