# Supplementary material for: Achieving the “Ending the HIV Epidemic in the U.S.” incidence reduction goals among at-risk populations in the South
Source: BMC Public Health. 2023 Apr 20;23:716. doi: 10.1186/s12889-023-15563-5 (PMC10116101; doi:10.1186/s12889-023-15563-5)
Supplement: Supplementary file 2 — Additional file 2: Supplemental figures. [file 12889_2023_15563_MOESM2_ESM.docx]

**Figure S1:** Annual HIV incidence per 100,000 individuals and 80% simulation intervals over 8 years with simultaneously improved antiretroviral therapy (ART) and pre-exposure prophylaxis (PrEP) coverage


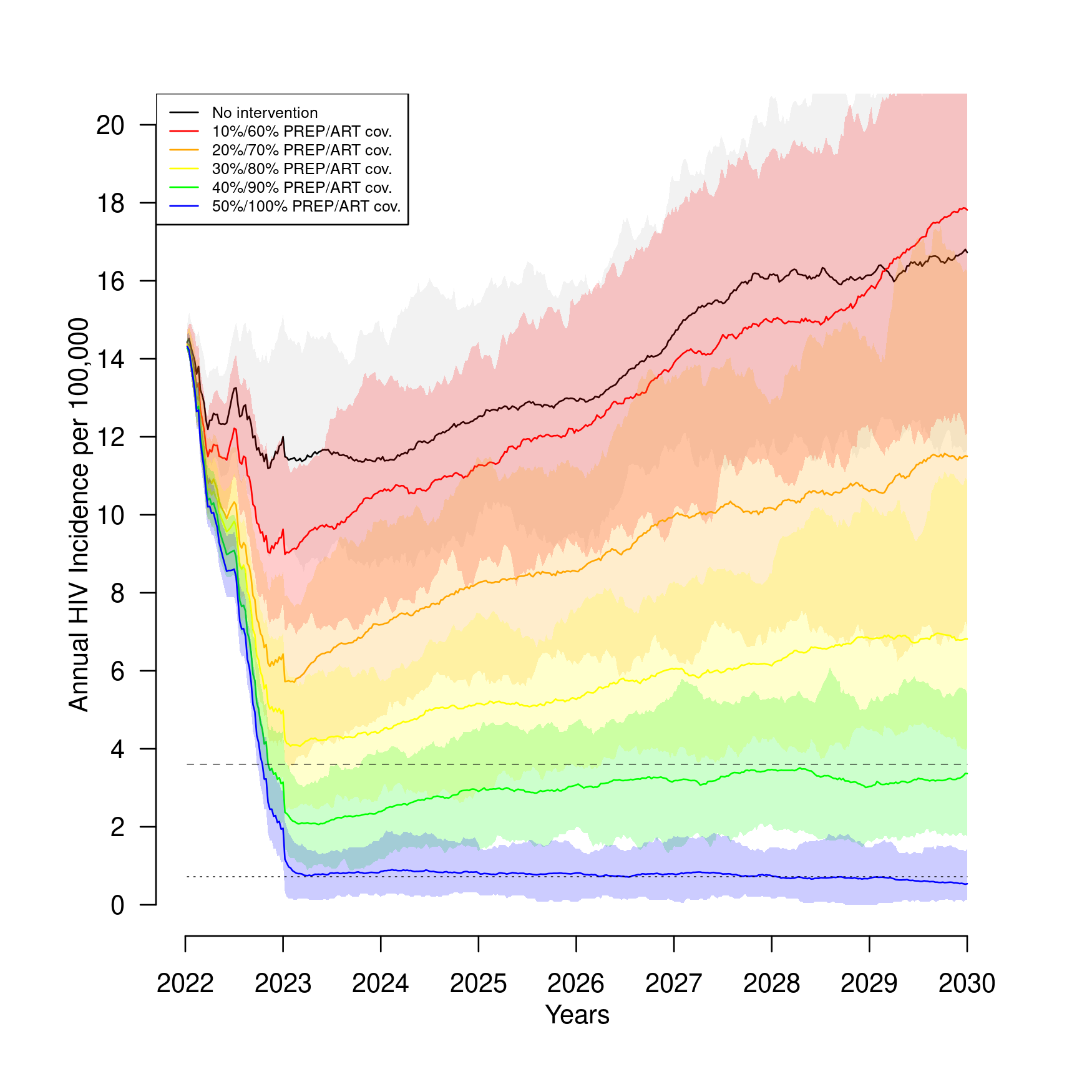


Note: Dashed lines indicate EHE targets

**Figure S2:** HIV incidence among Non-Hispanic Black MSM over 8 years following fixed changes in antiretroviral therapy coverage from

60% to 100% among nine demographic groups and all demographic groups combined.


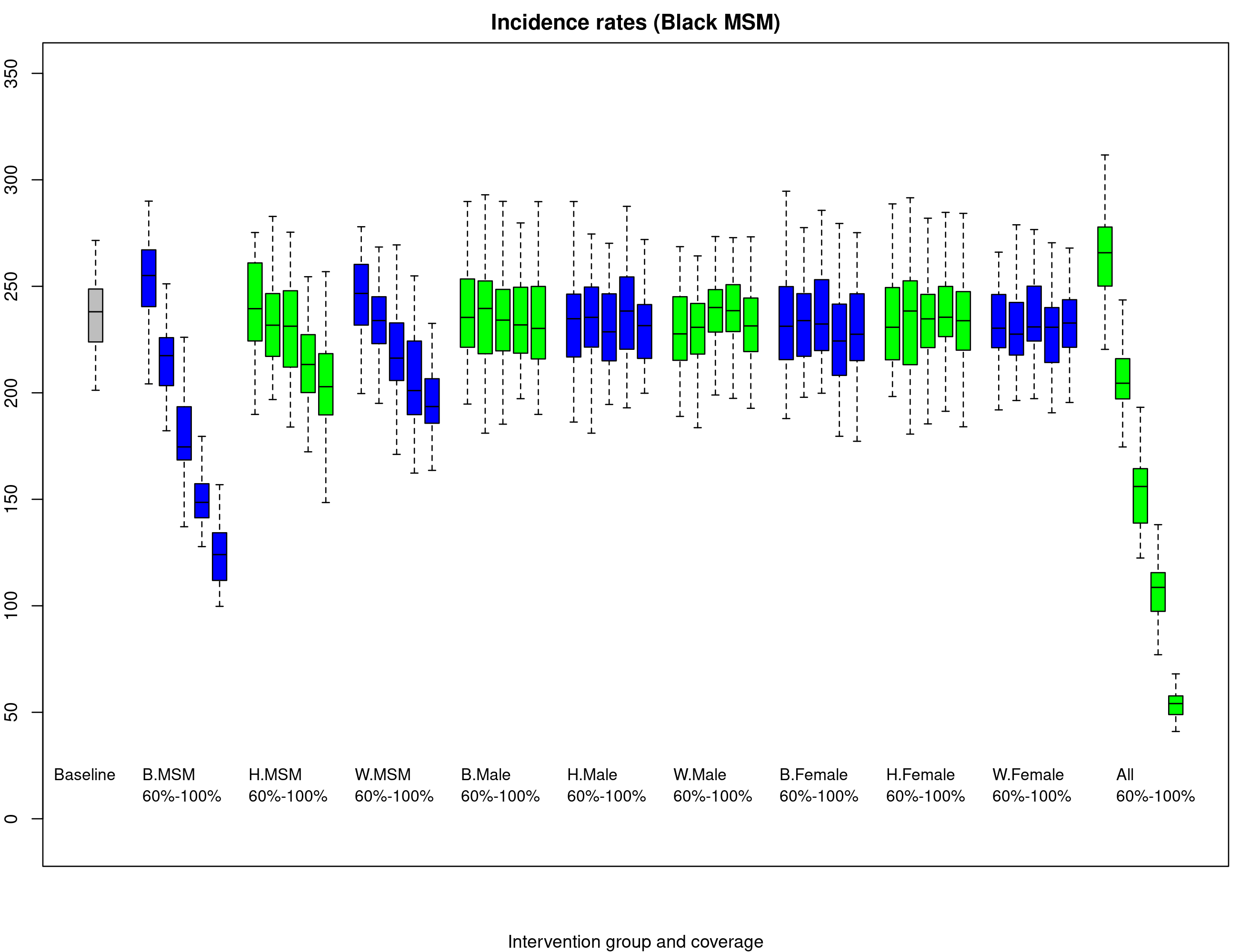


Notes: each boxplot is the combined results for 50 simulations and represents outcomes for one demographic group. All boxplots are for the same demographic group with results changing from baseline across 50 intervention scenarios.

In the x-axis labels B, H and W indicate non-Hispanic Black, Hispanic/Latino and White/Other respectively.

The male category indicates heterosexual males.

**Figure S3:** HIV incidence among Hispanic/Latino MSM over 8 years following fixed increases in antiretroviral therapy coverage from

60% to 100% among nine demographic groups and all demographic groups combined.


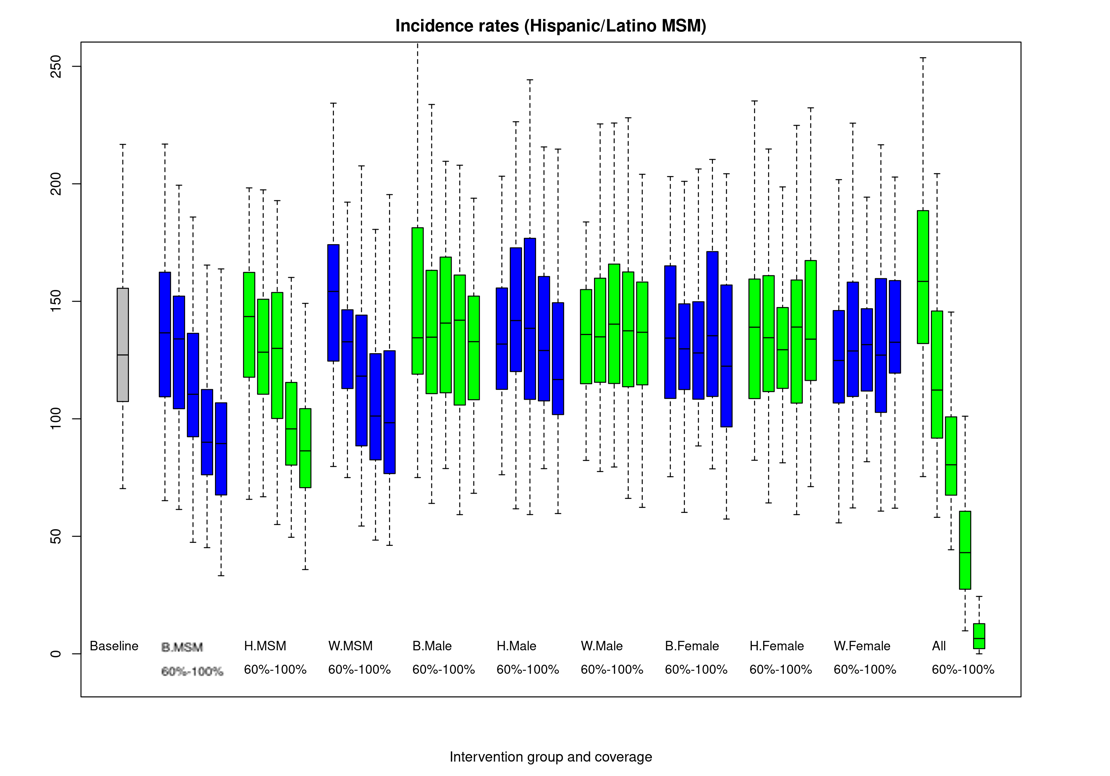


Notes: each boxplot is the combined results for 50 simulations and represents outcomes for one demographic group. All boxplots are for the same demographic group with results changing from baseline across 50 intervention scenarios.

In the x-axis labels B, H and W indicate non-Hispanic Black, Hispanic/Latino and White/Other respectively.

The male category indicates heterosexual males.

**Figure S4:** HIV incidence among White/Other MSM over 8 years following fixed increases in antiretroviral therapy coverage from

60% to 100% among nine demographic groups and all demographic groups combined.


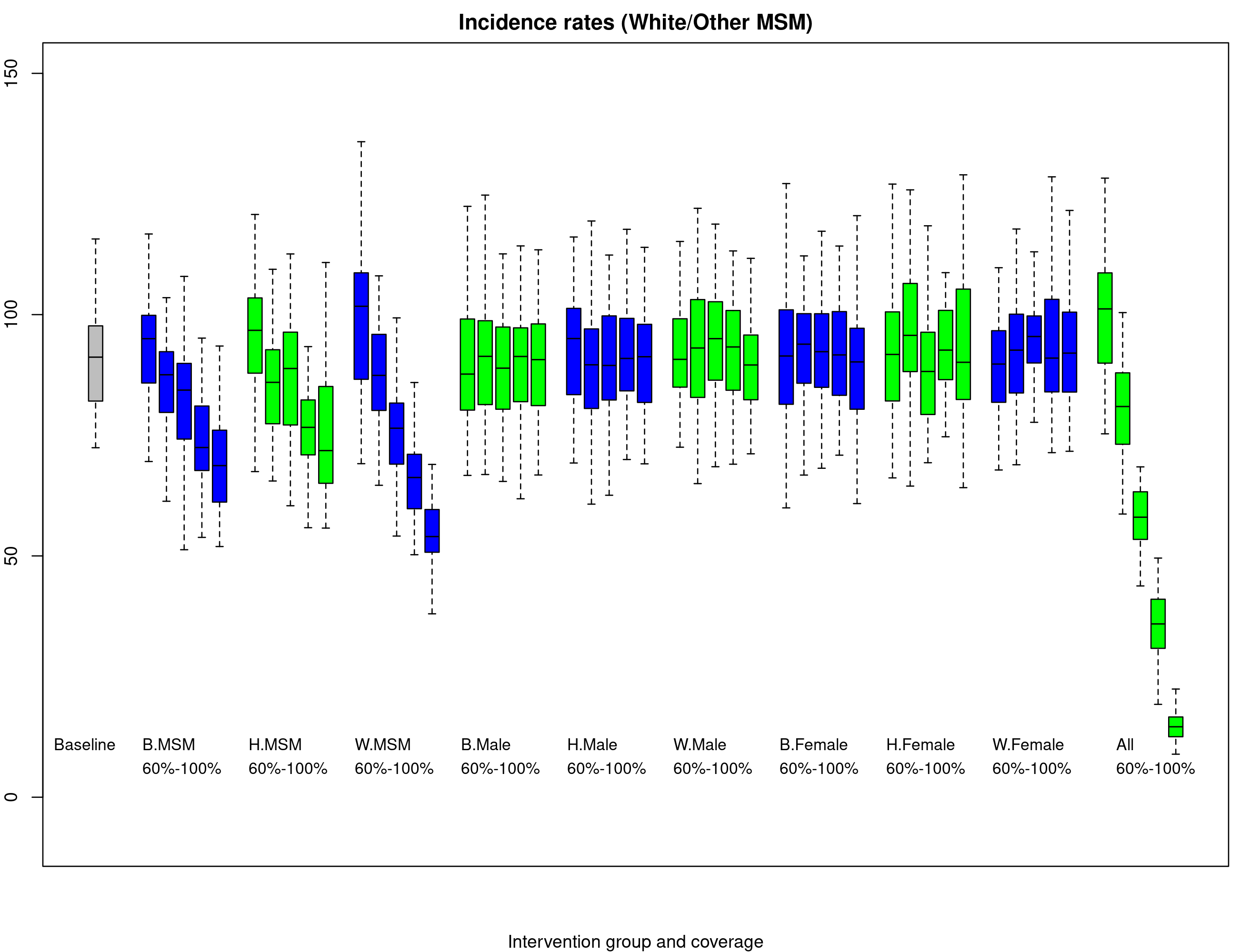


Notes: each boxplot is the combined results for 50 simulations and represents outcomes for one demographic group. All boxplots are for the same demographic group with results changing from baseline across 50 intervention scenarios.

In the x-axis labels B, H and W indicate non-Hispanic Black, Hispanic/Latino and White/Other respectively.

The male category indicates heterosexual males.

**Figure S5:** HIV incidence among non-Hispanic Black heterosexual males over 8 years following fixed increases in antiretroviral therapy coverage from 60% to 100% among nine demographic groups and all demographic groups combined.


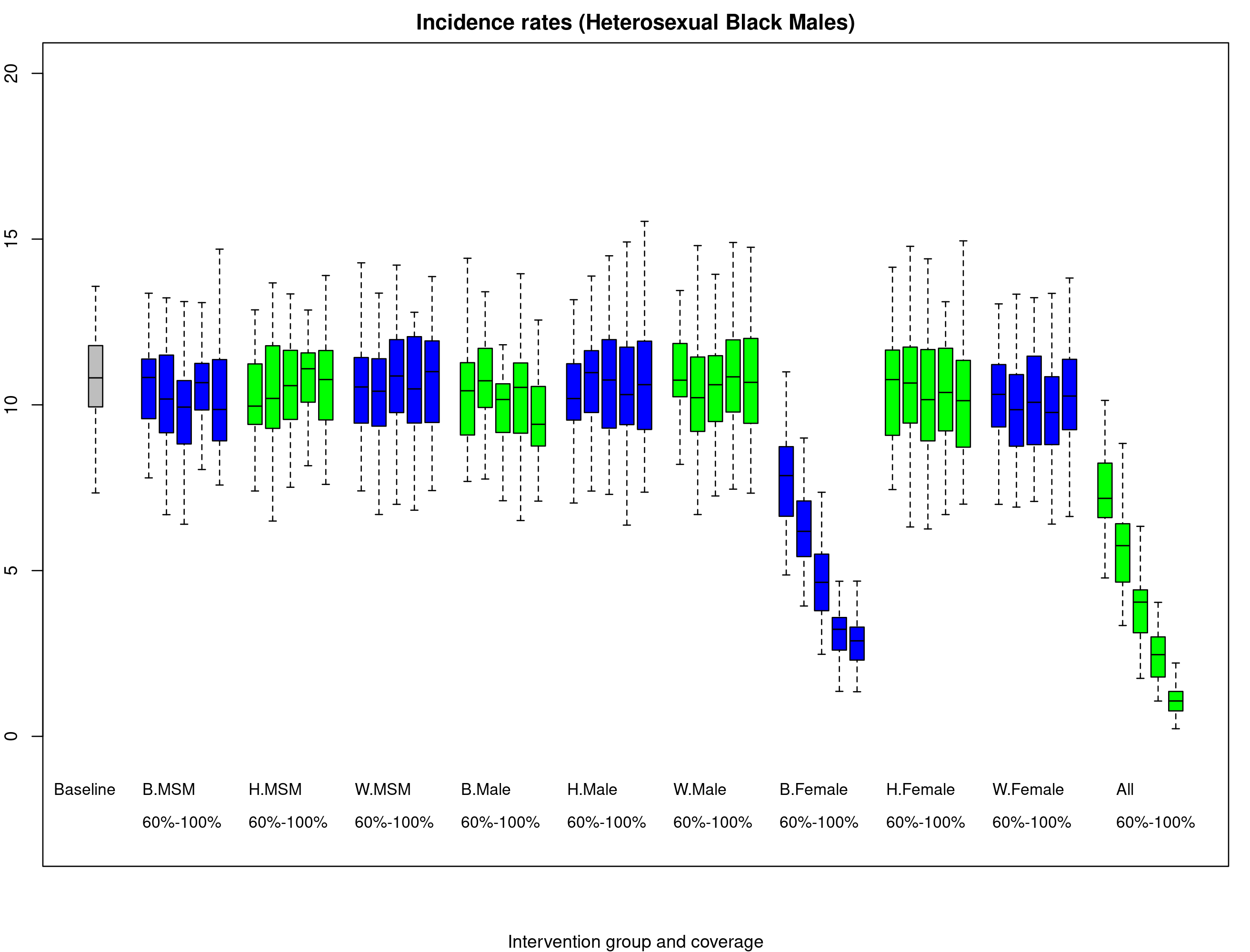


Notes: each boxplot is the combined results for 50 simulations and represents outcomes for one demographic group. All boxplots are for the same demographic group with results changing from baseline across 50 intervention scenarios.

In the x-axis labels B, H and W indicate non-Hispanic Black, Hispanic/Latino and White/Other respectively.

The male category indicates heterosexual males.

**Figure S6:** HIV incidence among Hispanic/Latino heterosexual males over 8 years following fixed increases in antiretroviral therapy coverage from 60% to 100% among nine demographic groups and all demographic groups combined.


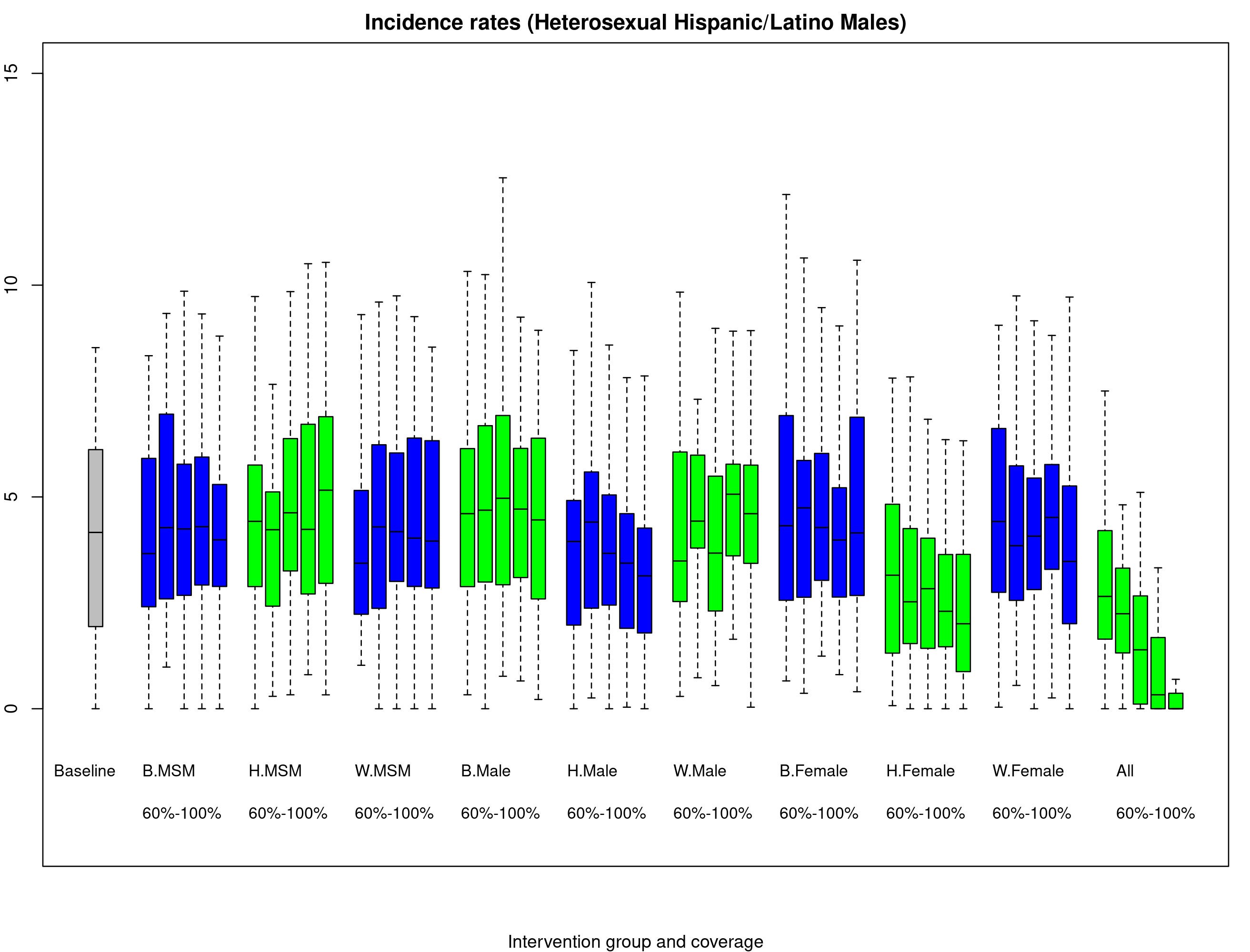


Notes: each boxplot is the combined results for 50 simulations and represents outcomes for one demographic group. All boxplots are for the same demographic group with results changing from baseline across 50 intervention scenarios.

In the x-axis labels B, H and W indicate non-Hispanic Black, Hispanic/Latino and White/Other respectively.

The male category indicates heterosexual males.

**Figure S7:** HIV incidence among White/Other heterosexual males over 8 years following fixed antiretroviral therapy coverage from

60% to 100% among nine demographic groups and all demographic groups combined.


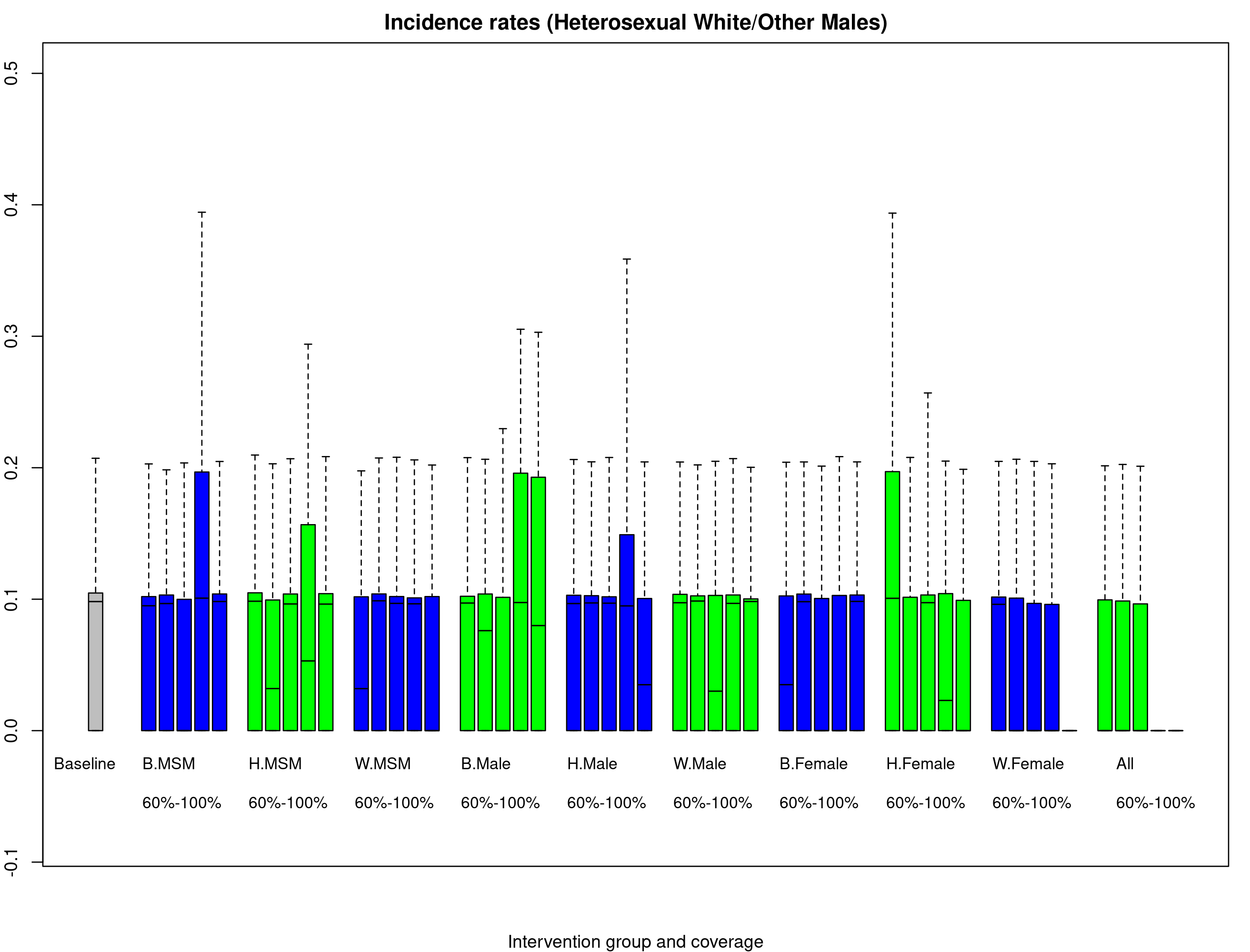


Notes: each boxplot is the combined results for 50 simulations and represents outcomes for one demographic group. All boxplots are for the same demographic group with results changing from baseline across 50 intervention scenario.

In the x-axis labels B, H and W indicate non-Hispanic Black, Hispanic/Latino and White/Other respectively.

The male category indicates heterosexual males.

**Figure S8:** HIV incidence among non-Hispanic Black heterosexual females over 8 years following fixed antiretroviral therapy coverage from

60% to 100% among nine demographic groups and all demographic groups combined.


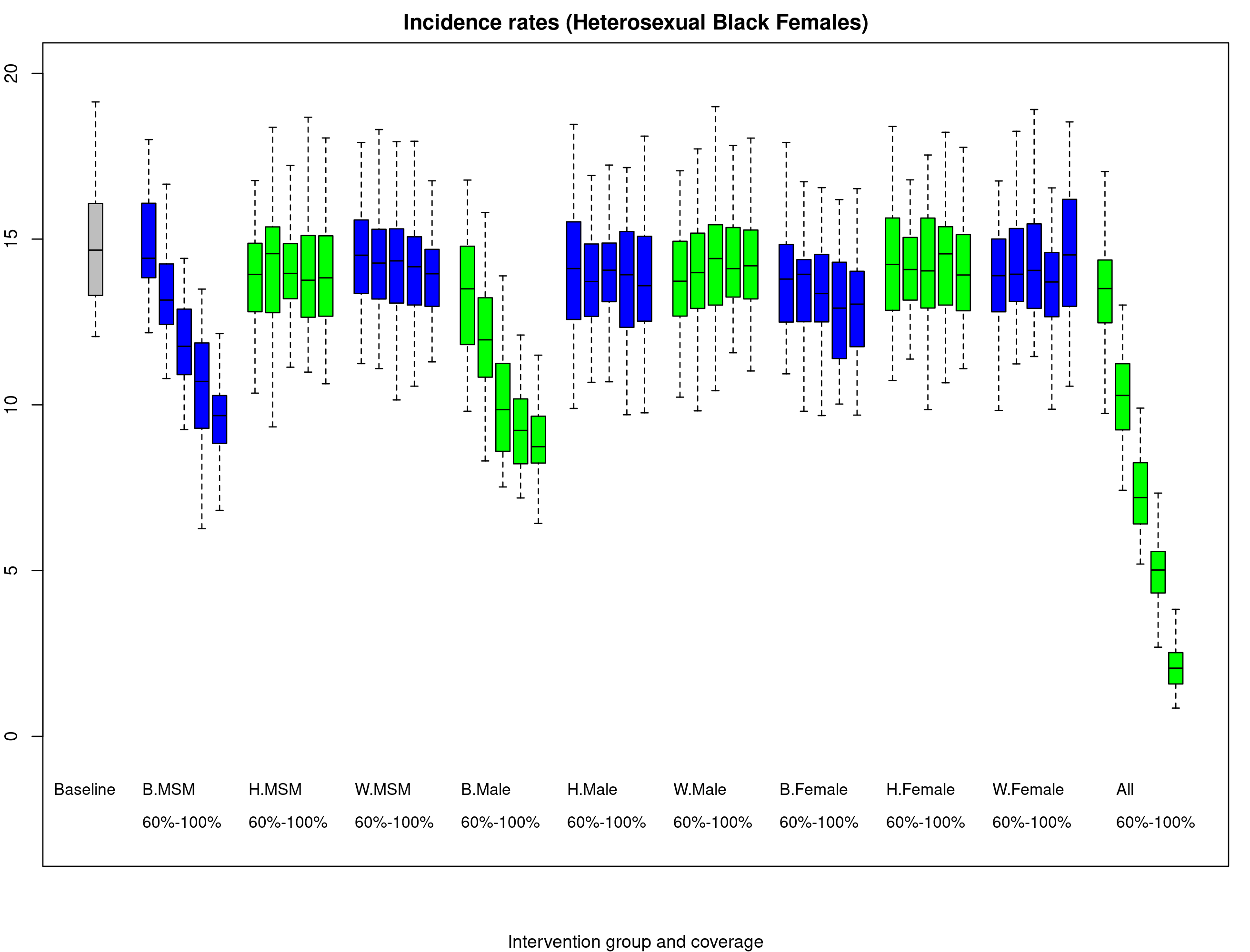


Notes: each boxplot is the combined results for 50 simulations and represents outcomes for one demographic group. All boxplots are for the same demographic group with results changing from baseline across 50 intervention scenarios.

In the x-axis labels B, H and W indicate non-Hispanic Black, Hispanic/Latino and White/Other respectively.

The male category indicates heterosexual males.

**Figure S9:** HIV incidence among Hispanic/Latina heterosexual females over 8 years following fixed antiretroviral therapy coverage from

60% to 100% among nine demographic groups and all demographic groups combined.


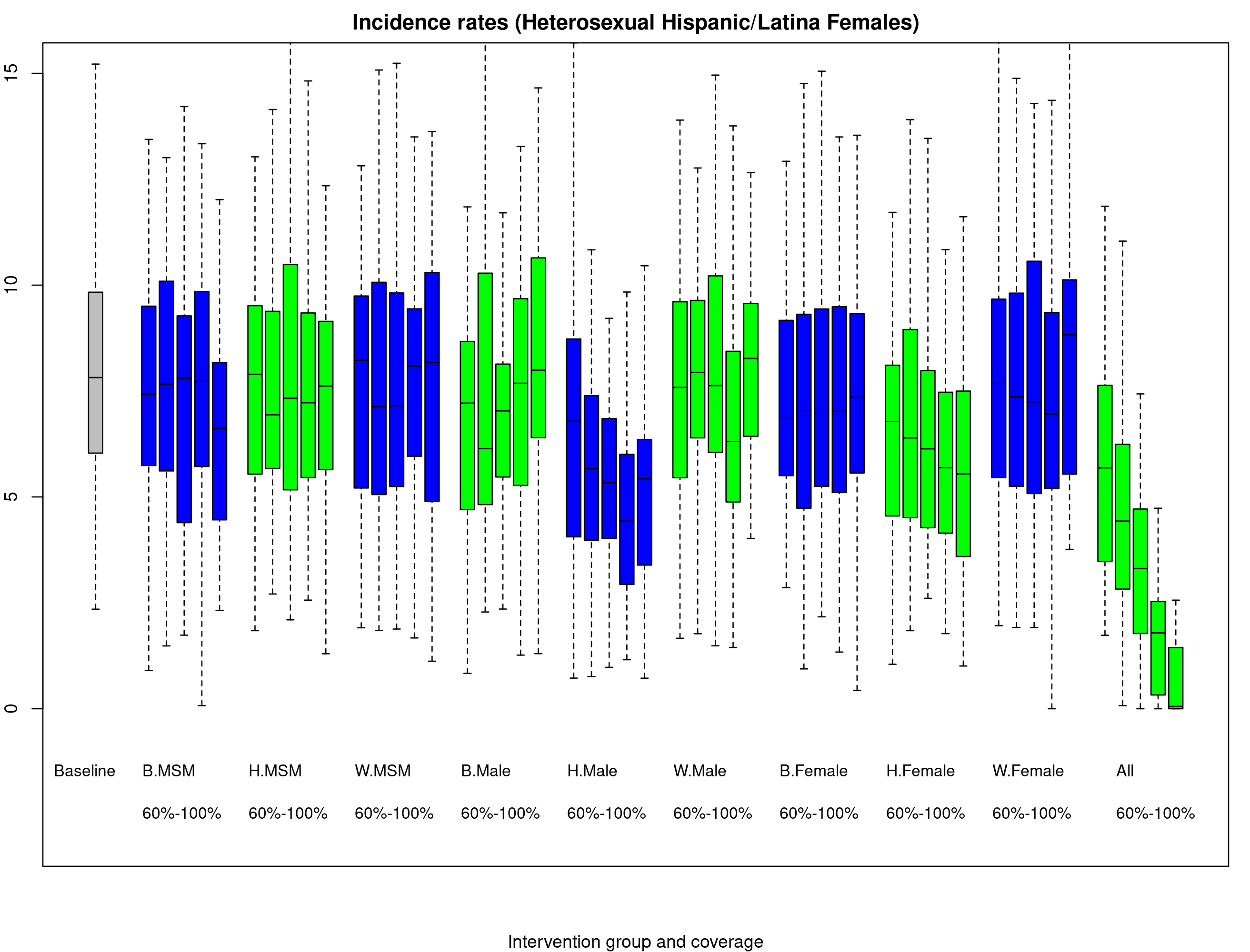


Notes: each boxplot is the combined results for 50 simulations and represents outcomes for one demographic group. All boxplots are for the same demographic group with results changing from baseline across 50 intervention scenario.

In the x-axis labels B, H and W indicate non-Hispanic Black, Hispanic/Latino and White/Other respectively.

The male category indicates heterosexual males.

**Figure S10:** HIV incidence among White/Other heterosexual females over 8 years following fixed antiretroviral therapy coverage from

60% to 100% among nine demographic groups and all demographic groups combined.


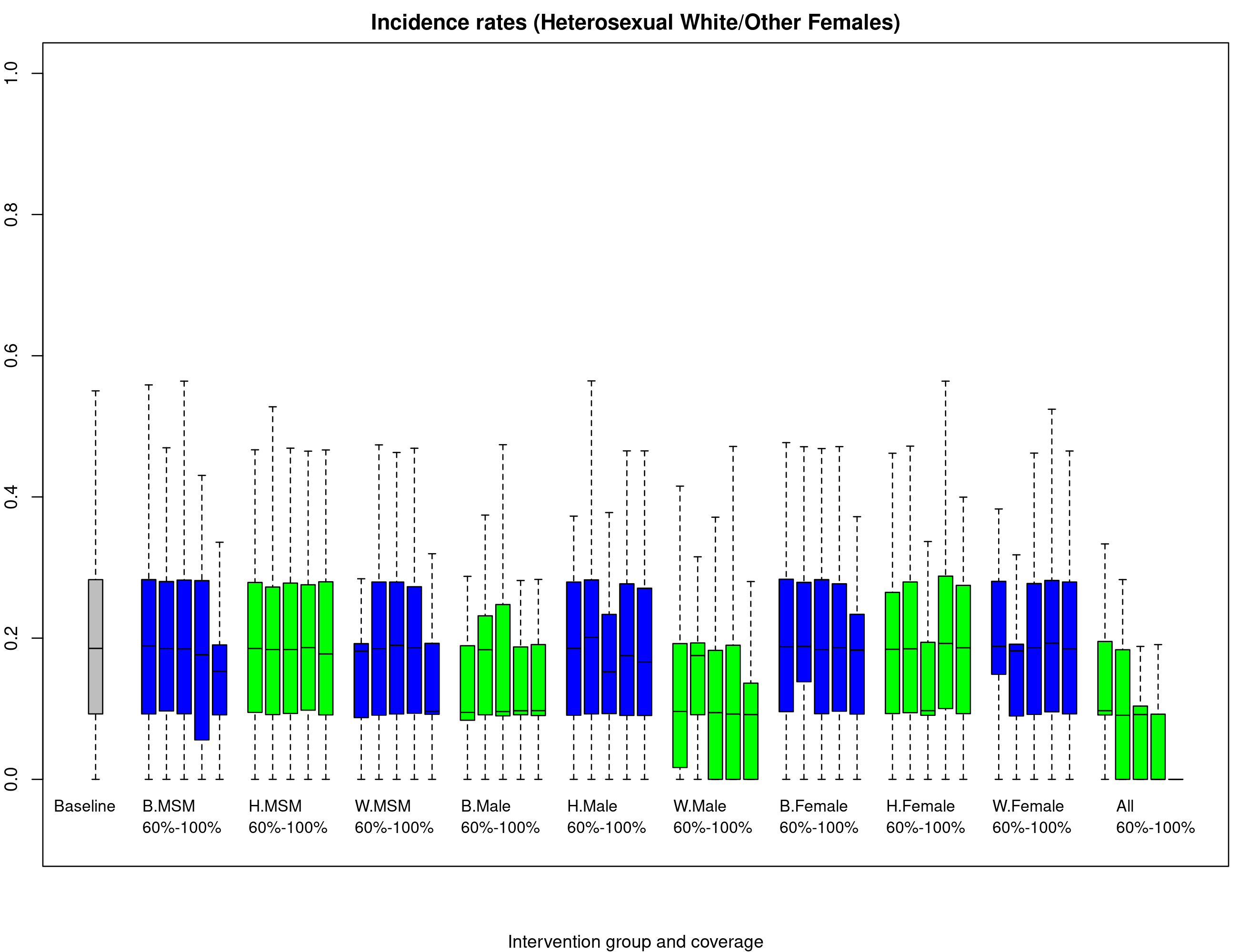


Notes: each boxplot is the combined results for 50 simulations and represents outcomes for one demographic group. All boxplots are for the same demographic group with results changing from baseline across 50 intervention scenarios.

In the x-axis labels B, H and W indicate non-Hispanic Black, Hispanic/Latino and White/Other respectively.

The male category indicates heterosexual males.

**Figure S11:** HIV incidence among Non-Hispanic Black MSM over 8 years following changes in pre-exposure prophylaxis coverage from

10% to 50% among eligible individuals in nine demographic groups and all demographic groups combined.


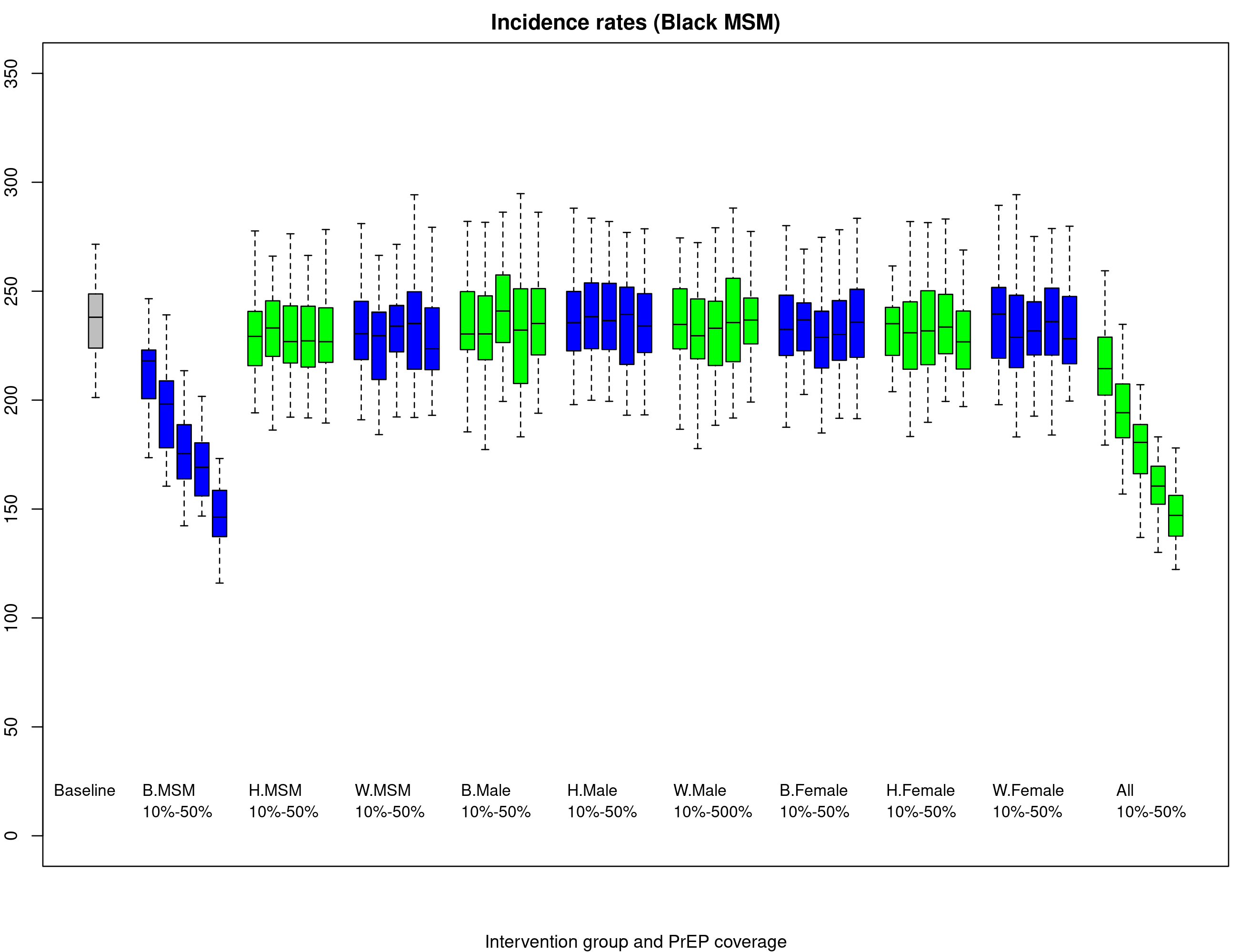


Notes: each boxplot is the combined results for 50 simulations and represents outcomes for one demographic group. All boxplots are for the same demographic group with results changing from baseline across 50 intervention scenarios results.

In the x-axis labels B, H and W indicate non-Hispanic Black, Hispanic/Latino and White/Other respectively.

The male category indicates heterosexual males

**Figure S12:** HIV incidence among Hispanic/Latino MSM over 8 years following fixed pre-exposure prophylaxis coverage from

10% to 50% among eligible individuals in nine demographic groups and all demographic groups combined.


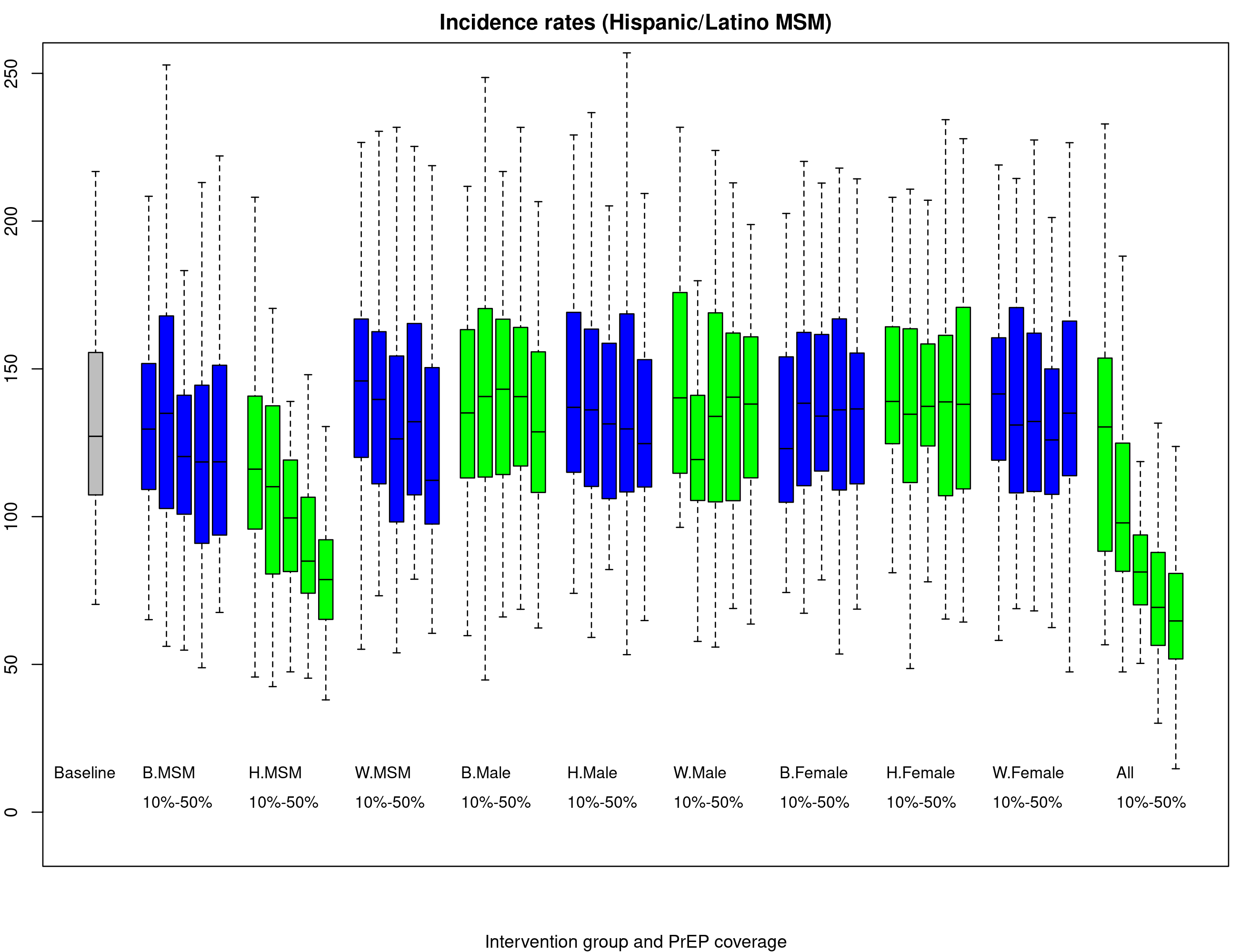


Notes: each boxplot is the combined results for 50 simulations and represents outcomes for one demographic group. All boxplots are for the same demographic group with results changing from baseline across 50 intervention scenarios.

In the x-axis labels B, H and W indicate non-Hispanic Black, Hispanic/Latino and White/Other respectively.

The male category indicates heterosexual males.

**Figure S13:** HIV incidence among White/Other MSM over 8 years following fixed pre-exposure prophylaxis coverage from

10% to 50% among eligible individuals in nine demographic groups and all demographic groups combined.


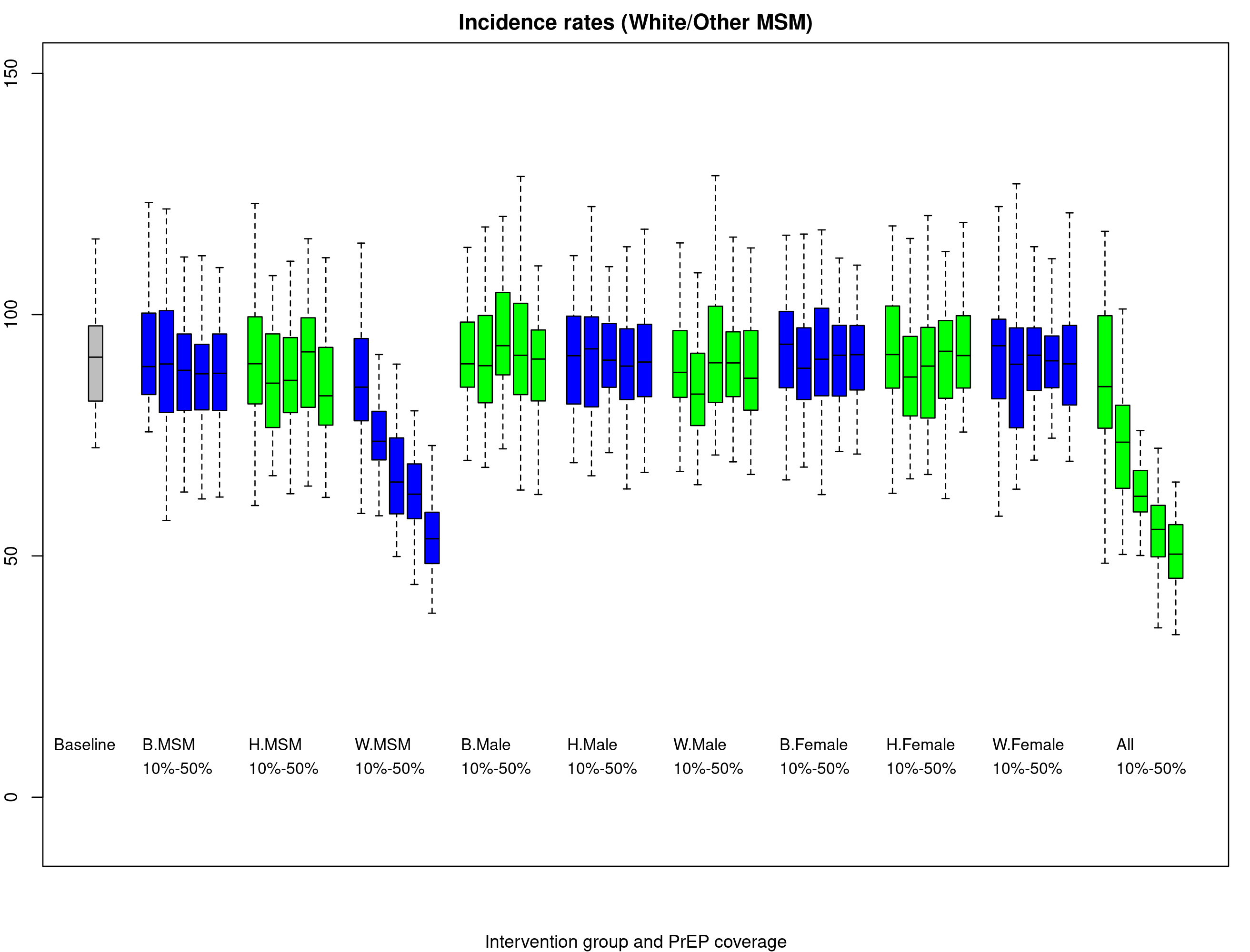


Notes: each boxplot is the combined results for 50 simulations and represents outcomes for one demographic group. All boxplots are for the same demographic group with results changing from baseline across 50 intervention scenarios.

In the x-axis labels B, H and W indicate non-Hispanic Black, Hispanic/Latino and White/Other respectively.

The male category indicates heterosexual males.

**Figure S14:** HIV incidence among non-Hispanic Black heterosexual males over 8 years following fixed pre-exposure prophylaxis coverage from

10% to 50% among eligible individuals in nine demographic groups and all demographic groups combined.


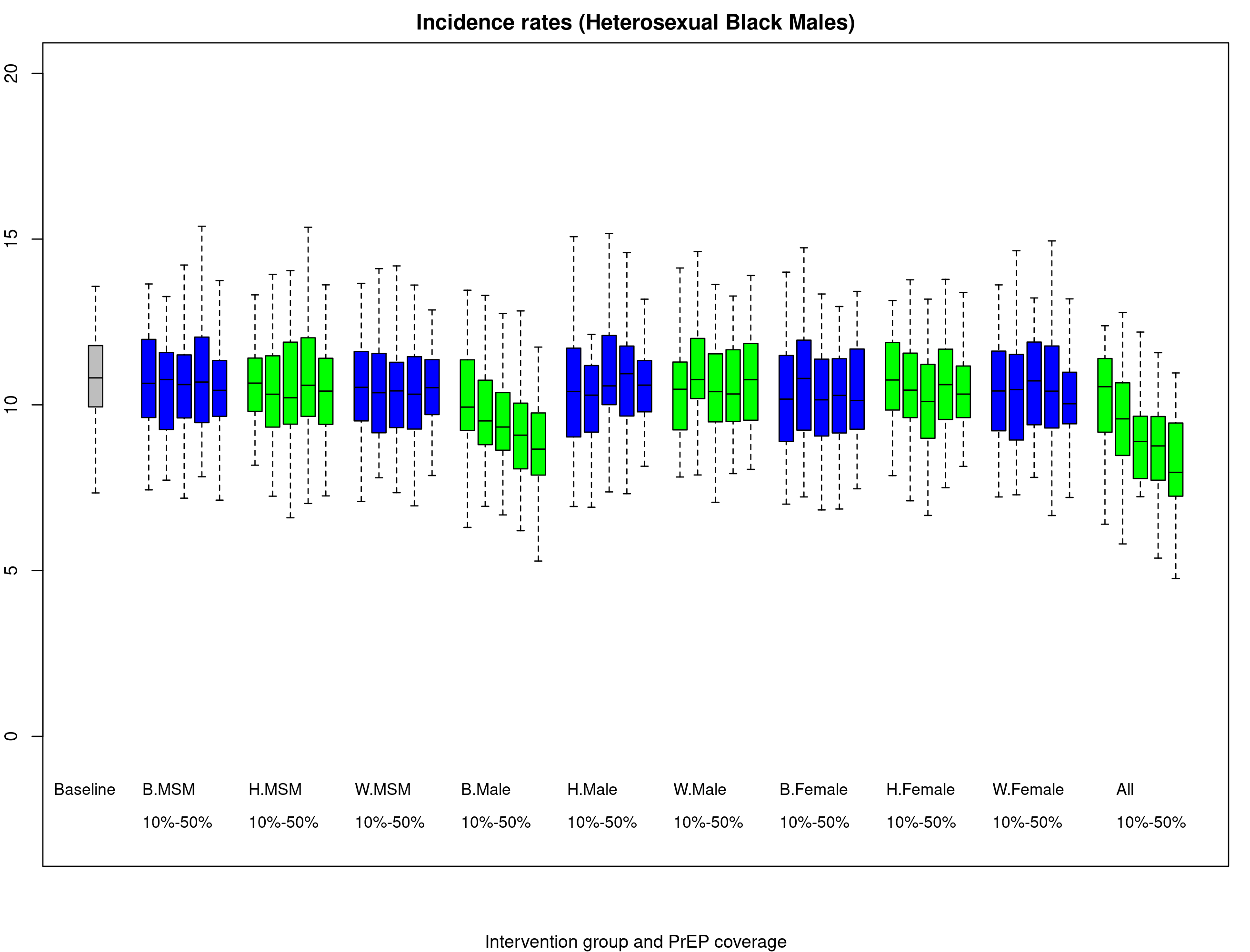


Notes: each boxplot is the combined results for 50 simulations and represents outcomes for one demographic group. All boxplots are for the same demographic group with results changing from baseline across 50 intervention scenarios.

In the x-axis labels B, H and W indicate non-Hispanic Black, Hispanic/Latino and White/Other respectively.

The male category indicates heterosexual males.

**Figure S15:** HIV incidence among Hispanic/Latino heterosexual males over 8 years following fixed pre-exposure prophylaxis coverage from 10% to 50% among eligible individuals in nine demographic groups and all demographic groups combined.


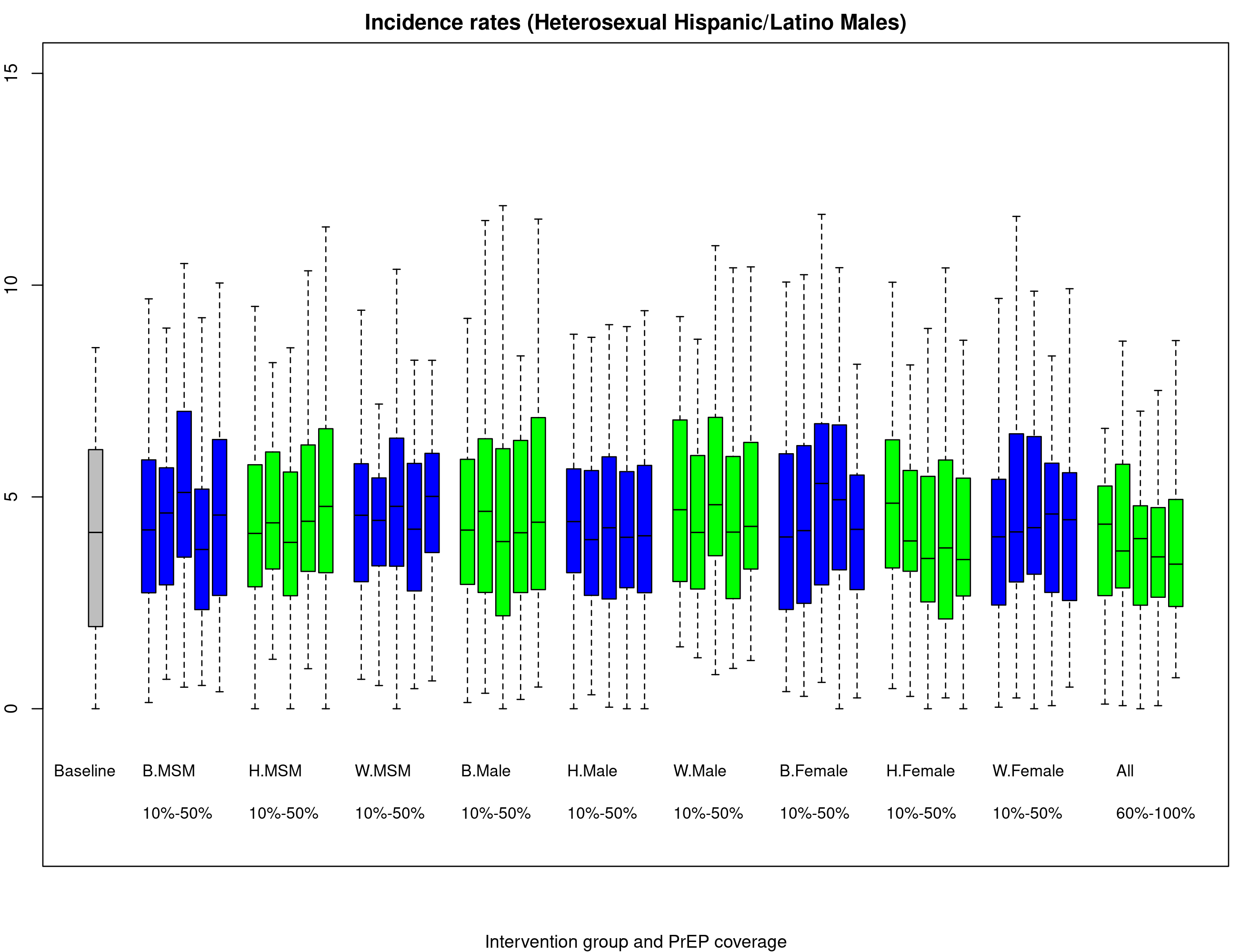


Notes: each boxplot is the combined results for 50 simulations and represents outcomes for one demographic group. All boxplots are for the same demographic group with results changing from baseline across 50 intervention scenarios.

In the x-axis labels B, H and W indicate non-Hispanic Black, Hispanic/Latino and White/Other respectively.

The male category indicates heterosexual males.

**Figure S16:** HIV incidence among White/Other heterosexual males over 8 years following fixed pre-exposure prophylaxis coverage from

10% to 50% among eligible individuals in nine demographic groups and all demographic groups combined.


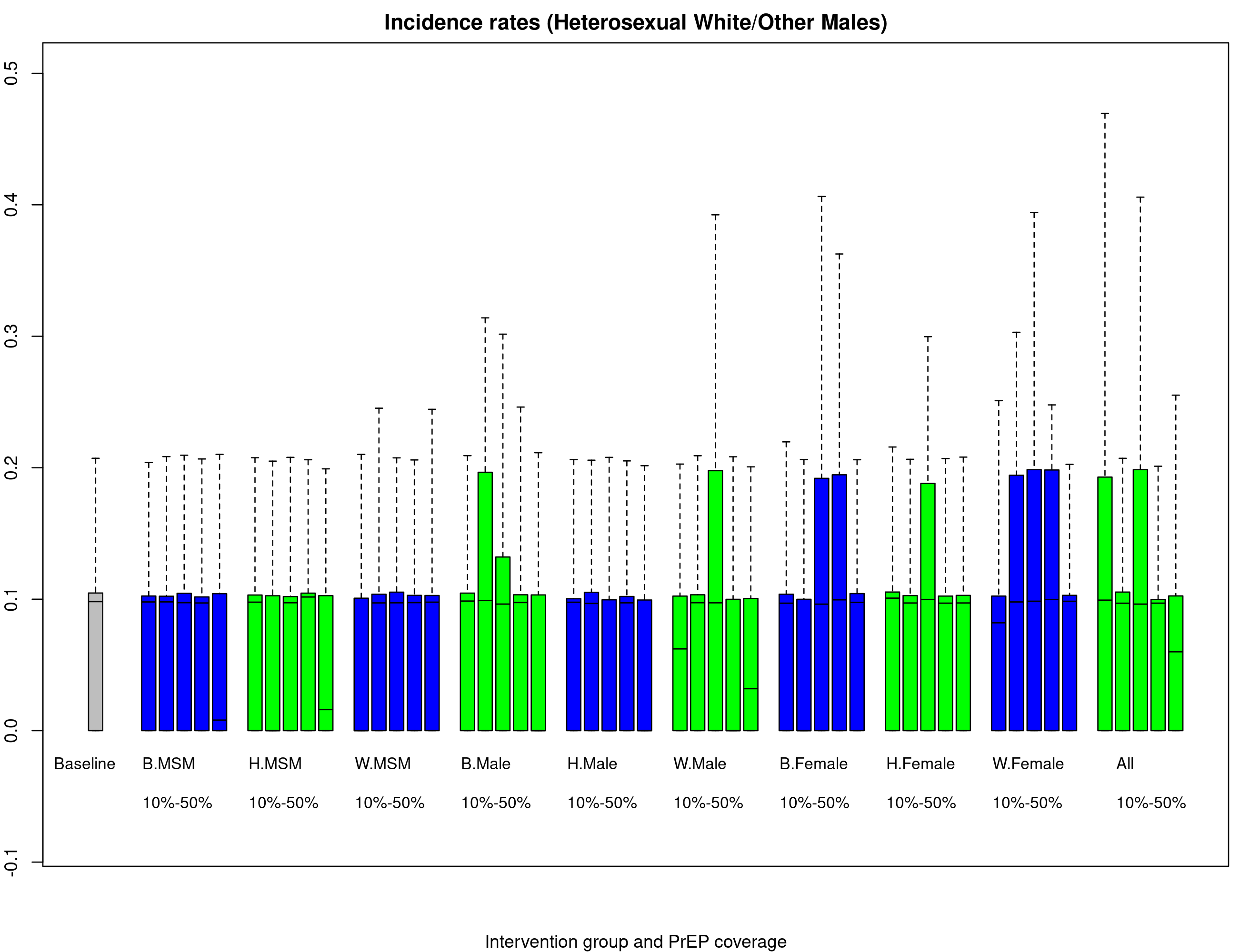


Notes: each boxplot is the combined results for 50 simulations and represents outcomes for one demographic group. All boxplots are for the same demographic group with results changing from baseline across 50 intervention scenarios.

In the x-axis labels B, H and W indicate non-Hispanic Black, Hispanic/Latino and White/Other respectively.

The male category indicates heterosexual males.

**Figure S17:** HIV incidence among non-Hispanic Black heterosexual females over 8 years following fixed pre-exposure prophylaxis coverage from

10% to 50% among eligible individuals in nine demographic groups and all demographic groups combined.


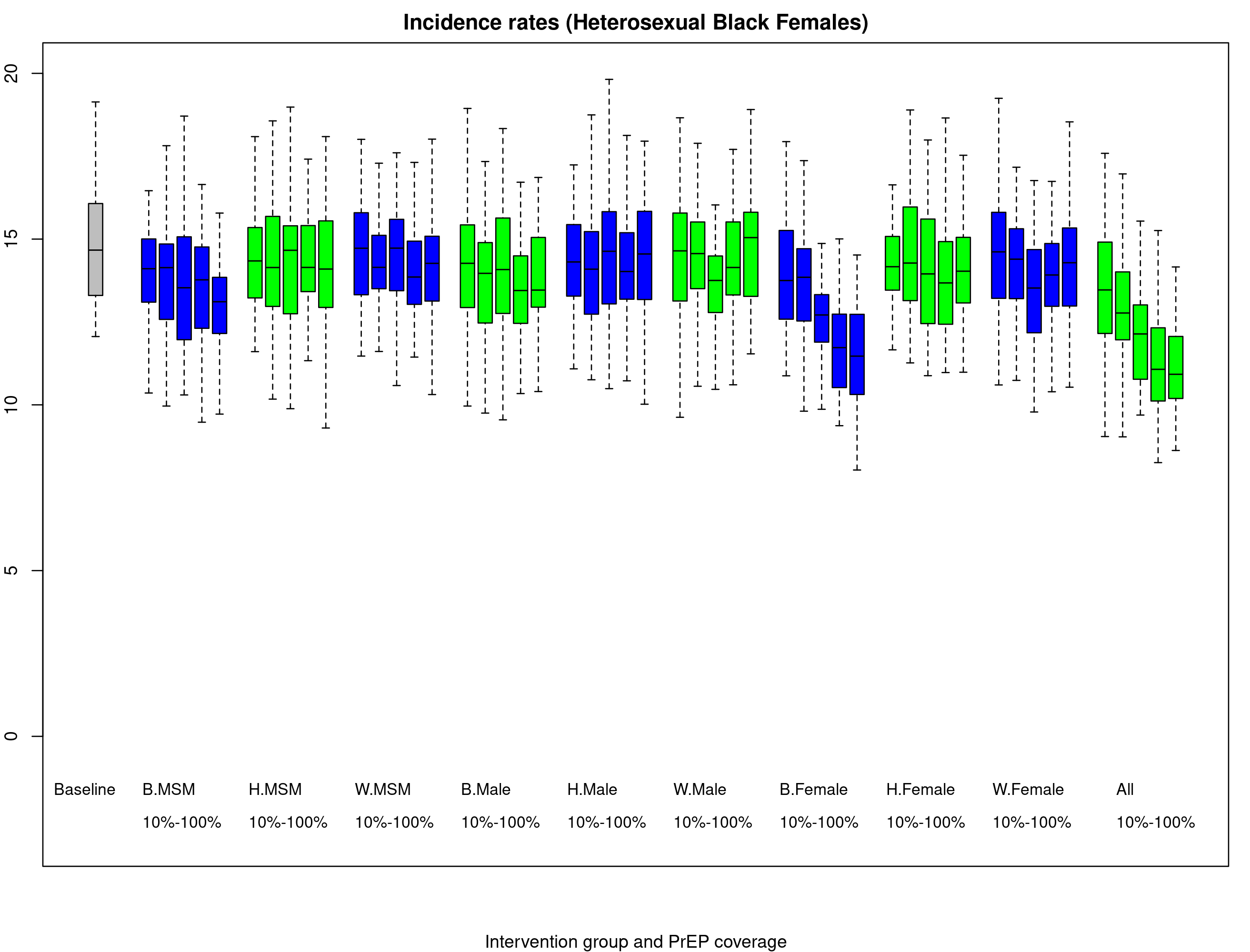


Notes: each boxplot is the combined results for 50 simulations and represents outcomes for one demographic group. All boxplots are for the same demographic group with results changing from baseline across 50 intervention scenarios.

In the x-axis labels B, H and W indicate non-Hispanic Black, Hispanic/Latino and White/Other respectively.

The male category indicates heterosexual male.

**Figure S18:** HIV incidence among Hispanic/Latina heterosexual females over 8 years following fixed pre-exposure prophylaxis coverage from

10% to 50% among eligible individuals in nine demographic groups and all demographic groups combined.


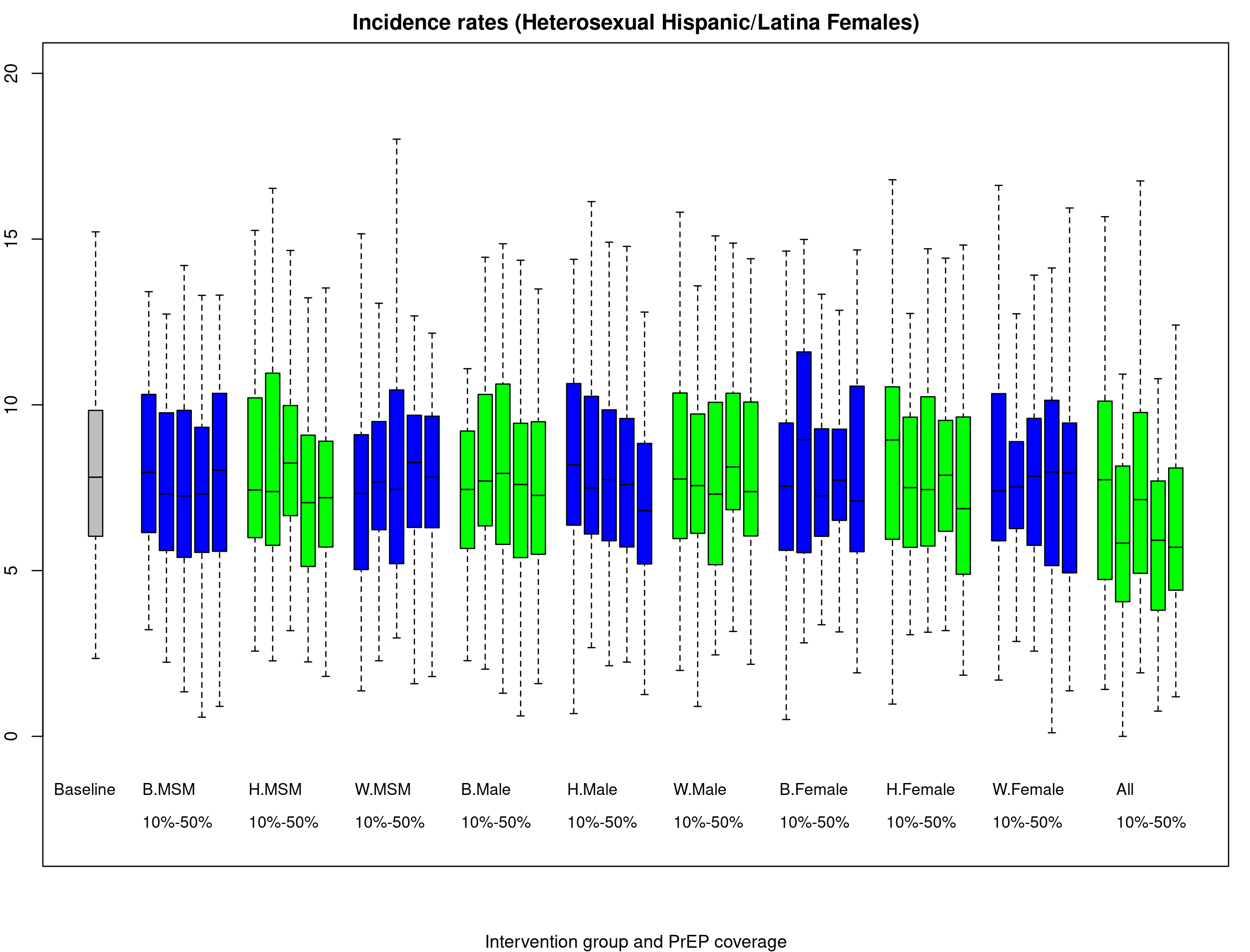


Notes: each boxplot is the combined results for 50 simulations and represents outcomes for one demographic group. All boxplots are for the same demographic group with results changing from baseline across 50 intervention scenarios.

In the x-axis labels B, H and W indicate non-Hispanic Black, Hispanic/Latino and White/Other respectively.

The male category indicates heterosexual males.

**Figure S19:** HIV incidence among White/Other heterosexual females over 8 years following fixed pre-exposure prophylaxis coverage from

10% to 50% among eligible individuals in nine demographic groups and all demographic groups combined.


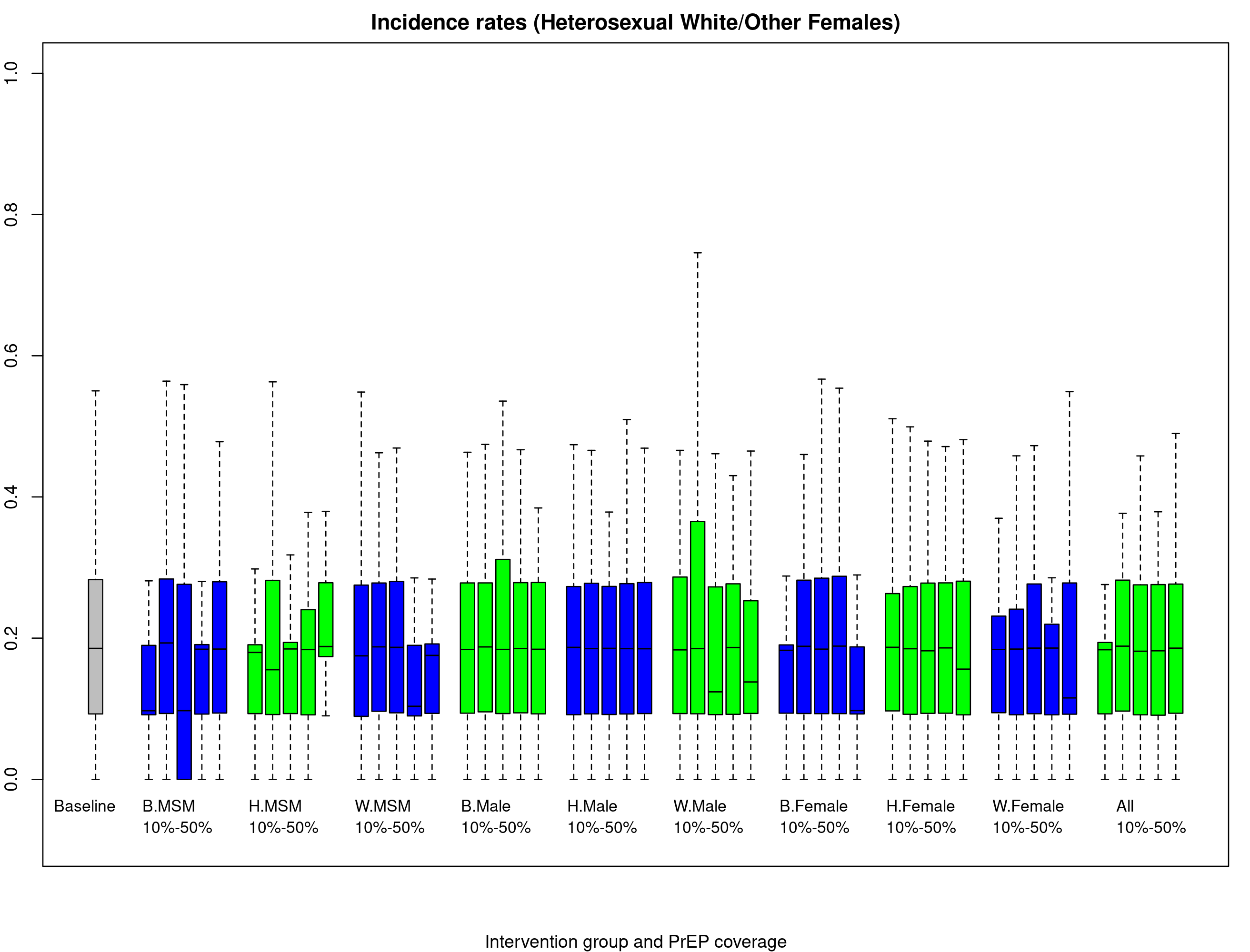


Notes: each boxplot is the combined results for 50 simulations and represents outcomes for one demographic group. All boxplots are for the same demographic group with results changing from baseline across 50 intervention scenarios.

In the x-axis labels B, H and W indicate non-Hispanic Black, Hispanic/Latino and White/Other respectively.

The male category indicates heterosexual males.
